# Design, synthesis, structural characterization, anticancer evaluation, and computational studies of novel quinoline-1,2,3-triazole glycohybrids

**DOI:** 10.1039/d5ra09700b

**Published:** 2026-02-11

**Authors:** Ravendra Kumar, Deepanshi Chauhan, Rakesh Kumar Gupta, Pawan K. Dubey, Divya Kushwaha

**Affiliations:** a Department of Chemistry, MMV, Banaras Hindu University Varanasi-221005 India divyakush.mmvbhu@ac.in; b Centre for Genetic Disorder, Institute of Science, Banaras Hindu University Varanasi-221005 India

## Abstract

A novel series of eleven phenyl-substituted quinoline 1,2,3-triazole glycoconjugates 6(a–k) was synthesized *via* the copper(i)-catalyzed azide-alkyne cycloaddition (CuAAC) reaction between 2-phenyl-substituted quinoline-4-carboxylic acid propargyl ester (4a–4e) and various sugar azides (5a, 5b, and 5c). The obtained compounds were comprehensively characterized by FT-IR, ^1^H NMR, ^13^C NMR, and mass spectrometry. The chemical structures of compounds 6a, 6b, and 6e were further established by single-crystal X-ray diffraction analysis. Their crystal packing was stabilized through a network of intermolecular hydrogen bonds involving C–H⋯N, N–H⋯N, and C–H⋯O (sp^2^, sp^3^) interactions, which were qualitatively analyzed using Hirshfeld surface analysis. *In vitro* anticancer evaluation against the MCF-7 breast cancer cell line revealed that most of the compounds of the series were inactive, while compounds 6c, 6d, and 6f exhibited only weakly cytotoxic activity (IC_50_ values of 108.59–135.95 µM). Molecular docking with estrogen receptor alpha (ERα, PDB ID: 3ERT) revealed good binding affinities (up to −9.1 kcal mol^−1^), comparable to the reference ligand (−9.7 kcal mol^−1^). Furthermore, DFT calculations were performed to evaluate key electronic parameters (HOMO–LUMO energies, reactivity, stability, and charge distribution), offering insight into the compounds' chemical behaviour and electronic properties.

## Abstract 2

Phenyl-substituted quinoline–1,2,3-triazole glycoconjugates were synthesized *via* CuAAC and characterized by spectroscopy and X-ray analysis. Crystal packing, molecular docking, and DFT studies elucidated structural and electronic features.

## Graphical abstract

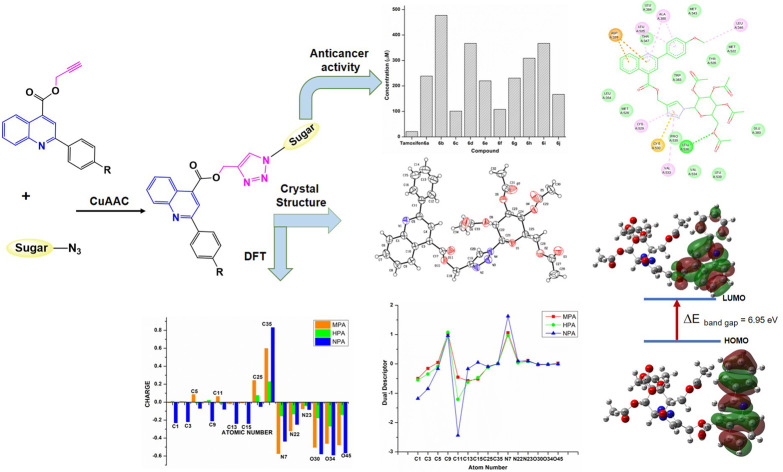

## 1. Introduction

Quinoline derivatives constitute an important class of heterocyclic compounds that have attracted considerable attention due to their broad spectrum of pharmacological activities, including anticancer, antimicrobial, antioxidant, antitubercular, antidiabetic, antimycobacterial, anticonvulsant, anti-Alzheimer, anti-inflammatory, and cardiovascular properties.^1^ Classical synthetic strategies such as the Pfitzinger and Friedländer reactions have long served as fundamental approaches for constructing quinoline frameworks with structural diversity. Owing to the remarkable structural versatility and diverse biological relevance, quinoline-based scaffolds have been extensively investigated in medicinal chemistry for the development of novel therapeutic agents, particularly anticancer drugs.^2^ Quinoline-based compounds are known to exert anticancer activity through multiple mechanisms, including inhibition of tyrosine kinases, cell cycle arrest, suppression of angiogenesis, induction of apoptosis, inhibition of tubulin polymerization, and disruption of cell migration *etc.*^3^ Furthermore, structural modification of the quinoline nucleus has yielded numerous derivatives with enhanced potency, particularly through DNA intercalation and interference with replication processes, emphasizing the significance of quinoline scaffold as versatile pharmacophore for anticancer drug design.^2,3^

To enhance the therapeutic potential of a bioactive heterocycle, molecular hybridization strategies have gained increasing attention, notably through the copper(i)-catalyzed azide-alkyne cycloaddition (CuAAC). This highly efficient and regioselective click reaction enables the reliable conjugation of two structurally and functionally distinct pharmacophoric moieties.^4,5^ The resulting 1,2,3-triazole linker exhibits remarkable chemical stability and acts as a bioisostere for amide and ester linkages, improving molecular polarity, hydrogen-bonding ability, and pharmacokinetic behaviour.^6–8^ Additionally, the conjugation of heterocyclic frameworks with carbohydrates has emerged as an effective approach in modern drug design due to their inherent chirality, hydrophilicity, target selectivity, and critical roles in molecular recognition and cellular processes.^9,10^ Such glycohybrid systems exploit the elevated glucose uptake rate of cancer cells, facilitating selective accumulation within tumour tissues and thereby improving therapeutic efficacy.

Breast cancer remains one of the most prevalent malignancies worldwide and represents the second leading cause of cancer-related mortality, accounting for approximately 24% of all diagnosed cancer cases and 15% of associated deaths.^11^ It is recognized as a heterogeneous disease comprising multiple molecular subtypes including, luminal A, luminal B, human epidermal growth factor (HER2)-positive, and triple-negative.^12^ The MCF-7 cell line, established in 1973, serves as a representative model of luminal A-type breast cancer characterized by estrogen receptor (ERα)– and progesterone receptor (PR)-positive, low invasiveness and limited metastatic potential.^13^

**Fig. 1:**
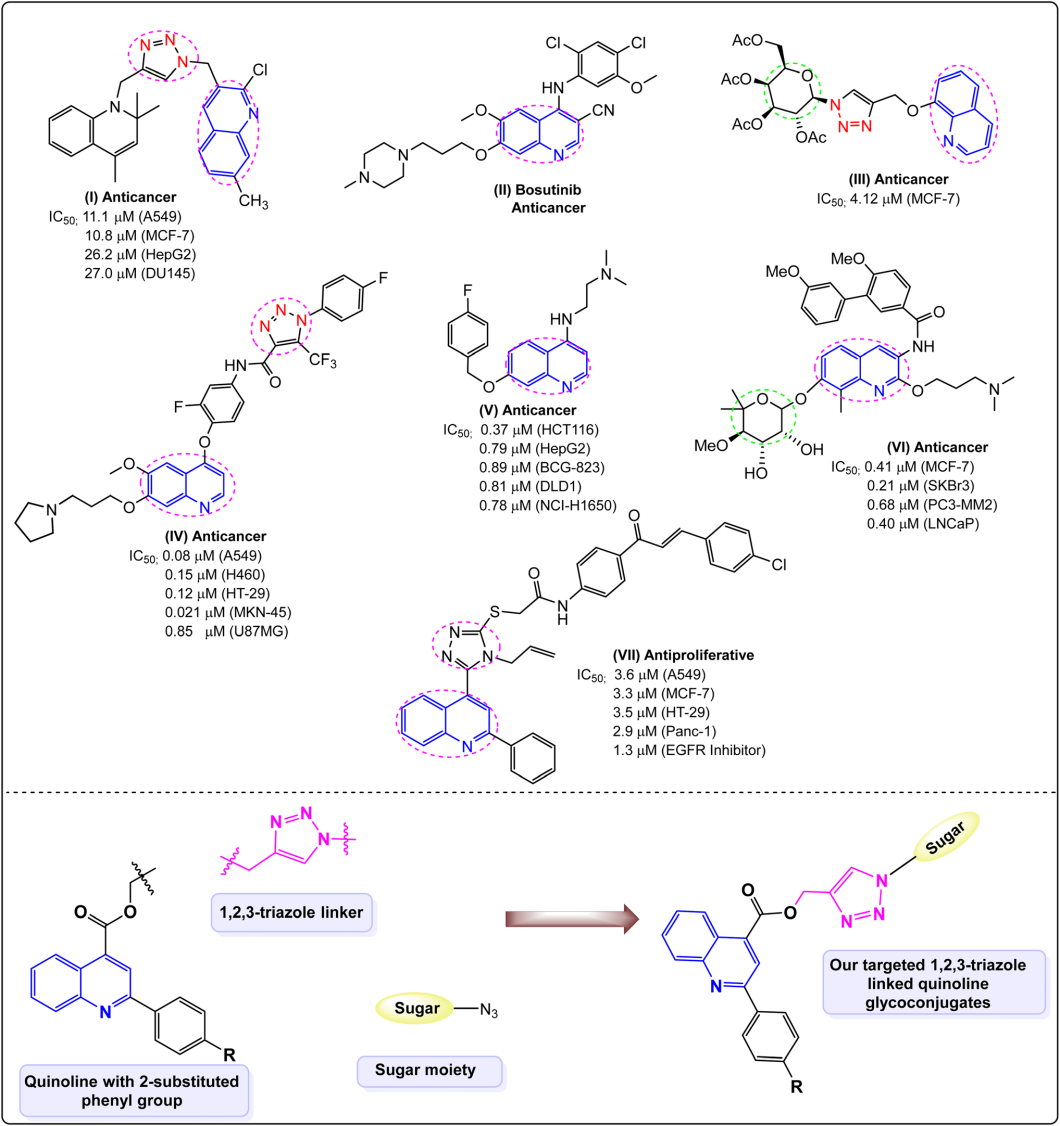
Previously reported quinoline triazole derivatives with potent anticancer activity I–VII and our synthesized 2-phenylquinoline glycoconjugates.

Numerous quinoline- and 1,2,3-triazole-based compounds (I–VII) reported in the literature have exhibited notable anticancer activity, highlighting these motifs as privileged scaffolds in anticancer drug design (Fig. 1). Compound I, a 1,4-disubstituted 1,2,3-triazole derivative featuring a quinolinyl methylene group at the N-1 position and a 1,2-dihydroquinolinylmethylene group at the C-4 position, demonstrated significant cytotoxic activity against lung cancer cells. Additionally, its ability to inhibit phosphodiesterase 4 (PDE4) highlights its potential as a promising anticancer candidate.^14^ The compound II, known as SKI-606 (bosutinib), is a quinoline-based anticancer agent that effectively inhibits Src-mediated signalling pathways, thereby suppressing the migration and invasion of breast cancer cells.^15^ The compound III, a 1,2,3-triazole-linked quinoline glycoconjugate, showed potent anticancer activity against MCF-7 cells. The 1,2,3-triazole moiety enhanced cytotoxicity, and the combination of the 8-hydroxyquinoline core, acetyl-protected sugar, and triazole linker was key to its activity.^16^ The compound IV, a quinoline-based 1,2,3-triazole-4-carboxamide, showed potent antitumor activity, with a 4-fluoro substituent on the phenyl ring and a trifluoromethyl group at C-5 of triazole enhanced c-Met inhibition and cytotoxicity.^17^ In 2018, Li *et al.* reported that compound V, featuring a bulky alkoxy group at the 7-position and amino side chains at the 4-position with an optimal two-CH_2_ unit length, demonstrated strong anticancer activity against colorectal cancer cells by triggering p53/Bax-mediated apoptosis through the activation of p53 transcriptional signaling.^18^ Kusuma *et al.* developed novobiocin analogues by replacing the coumarin framework with a quinoline scaffold to assess their anticancer potential through Hsp90 inhibition. Among these, compound VI displayed notable antiproliferative effects across various cancer cell lines, identifying 2-alkoxyquinoline derivatives as key active structures.^19^ Compound VII, a quinoline–chalcone–triazole hybrid, showed the most potent antiproliferative activity against multiple cancer cell lines, in which the presence of the allyl and triazole moieties played a crucial role in enhancing the biological activity. It also inhibited EGFR and BRAF kinases and induced apoptosis with G2/M phase arrest in Panc-1 cells, confirming its strong anticancer potential.^20^

In light of these reported studies, the present work describes the synthesis and characterization of a series of 2-phenyl derivatives of quinoline-4-carboxy 1,2,3-triazole glycoconjugates 6(a–k). Single-crystal X-ray diffraction analysis was carried out to elucidate the solid-state structures of compounds 6a, 6b, and 6e. The anticancer potential of the compounds was subsequently evaluated *in vitro* against the MCF-7 human breast cancer cell line. Furthermore, comprehensive computational studies were carried out to assess the chemical stability, electronic properties, and binding interactions of the synthesized derivatives with the estrogen receptor alpha (ERα; PDB ID: 3ERT).

## 2. Experimental section

### 2.1. General experimental methods

All experiments were performed using analytical grade reagents and solvents without further purification. Thin-layer chromatography (TLC) was conducted on silica gel 60-F_254_ pre-coated aluminium plates (0.25 mm), and spots were visualized under UV light, by exposure to iodine vapours, or by charring after spraying with 5% H_2_SO_4_ in methanol. Column chromatography was carried out on silica gel (230–400 mesh) using suitable solvent systems. Melting points were determined in open capillaries using a digital melting point apparatus. IR spectra were recorded on a Bruker FTIR 5300 spectrophotometer using KBr pellets (4000–400 cm^−1^). ^1^H and ^13^C NMR spectra were obtained on JEOL (500/125 MHz) and Bruker (600/150 MHz) spectrometers. Chemical shifts (*δ*) are given in ppm relative to TMS, and coupling constants (*J*) in Hz. The following abbreviations are used: s = singlet, d = doublet, t = triplet, dd = double doublet, m = multiplet, br s = broad singlet. HRMS data were recorded on an X500-QTOF mass spectrometer with an electrospray ionization (ESI) source.

### 2.2. General experimental procedure for the synthesis of 3(a–e)

Isatin (1, 10 mmol) was dissolved in a stirred mixture of 33% aq. potassium hydroxide (10 mL) and ethanol (20 mL). To this solution, an appropriate acetophenone derivative (2a–2e, 10 mmol) was added, and the reaction mixture was refluxed for 18 h. The reaction mixture was cooled to room temperature and solvent was evaporated under reduced pressure. The aqueous layer was collected, cooled to 0 °C, and acidified dropwise with concentrated HCl until precipitation occurred (pH ∼2). The resulting solid was filtered, washed with brine and then with cold ethanol, and dried. Recrystallization from ethanol afforded a light brown solid in good yield. Other derivatives (3b–3e) were synthesized using the same procedure, giving yields up to 88%.^21^

#### 2.2.1. 2-Phenylquinoline-4-carboxylic acid (3a)

Yield: 2.10 g (86%); m.p. 196–198 °C; *R*_f_ 0.30 (2 : 8, MeOH : CHCl_3_, v/v); ^1^H NMR (500 MHz, CDCl_3_): *δ*_ppm_ = 8.76 (d, *J* = 8.9 Hz, 1H), 8.38 (s, 1H), 8.11 (d, *J* = 7.9 Hz, 3H), 7.66 (t, *J* = 7.9 Hz, 1H), 7.51 (t, *J* = 7.9 Hz, 1H), 7.44 (t, *J* = 7.4 Hz, 2H), 7.38 (t, *J* = 7.3 Hz, 1H); ^13^C NMR (150 MHz, CDCl_3_): *δ* = 168.27, 156.47, 149.0, 138.66, 136.66, 129.91, 129.55, 129.47, 128.71, 127.32, 127.24, 125.64, 124.09, 120.21; HRMS (ESI): *m*/*z* calculated for C_16_H_12_NO_2_, [M + H]^+^ 250.0790, found 250.0837, *m*/*z* calculated for C_16_H_11_NNaO_2_, [M + Na]^+^ 272.0687, found 272.0683.

### 2.3. General experimental procedure for the synthesis of 4(a–e)

To a solution of 2-phenylquinoline-4-carboxylic acid 3a (1.69 mmol, 1.0 equiv.) in dry dimethylformamide (DMF), and alcoholic sodium carbonate (Na_2_CO_3_, 2.03 mmol, 1.2 equiv.) was added as a base in a round-bottom flask. Subsequently, propargyl bromide (1.69 mmol, 1.0 equiv.) was added dropwise to the reaction mixture, which was then stirred at room temperature for 5 h. The progress of the reaction was monitored by thin-layer chromatography (TLC) using a 40% ethyl acetate and hexane solvent system. Upon completion, the reaction mixture was diluted with ethyl acetate, and the organic layer was sequentially washed with brine and dried over anhydrous sodium sulphate. The solvent was evaporated under reduced pressure to obtain the crude product, which was purified by column chromatography using 20% ethyl acetate in hexane as the eluent. All other derivatives were synthesized using the same standardized procedure, affording yields of up to 86%.

#### 2.3.1. Prop-2-yn-1-yl 2-phenylquinoline-4-carboxylate (4a)

A solution of 2-phenylquinoline-4-carboxylic acid 3a (0.400 g, 1.695 mmol, 1.0 equiv.), propargyl bromide (0.128 mL, 2.03 mmol, 1.2 equiv.), 3 mL alcoholic Na_2_CO_3_ solution (0.234 g, 1.695 mmol, 1.0 equiv.) was prepared in 4 mL dry dimethyl formamide and reacted as described above. After chromatographic purification 4a was obtained. Light brown powder; yield 0.414 g (85%); m.p. 90–92 °C; *R*_f_ 0.30 (2 : 8, EtOAc/hexane v/v); ^1^H NMR (600 MHz, CDCl3): *δ* = 8.77 (d, *J* = 8.5 Hz, 1H), 8.44 (s, 1H), 8.25–8.21 (m, 3H), 7.78 (t, *J* = 7.7 Hz, 1H), 7.64 (t, *J* = 7.7 Hz, 1H), 7.55 (t, *J* = 7.6 Hz, 2H), 7.50 (t, *J* = 7.3 Hz, 1H), 5.08 (d, *J* = 2.5 Hz, 2H), 2.61 (t, *J* = 2.5 Hz, 1H); ^13^C NMR (150 MHz, CDCl3): *δ* = 165.63, 156.85, 149.40, 138.81, 134.83, 130.51, 130.15, 129.93, 129.09, 128.14, 127.62, 125.41, 124.03, 120.70, 77.32, 75.86, 53.25; HRMS (ESI): *m*/*z* calculated for C_19_H_14_NO_2_, [M + H]^+^ 288.0946, found 288.1012.

#### 2.3.2. Prop-2-yn-1-yl 2-(4-chlorophenyl) quinoline-4-carboxylate (4b)

A solution of 2-(4-chlorophenyl) quinoline-4-carboxylic acid 3b (0.350 g, 1.233 mmol, 1.0 equiv.), propargyl bromide (0.093 mL, 1.233 mmol, 1.0 equiv.), 2 mL alcoholic Na_2_CO_3_ solution (0.170 g, 1.233 mmol, 1.0 equiv.), was prepared in 4 mL dry dimethyl formamide and reacted as described above. After chromatographic purification 4b was obtained. Yellowish crystalline powder; yield 0.341 g (86%); m.p. 103–105 °C; *R*_f_ 0.35 (2 : 8, EtOAc/hexane v/v); ^1^H NMR (500 MHz, CDCl_3_): *δ* = 8.77 (d, *J* = 9.3 Hz, 1H), 8.40 (s, 1H), 8.21 (d, *J* = 8.0 Hz, 1H), 8.17 (d, *J* = 9.3 Hz, 2H), 7.79 (t, *J* = 7.3 Hz, 1H), 7.66 (t, *J* = 7.3 Hz, 1H), 7.52 (d, *J* = 8.0 Hz, 2H), 5.08 (s, 2H), 2.61 (s, 1H); ^13^C NMR (125 MHz, CDCl_3_): *δ* = 165.54, 155.55, 149.42, 137.24, 136.26, 135.10, 130.52, 130.35, 129.31, 128.90, 128.37, 125.48, 124.12, 120.23, 77.28, 75.92, 53.32; HRMS (ESI): *m*/*z* calculated for C_19_H_13_ClNO_2_, [M + H]^+^ 322.0557, found 322.0606.

#### 2.3.3. Prop-2-yn-1-yl 2-(4-methoxyphenyl) quinoline-4-carboxylate (4c)

A solution of 2-(4-methoxyphenyl) quinoline-4-carboxylic acid 3c (0.300 g, 1.074 mmol, 1.0 equiv.), propargyl bromide (0.081 mL, 1.047 mmol, 1.0 equiv.), 1.5 mL alcoholic Na_2_CO_3_ solution (0.148 g, 1.047 mmol, 1.0 equiv.), was prepared in 4 mL dry dimethyl formamide and reacted as described above. After chromatographic purification 4c was obtained. Reddish brown powder; yield 0.286 g (84%); m.p. 100–102 °C; *R*_f_ 0.40 (2 : 8, EtOAc/hexane v/v); ^1^H NMR (600 MHz, CDCl_3_): *δ* = 8.73 (d, *J* = 7.0 Hz, 1H), 8.39 (s, 1H), 8.20–8.18 (m, 3H), 7.75 (t, *J* = 6.9 Hz, 1H), 7.60 (t, *J* = 7.0 Hz, 1H), 7.07–7.05 (m, 2H), 5.07 (d, *J* = 2.5 Hz, 2H), 3.89 (s, 3H), 2.60 (t, *J* = 2.5 Hz, 1H); ^13^C NMR (150 MHz, CDCl_3_): *δ* = 165.72, 161.32, 156.38, 149.39, 134.69, 131.37, 130.28, 130.06, 129.00, 127.70, 125.38, 123.69, 120.28, 114.47, 75.82, 55.55, 53.20; HRMS (ESI): *m*/*z* calculated for C_20_H_16_NO_3_, [M + H]^+^ 318.1052, found 318.1108.

#### 2.3.4. Prop-2-yn-1-yl 2-(4-fluorophenyl) quinoline-4-carboxylate (4d)

A solution of 2-(4-fluorophenyl) quinoline-4-carboxylic acid 3d (0.400 g, 1.491 mmol, 1.0 equiv.), propargyl bromide (0.113 mL, 1.491 mmol, 1.0 equiv.), 2 mL alcoholic Na_2_CO_3_ solution (0.206 g, 1.491 mmol, 1.0 equiv.), was prepared in 5 mL dry dimethyl formamide and reacted as described above. After chromatographic purification 4d was obtained. Brownish powder; yield 0.388 g (85%); m.p. 95–97 °C; *R*_f_ 0.35 (2 : 8, EtOAc/hexane v/v); ^1^H NMR (600 MHz, CDCl_3_): *δ* = 8.75 (d, *J* = 7.2 Hz, 1H), 8.38 (d, *J* = 2.6 Hz, 1H), 8.21–8.19 (m, 2H), 7.77 (t, *J* = 6.9 Hz, 1H), 7.63 (t, *J* = 7.0 Hz, 1H), 7.22 (t, *J* = 8.6 Hz, 2H), 7.04 (d, *J* = 8.8 Hz, 1H), 5.07 (d, *J* = 2.6 Hz, 2H), 2.61 (t, *J* = 2.5 Hz, 1H); ^13^C NMR (150 MHz, CDCl_3_): *δ* = 165.52, 155.66, 149.38, 134.94, 130.39, 130.25, 129.54, 129.49, 128.96, 128.17, 125.42, 123.91, 120.26, 116.11, 115.97, 114.97, 77.26, 75.90, 53.18; HRMS (ESI): *m*/*z* calculated for C_19_H_13_FNO_2_, [M + H]^+^ 306.0852, found 306.0921.

#### 2.3.5. Prop-2-yn-1-yl 2-(4-methylphenyl) quinoline-4-carboxylate (4e)

A solution of 2-(4-methylphenyl) quinoline-4-carboxylic acid 3e (0.450 g, 1.709 mmol, 1.0 equiv.), propargyl bromide (0.129 mL, 1.709 mmol, 1.0 equiv.), 2 mL alcoholicNa_2_CO_3_ solution (0.236 g, 1.709 mmol, 1.0 equiv.), was prepared in 5 mL dry dimethyl formamide and reacted as described above. After chromatographic purification 4e was obtained. Brownish powder; yield 0.433 g (86%); m.p. 100–102 °C; *R*_f_ 0.35 (2 : 8, EtOAc/hexane v/v); ^1^H NMR (500 MHz, CDCl_3_): *δ* = 8.75 (d, *J* = 9.3 Hz, 1H), 8.43 (s, 1H), 8.22 (d, *J* = 8.2 Hz, 1H), 8.12 (d, *J* = 8.0 Hz, 2H), 7.77 (t, *J* = 8.0 Hz, 1H), 7.64–7.61 (m, 1H), 7.35 (d, *J* = 6.9 Hz, 2H), 5.08 (d, *J* = 2.7 Hz, 2H), 2.60 (t, *J* = 2.7 Hz, 1H), 2.45 (s, 3H); ^13^C NMR (125 MHz, CDCl_3_): *δ* = 165.74, 156.85, 149.45, 140.15, 136.08, 134.79, 130.47, 130.08, 129.84, 127.93, 127.53, 125.43, 123.95, 120.56, 77.03, 75.82, 53.22, 21.51; HRMS (ESI): *m*/*z* calculated for C_20_H_16_NO_2_, [M + H]^+^ 302.1103, found 302.1162.

### 2.4. General experimental procedure for the synthesis of 1,2,3-triazole linked quinoline glycoconjugates 6(a–k)

A solution of prop-2-yn-1-yl 2-phenylquinoline-4-carboxylate (4a; 1.0 equiv.), 2,3,4,6-tetra-*O*-acetyl-β-d-glucopyranosyl azide and (5a; 1.0 equiv.), was prepared in dichloromethane. To this reaction mixture, *N*,*N*-diisopropylethylamine (DIPEA; 0.5 equiv.) and copper iodide (CuI; 0.3 equiv.) were added with continuous stirring at room temperature. The reaction mixture was stirred at room temperature for 6 h, and the progress was monitored by TLC using 50% ethyl acetate in hexane. Visualization of TLC was performed by charring with 5% concentrated H_2_SO_4_ in methanol. After completion, the solvent was evaporated under reduced pressure, and the crude product was subsequently purified by column chromatography. Other derivatives (6b–6k) were synthesized following the same general procedure.

#### 2.4.1. 1-(2,3,4,6-Tetra-*O*-acetyl-β-d-glucopyranosyl)-1*H*-1,2,3-triazole-4-yl)methyl-2-phenylquinoline-4-carboxylate (6a)

A solution of 4a (0.250 g, 0.870 mmol), 2,3,4,6-tetra-*O*-acetyl-β-d-glucopyranosyl azide (5a; 0.324 g, 0.870 mmol), CuI (0.049 g, 0.261 mmol) and DIPEA (0.075 mL, 0.435 mmol), was prepared in 4 mL dichloromethane and reacted as described above. After chromatographic purification obtained 6a. White crystalline powder; yield 0.454 g (79%); m.p. 165–167 °C; *R*_f_ 0.30 (3 : 7, EtOAc : hexane, v/v); IR (*ν* cm^−1^, KBr) 1460C=N, 1598 C=C, 1745 (–O{CO}), 2923, 3095; ^1^H NMR (500 MHz, CDCl_3_): *δ* 8.73 (d, *J* = 9.3 Hz, 1H), 8.40 (s, 1H), 8.20 (dd, *J* = 13.3, 8.0 Hz, 3H), 8.03 (s, 1H), 7.76 (t, *J* = 7.3 Hz, 1H), 7.63 (t, *J* = 8.0 Hz, 1H), 7.52 (t, *J* = 7.3 Hz, 2H), 7.49–7.45 (m, 1H), 5.91–5.88 (m, 1H), 5.62 (s, 2H), 5.45–5.41 (m, 2H), 5.26–5.21 (m, 1H), 4.30 (dd, *J* = 12.7, 4.7 Hz, 1H), 4.15 (d, *J* = 12.0 Hz, 1H), 4.03–3.99 (m, 1H), 2.06 (s, 3H), 2.05 (s, 3H), 2.02 (s, 3H), 1.82 (s, 3H). ^13^C NMR (125 MHz, CDCl_3_): *δ* = 170.58, 169.99, 169.46, 168.96, 166.29, 156.88, 149.40, 143.31, 138.84, 135.27, 130.49, 130.09, 129.89, 129.04, 128.06, 127.66, 125.48, 124.07, 122.87, 120.69, 86.06, 75.45, 72.63, 70.57, 67.83, 61.62, 58.60, 20.77, 20.63, 20.20; HRMS (ESI): *m*/*z* calculated for C_33_H_33_N_4_O_11_, [M + H]^+^ 661.2068, found 661.2141.

#### 2.4.2. 1-(2,3,4,6-Tetra-*O*-acetyl-β-d-glucopyranosyl)-1*H*-1,2,3-triazole-4-yl)methyl-2-(4-chlorophenyl) quinoline-4-carboxylate (6b)

A solution of prop-2-yn-1-yl 2(4-chlorophenyl)quinoline-4-carboxylate 4b (0.300 g, 0.932 mmol), 5a (0.347 g, 0.932 mmol), CuI (0.053 g, 0.279 mmol) and DIPEA (0.081 mL, 0.466 mmol), was prepared in 4 mL dichloromethane and reacted as described above. After chromatographic purification 6b was obtained. Light yellow powder; yield 0.531 g (82%); m.p. 163–165 °C; *R*_f_ 0.35 (3 : 7, EtOAc : hexane, v/v); IR (*ν* cm^−1^, KBr) 1547C=N, 1596 C=C, 1732 (–O{CO}), 2976, 3117; ^1^H NMR (600 MHz, CDCl_3_): *δ* = 8.73 (d, *J* = 7.6 Hz, 1H), 8.36 (s, 1H), 8.18 (d, *J* = 8.2 Hz, 1H), 8.16–8.14 (m, 2H), 8.03 (s, 1H), 7.76 (ddd, *J* = 8.4, 6.8, 1.4 Hz, 1H), 7.63 (ddd, *J* = 8.3, 6.8, 1.3 Hz, 1H), 7.49–7.47 (m, 2H), 5.91–5.89 (m, 1H), 5.62 (s, 2H), 5.43–5.42 (m, 2H), 5.24 (ddd, *J* = 9.7, 6.4, 2.8 Hz, 1H), 4.30 (dd, *J* = 12.6, 5.0 Hz, 1H), 4.15 (dd, *J* = 12.7, 2.1 Hz, 1H), 4.01 (ddd, *J* = 10.2, 5.0, 2.2 Hz, 1H), 2.05 (s, 6H), 2.02 (s, 3H), 1.83 (s, 3H); ^13^C NMR (150 MHz, CDCl_3_): *δ* = 170.55, 169.98, 169.45, 168.98, 166.11, 155.47, 149.32, 143.22, 137.16, 136.15, 135.38, 130.44, 130.25, 129.22, 128.89, 128.26, 125.50, 124.09, 122.87, 120.19, 86.04, 75.44, 72.59, 70.55, 67.79, 61.59, 58.62, 20.78, 20.65, 20.62, 20.23; HRMS (ESI): *m*/*z* calculated for C_33_H_32_ClN_4_O_11_, [M + H]^+^ 695.1678, found 695.1763.

#### 2.4.3. 1-(2,3,4,6-Tetra-*O*-acetyl-β-d-glucopyranosyl)-1*H*-1,2,3-triazole-4-yl)methyl-2-(4-methoxyphenyl) quinoline-4-carboxylate (6c)

A solution of prop-2-yn-1-yl 2(4-methoxyphenyl)quinoline-4-carboxylate 4c (0.350 g, 1.10 mmol), 5a (0.411 g, 1.10 mmol), CuI (0.063 g, 0.330 mmol) and DIPEA (0.096 mL, 0.551 mmol), was prepared in 4 mL dichloromethane and reacted as described above. After chromatographic purification 6c was obtained. White powder; yield 0.624 g (82%); m.p. 170–172 °C; *R*_f_ 0.40 (3 : 7, EtOAc : hexane, v/v); IR (*ν* cm^−1^, KBr) 1589C=N, 1604 C=C, 1754 (–O{CO}), 2922, 3138; ^1^H NMR (500 MHz, CDCl_3_): *δ* = 8.69 (d, *J* = 8.5 Hz, 1H), 8.36 (s, 1H), 8.16 (d, *J* = 8.9 Hz, 3H), 8.03 (s, 1H), 7.73 (t, *J* = 6.9 Hz, 1H), 7.58 (t, *J* = 7.0 Hz, 1H), 7.03 (d, *J* = 8.8 Hz, 2H), 5.93–5.87 (m, 1H), 5.62 (s, 2H), 5.47–5.39 (m, 2H), 5.24 (t, *J* = 9.7 Hz, 1H), 4.30 (dd, *J* = 12.6, 5.0 Hz, 1H), 4.15 (d, *J* = 12.6 Hz, 1H), 4.01 (td, *J* = 4.9, 2.7 Hz, 1H), 3.87 (s, 3H), 2.05 (d, *J* = 5.0 Hz, 6H), 2.02 (s, 3H), 1.82 (s, 3H); ^13^C NMR (125 MHz, CDCl_3_): *δ* = 170.57, 169.99, 169.44, 168.94, 166.34, 161.28, 156.36, 149.35, 143.30, 135.06, 131.36, 130.23, 129.98, 129.01, 127.61, 125.42, 123.70, 122.88, 120.25, 114.41, 86.01, 75.40, 72.61, 70.51, 67.78, 61.59, 58.54, 55.52, 20.77, 20.63, 20.61, 20.21; HRMS (ESI): *m*/*z* calculated for C_34_H_35_N_4_O_12_, [M + H]^+^ 691.2173, found 691.2230.

#### 2.4.4. 1-(2,3,4,6-Tetra-*O*-acetyl-β-d-glucopyranosyl)-1*H*-1,2,3-triazole-4-yl)methyl-2-(4-fluorophenyl) quinoline-4-carboxylate (6d)

A solution of prop-2-yn-1-yl 2(4-fluorophenyl)quinoline-4-carboxylate (4d; 0.300 g, 0.982 mmol), 5a (0.366 g, 0.982 mmol), CuI (0.056 g, 0.294 mmol) and DIPEA (0.085 mL, 0.491 mmol), was prepared in 4 mL dichloromethane and reacted as described above. After chromatographic purification 6d obtained. White cream powder; yield 0.546 g (82%); m.p. 161–163 °C; *R*_f_ 0.35 (3 : 7, EtOAc : hexane, v/v); IR (*ν* cm^−1^, KBr) 1588C=N, 1600C=C, 1755 (–O{CO}), 2924, 3098; ^1^H NMR (600 MHz, CDCl_3_): *δ* 8.72 (dd, *J* = 16.8, 8.8 Hz, 1H), 8.37 (s, 1H), 8.22–8.15 (m, 3H), 8.02 (s, 1H), 7.75 (dt, *J* = 15.5, 7.9 Hz, 1H), 7.61 (dt, *J* = 21.5, 7.9 Hz, 1H), 7.22–7.18 (m, 1H), 7.03 (d, *J* = 8.2 Hz, 1H), 5.62 (s, 2H), 5.43 (d, *J* = 8.0 Hz, 2H), 5.24 (t, *J* = 6.8 Hz, 1H), 4.31 (dd, *J* = 12.6, 4.8 Hz, 1H), 4.14 (dd, *J* = 18.1, 10.0 Hz, 2H), 4.01 (dd, *J* = 10.3, 5.0 Hz, 1H). ^13^C NMR (150 MHz, CDCl_3_): *δ* = 171.82, 170.08, 169.45, 168.98, 166.22, 160.74, 155.75, 149.76, 143.28, 135.10, 130.43, 130.29, 129.99, 129.57, 129.04, 128.13, 127.61, 125.52, 122.85, 120.28, 116.12, 115.0, 86.10, 75.50, 72.66, 70.60, 67.86, 61.64, 58.64, 20.78, 20.63, 20.22; HRMS (ESI): *m*/*z* calculated for C_33_H_32_FN_4_O_11_, [M + H]^+^ 679.1973, found 679.2066.

#### 2.4.5. 1-(2,3,4,6-Tetra-*O*-acetyl-β-d-glucopyranosyl)-1*H*-1,2,3-triazole-4-yl)methyl-2-(4-methylphenyl)quinoline-4-carboxylate (6e)

A solution of prop-2-yn-1-yl 2(4-methylphenyl)quinoline-4-carboxylate (4e; 0.250 g, 0.830 mmol), 5a (0.309 g, 0.830 mmol), CuI (0.047 g, 0.249 mmol) and DIPEA (0.072 mL, 0.415 mmol), was prepared in 4 mL dichloromethane and reacted as described above. After chromatographic purification 6e was obtained. White powder; yield 0.453 g (81%); m.p. 166–168 °C; *R*_f_ 0.30 (3 : 7, EtOAc : hexane, v/v); IR (*ν* cm^−1^, KBr) 1493C=N, 1596C=C, 1749 (–O{CO}), 2922, 3064; ^1^H NMR (600 MHz, CDCl_3_): *δ* = 8.71 (d, *J* = 9.8 Hz, 1H), 8.39 (s, 1H), 8.19 (d, *J* = 7.6 Hz, 1H), 8.10 (d, *J* = 8.2 Hz, 2H), 8.03 (s, 1H), 7.75 (t, *J* = 6.9 Hz, 1H), 7.60 (t, *J* = 7.7 Hz, 1H), 7.32 (d, *J* = 8.1 Hz, 2H), 5.90–5.88 (m, 1H), 5.62 (s, 2H), 5.44–5.42 (m, 2H), 5.26–5.22 (m, 1H), 4.30 (dd, *J* = 12.6, 5.0 Hz, 1H), 4.15 (dd, *J* = 12.6, 2.2 Hz, 1H), 4.02–3.99 (m, 1H), 2.43 (s, 3H), 2.06 (d, *J* = 4.5 Hz, 6H), 2.02 (s, 3H), 1.83 (s, 3H); ^13^C NMR (150 MHz, CDCl_3_): *δ* = 170.58, 169.99, 169.44, 168.94, 166.34, 156.79, 149.37, 143.32, 140.08, 136.01, 135.11, 130.40, 130.00, 129.77, 127.83, 127.50, 125.44, 123.93, 122.87, 120.51, 86.03, 75.42, 72.62, 70.52, 67.79, 61.59, 58.57, 21.49, 20.79, 20.65, 20.62, 20.22; HRMS (ESI): *m*/*z* calculated for C_33_H_35_N_4_O_11_, [M + H]^+^ 675.2224, found 675.2290.

#### 2.4.6. 1-(2,3,4,6-Tetra-*O*-acetyl-β-d-galactopyranosyl)-1*H*-1,2,3-triazole-4-yl)methyl-2-phenylquinoline-4-carboxylate (6f)

A solution of 4a (0.250 g, 0.870 mmol), 2,3,4,6-Tetra-*O*-acetyl-*β*-d-glactopyranosyl azide (5b; 0.324 g, 0.870 mmol), CuI (0.049 g, 0.261 mmol) and DIPEA (0.075 mL, 0.435 mmol), was prepared in 4 mL dichloromethane and reacted as described above. After chromatographic purification 6f was obtained. White powder; yield 0.459 g (80%); m.p. 160–162 °C; *R*_f_ 0.30 (3 : 7, EtOA : hexane, v/v); IR (*ν* cm^−1^, KBr) 1495C=N, 1549 C=C, 1755 (–O{CO}), 2925, 3472; ^1^H NMR (500 MHz, CDCl_3_): *δ* = 8.73 (d, *J* = 9.3 Hz, 1H), 8.41 (s, 1H), 8.21 (dd, *J* = 12.0, 8.0 Hz, 3H), 8.08 (s, 1H), 7.77 (t, *J* = 8.0 Hz, 1H), 7.63 (t, *J* = 7.3 Hz, 1H), 7.54–7.48 (m, 2H), 7.46 (d, *J* = 8.0 Hz, 1H), 5.86 (d, *J* = 9.3 Hz, 1H), 5.63 (s, 2H), 5.55–5.53 (m, 2H), 5.26 (dd, *J* = 10.0, 4.7 Hz, 1H), 4.24–4.20 (m, 1H), 4.18 (q, *J* = 4.0 Hz, 1H), 4.14 (dd, *J* = 12.0, 6.7 Hz, 1H), 2.20 (s, 3H), 2.01 (d, *J* = 10.7 Hz, 6H), 1.85 (s, 3H); ^13^C NMR (125 MHz, CDCl_3_): *δ* = 170.42, 170.08, 169.88, 169.12, 166.34, 156.86, 149.41, 143.21, 138.83, 135.33, 130.52, 130.10, 129.90, 129.05, 128.04, 127.64, 125.46, 124.05, 123.00, 120.66, 86.60, 74.37, 70.79, 68.16, 66.96, 61.29, 58.59, 20.77, 20.72, 20.58, 20.29; HRMS (ESI): *m*/*z* calculated for C_33_H_32_N_4_O_11_, [M + H]^+^ 661.2068, found 661.2141.

#### 2.4.7. 1-(2,3,4,6-Tetra-*O*-acetyl-β-d-galactopyranosyl)-1*H*-1,2,3-triazole-4-yl)methyl-2-(4-chlorophenyl) quinoline-4-carboxylate (6g)

A solution of 4b (0.300 g, 0.932 mmol), 5b (0.347 g, 0.932 mmol), CuI (0.053 g, 0.279 mmol) and DIPEA (0.081 mL, 0.466 mmol), was prepared in 4 mL dichloromethane and reacted as described above. After chromatographic purification 6g was obtained. Yellowish powder; yield 0.537 g (83%); m.p. 161–163 °C; *R*_f_ 0.35 (3 : 7, EtOAc : hexane, v/v); IR (*ν* cm^−1^, KBr) 1575 C=N, 1627 C=C, 1755 (–O{CO}), 2926, 3328; ^1^H NMR (600 MHz, DMSO-*d*_6_): *δ* = 8.61 (d, *J* = 8.4 Hz, 1H), 8.36 (s, 1H), 8.29 (s, 1H), 8.19 (d, *J* = 8.2 Hz, 2H), 8.10 (d, *J* = 8.4 Hz, 1H), 7.94 (s, 1H), 7.76 (t, *J* = 7.6 Hz, 1H), 7.61 (t, *J* = 7.6 Hz, 1H), 7.47 (d, *J* = 8.2 Hz, 2H), 6.21 (d, *J* = 9.1 Hz, 1H), 5.54 (dd, *J* = 21.4, 8.1 Hz, 2H), 5.43 (s, 2H), 4.53 (t, *J* = 6.4 Hz, 1H), 4.11 (dd, *J* = 11.6, 5.6 Hz, 1H), 4.02 (dd, *J* = 11.4, 6.9 Hz, 1H), 3.36 (ddd, *J* = 10.5, 7.3, 3.8 Hz, 1H), 2.13 (s, 3H), 1.93 (d, *J* = 11.8 Hz, 6H), 1.76 (s, 3H); ^13^C NMR (150 MHz*,* DMSO-*d*_6_): *δ* = 168.34, 167.87, 167.06, 164.05, 155.80, 153.32, 147.31, 140.87, 135.30, 134.27, 134.09, 128.78, 128.62, 127.50, 127.37, 126.64, 123.83, 122.44, 122.18, 118.08, 83.56, 71.80, 69.00, 66.72, 65.83, 59.92, 57.13, 19.13, 19.07, 19.00, 18.69; HRMS (ESI): *m/z* calculated for C_33_H_32_ClN_4_O_11_, [M + H]^+^ 695.1678, found 695.1759.

#### 2.4.8. 1-(2,3,4,6-Tetra-*O*-acetyl-β-d-galactopyranosyl)-1*H*-1,2,3-triazole-4-yl)methyl-2-(4-methoxyphenyl) quinoline-4-carboxylate (6h)

A solution of 4c (0.350 g, 1.10 mmol), 5b (0.411 g, 1.10 mmol), CuI (0.063 g, 0.330 mmol) and DIPEA (0.096 mL, 0.551 mmol), was prepared in 4 mL dichloromethane and reacted as described above. After chromatographic purification 6h was obtained. Cream colour powder; yield 0.608 g (80%); m.p. 164–166 °C; *R*_f_ 0.35 (3 : 7, EtOAc : hexane, v/v); IR (*ν* cm^−1^, KBr) 1591 C=N, 1605C=C, 1747 (–O{CO}), 2966, 3134; ^1^H NMR (500 MHz, CDCl_3_): *δ* = 8.70 (d, *J* = 9.4 Hz, 1H), 8.36 (s, 1H), 8.17 (d, *J* = 8.9 Hz, 3H), 8.08 (s, 1H), 7.75–7.73 (m, 1H), 7.60–7.57 (m, 1H), 7.03 (d, *J* = 8.9 Hz, 2H), 5.86 (d, *J* = 9.4 Hz, 1H), 5.63 (s, 2H), 5.55–5.53 (m, 2H), 5.25 (dd, *J* = 10.4, 3.4 Hz, 1H), 4.24–4.12 (m, 3H), 3.88 (s, 3H), 2.20 (s, 3H), 2.02 (s, 3H), 2.00 (s, 3H), 1.85 (s, 3H); ^13^C NMR (125 MHz, CDCl_3_): *δ* = 170.42, 170.08, 169.89, 169.12, 166.41, 161.29, 156.38, 149.37, 143.22, 135.15, 131.37, 130.26, 130.00, 129.01, 127.61, 125.41, 123.70, 123.02, 120.24, 114.42, 86.56, 74.33, 70.77, 68.11, 66.93, 61.29, 58.54, 20.78, 20.74, 20.60, 20.32; HRMS (ESI): *m*/*z* calculated for C_34_H_35_N_4_O_12_, [M + H]^+^ 691.2173, found 691.2224.

#### 2.4.9. 1-(2,3,4,6-Tetra-*O*-acetyl-β-d-galactopyranosyl)-1*H*-1,2,3-triazole-4-yl)methyl-2-(4-fluorophenyl) quinoline-4-carboxylate (6i)

A solution of 4d (0.300 g, 982 mmol), 5b (0.366 g, 0.982 mmol), CuI (0.056 g, 0.294 mmol) and DIPEA (0.085 mL, 0.491 mmol), was prepared in 4 mL dichloromethane and reacted as described above. After chromatographic purification 6i was obtained. White powder; yield 0.540 g (81%); m.p. 163–165 °C; *R*_f_ 0.30 (3 : 7, EtOAc : hexane, v/v); IR (*ν* cm^−1^, KBr) 1503 C=N, 1592 C=C, 1750 (–O{CO}), 2926, 3489; ^1^H NMR (600 MHz, CDCl_3_): *δ* = 8.72 (d, *J* = 8.0 Hz, 1H), 8.36 (s, 1H), 8.21–8.18 (m, 2H), 8.08 (s, 1H), 7.76 (t, *J* = 8.0 Hz, 1H), 7.64–7.61 (m, 1H), 7.20 (t, *J* = 8.7 Hz, 2H), 7.02 (d, *J* = 9.3 Hz, 1H), 5.87 (d, *J* = 9.3 Hz, 1H), 5.63 (s, 2H), 5.55–5.53 (m, 2H), 5.26 (dd, *J* = 10.7, 4.0 Hz, 1H), 4.22–4.13 (m, 3H), 2.20 (s, 3H), 2.02 (s, 3H), 2.00 (s, 3H), 1.85 (s, 3H); ^13^C NMR (150 MHz, CDCl_3_): *δ* = 170.39, 170.04, 169.86, 169.14, 166.23, 155.69, 149.36, 143.17, 135.45, 130.41, 130.20, 129.6, 129.53, 129.0, 128.08, 125.48, 123.96, 122.97, 120.23, 116.08, 115.91, 114.96, 86.6, 74.39, 70.78, 68.19, 66.96, 61.30, 58.61, 20.74, 20.70, 20.57, 20.29; HRMS (ESI): *m*/*z* calculated for C_33_H_32_FN_4_O_11_, [M + H]^+^ 679.1973, found 679.2020.

#### 2.4.10. 1-(2,3,4,6-Tetra-*O*-acetyl-β-d-galactopyranosyl)-1*H*-1,2,3-triazole-4-yl)methyl-2-(4-methylphenyl) quinoline-4-carboxylate (6j)

A solution of 4e (0.250 g, 0.830 mmol), 5b (0.309 g, 0.830 mmol), CuI (0.047 g, 0.249 mmol) and DIPEA (0.072 mL, 0.415 mmol), was prepared in 4 mL dichloromethane and reacted as described above. After chromatographic purification 6j was obtained. White powder; yield 0.447 g (80%); m.p. 161–163 °C; *R*_f_ 0.30 (3 : 7, EtOAc : hexane, v/v); IR (*ν* cm^−1^, KBr) 1548 C=N, 1593 C=C, 1755 (–O{CO}), 2923, 3492; ^1^H NMR (600 MHz, CDCl_3_): *δ* = 8.71 (d, *J* = 10.1 Hz, 1H), 8.39 (s, 1H), 8.20 (d, *J* = 9.8 Hz, 1H), 8.10 (d, *J* = 8.2 Hz, 2H), 8.07 (s, 1H), 7.75 (t, *J* = 8.4 Hz, 1H), 7.61 (t, *J* = 8.4 Hz, 1H), 7.32 (d, *J* = 8.0 Hz, 2H), 5.86 (d, *J* = 9.4 Hz, 1H), 5.63 (s, 2H), 5.55–5.53 (m, 2H), 5.26 (dd, *J* = 10.4, 3.4 Hz, 1H), 4.23–4.12 (m, 3H), 2.43 (s, 3H), 2.20 (s, 3H), 2.02 (s, 3H), 2.00 (s, 3H), 1.85 (s, 3H); ^13^C NMR (150 MHz, CDCl_3_): *δ* = 170.42, 170.09, 169.89, 169.12, 166.41, 156.78, 149.36, 143.21, 140.08, 135.99, 135.19, 130.37, 130.01, 129.77, 127.81, 127.49, 125.41, 123.91, 123.03, 120.49, 86.56, 74.33, 70.77, 68.11, 66.93, 61.29, 58.55, 21.49, 20.78, 20.74, 20.60, 20.31; HRMS (ESI): *m*/*z* calculated for C_33_H_35_N_4_O_11_, [M + H]^+^ 675.2224, found 675.2323.

#### 2.4.11. 1-(5-Deoxy-1,2-*O*-isopropylidene-α-d-xylofuranose-5-yl)-1*H*-1,2,3-triazol-4-yl)methyl-2-phenylquinoline-4-carboxylate (6k)

A solution of 4a (0.200 g, 0.69 mmol), 5-azido-5-deoxy-1,2-*O*-isopropylidene-α-d-xylofuranose^22^ (5c; 0.150 g, 0.69 mmol), CuI (0.040 g, 0.21 mmol) and DIPEA (60 µL, 0.34 mmol), was prepared in 4 mL dichloromethane and reacted as described above. After chromatographic purification 6k was obtained. Light creamy powder; yield 0.284 g (81%); m.p. 155–158 °C; *R*_f_ 0.35 (3 : 7, EtOAc : hexane, v/v); ^1^H NMR (500 MHz, CDCl_3_) *δ* 8.74 (d, *J* = 9.3 Hz, 1H), 8.40 (s, 1H), 8.21 (d, *J* = 8.0 Hz, 1H), 8.16 (d, *J* = 6.7 Hz, 2H), 7.90 (s, 1H), 7.76 (t, *J* = 7.3 Hz, 1H), 7.62 (t, *J* = 8.0 Hz, 1H), 7.53 (t, *J* = 7.3 Hz, 2H), 7.47 (t, *J* = 6.7 Hz, 1H), 5.96 (d, *J* = 4.0 Hz, 1H), 5.66–5.56 (m, 2H), 4.79 (dd, *J* = 13.3, 6.7 Hz, 1H), 4.64–4.52 (m, 2H), 4.47 (t, *J* = 6.7 Hz, 1H), 4.23 (s, 1H), 3.49 (d, *J* = 5.3 Hz, 1H), 1.41 (s, 3H), 1.27 (s, 3H)·^13^C NMR (125 MHz, CDCl_3_) *δ* 166.29, 156.93, 149.34, 142.55, 138.78, 135.19, 130.42, 130.15, 129.93, 129.09, 128.11, 127.67, 125.75, 125.43, 124.07, 120.75, 112.24, 105.26, 85.39, 79.20, 74.76, 58.69, 48.86, 26.89, 26.24. HRMS (ESI): *m*/*z* calculated for C_22_H_26_N_4_O_6_, [M + H]^+^ 503.1852, found 503.1884.

### 2.5. Cell culture

The MCF-7 human breast cancer cell line was obtained from the National Centre for Cell Science (NCCS), Pune, India. The cells were maintained in Dulbecco's Modified Eagle's Medium (DMEM) enriched with 10% fetal bovine serum (FBS) and 100 U mL^−1^ penicillin–streptomycin, under standard culture conditions of 37 °C in a humidified atmosphere containing 5% CO_2_. Stock solutions of the synthesized compounds 6(a–j) were initially prepared in dimethyl sulfoxide (DMSO) and subsequently diluted with complete medium to achieve the required test concentrations. The final concentration of DMSO in all experimental treatments was kept below 0.1% (v/v) to avoid solvent-related cytotoxic effects.

### 2.6. Cell viability assay

The cytotoxic activity of the synthesized compounds 6(a–j) was evaluated against MCF-7 human breast cancer cells using the MTT colorimetric assay. In brief, cells were seeded into 96-well culture plates and incubated for 48 hours under standard growth conditions (37 °C, 5% CO_2_, and a humidified atmosphere) to achieve approximately 70% confluence. After cell attachment, the medium was replaced with fresh DMEM containing various concentrations of the test compounds (50–200 µg mL^−1^), followed by an additional 48-hours incubation. Post-treatment, the medium was replaced with 200 µL of serum-free DMEM containing 20 µL of MTT solution (5 mg mL^−1^) and incubated for 4 hours to facilitate formazan crystal formation. The resulting supernatant was carefully removed, and the formazan precipitate was dissolved in 100 µL of DMSO. The absorbance of each well was recorded at 590 nm using a microplate reader, and the cell viability was calculated relative to the untreated control group.% Of cell survival = (mean O. D. of the drug treated well/mean O.D. of the control well) ×100.

### 2.7. Molecular docking study

The crystal structure of the human oestrogen receptor alpha (ERα) complexed with the inhibitor 4-hydroxy tamoxifen (PDB ID: 3ERT) was obtained from RCSB Protein Data Bank (https://www.rcsb.org). Molecular docking simulations were performed using AutoDock vina 4.2 to examine the interaction between the ligand and the receptor.^23,24^ Using the Gaussian09W software package, geometry optimisations were carried out at the B3LYP/6-31G(d,p) level of theory to determine the structures of compounds 6(a–j). The ligand–protein interactions were analysed and visualised in both 2D and 3D formats using BIOVIA Discovery Studio Visualiser.

### 2.8. Crystallography

Single clear white needle-shaped crystals of 6a, 6b and 6e were obtained by recrystallisation from ethyl acetate and chloroform layering. A suitable crystal 0.03 × 0.02 × 0.01 mm was selected and mounted on a suitable support on an XtaLAB Synergy, Dualflex, HyPix3000 diffractometer. The crystal was kept at a steady *T* = 293 K during data collection. The structure was solved with the ShelXT 2018/2.^25^ Structure solution program using the Intrinsic phasing solution method and by using Olex2 as the graphical interface.^26^ The model was refined with version 2019/2 of ShelXL 2019/2 using Least Squares minimisation.^27^

### 2.9. DFT studies

All DFT calculations in the present study were carried out using the Gaussian 09W software package.^28^ The visualization and interpretation of the optimized geometries and electronic properties were performed using GaussView 5.0.^29^ Initially, all molecular geometries were optimized in the gas phase without imposing any symmetry constraints. The calculations were conducted at the density functional theory (DFT) level employing the B3LYP functional. The B3LYP method combines Becke's three-parameter hybrid exchange functional (B3) with the Lee–Yang–Parr (LYP) correlation functional, providing a reliable balance between computational efficiency and accuracy.^30^ This functional effectively incorporates both local and non-local electron correlation effects, making it widely suitable for studying molecular geometries, electronic structures, and reactivity parameters.

## 3. Result and discussion

### 3.1. Synthesis

Quinoline-4-carboxylic acids and their derivatives are important building blocks for various pharmaceuticals and other biologically active compounds. The Pfitzinger reaction is a versatile and efficient method for the synthesis of quinoline-4-carboxylic acids, but it can be challenging to achieve high yields and good purity.^21^ In this study, the Pfitzinger reaction was successfully employed to synthesize a series of 2-phenylquinoline-4-carboxylic acids 3(a–e) from isatin and substituted acetophenones under mild conditions, affording excellent yields (85–88%). The reactions were carried out in ethanol using an aqueous KOH under reflux conditions, and the products were isolated by simple filtration followed by recrystallization.

The structure of compound 3a was confirmed by spectroscopic analysis. Its IR spectrum showed characteristic absorption bands at 3400–3300 cm^−1^ corresponding to O–H stretching and at 1670–1700 cm^−1^ attributed to C=O stretching of the carboxylic acid group. In the ^13^C NMR spectrum, a signal at *δ* 168.27 ppm was assigned to the carboxylic acid carbon. Further confirmation was done by the HRMS analysis, which showed a molecular ion peak at *m*/*z* 250.0837 [M + H]^+^, consistent with the molecular formula C_16_H_12_NO_2_. Similar spectroscopic features confirmed structure of remaining compounds 3(b–e).

**Scheme 1:**
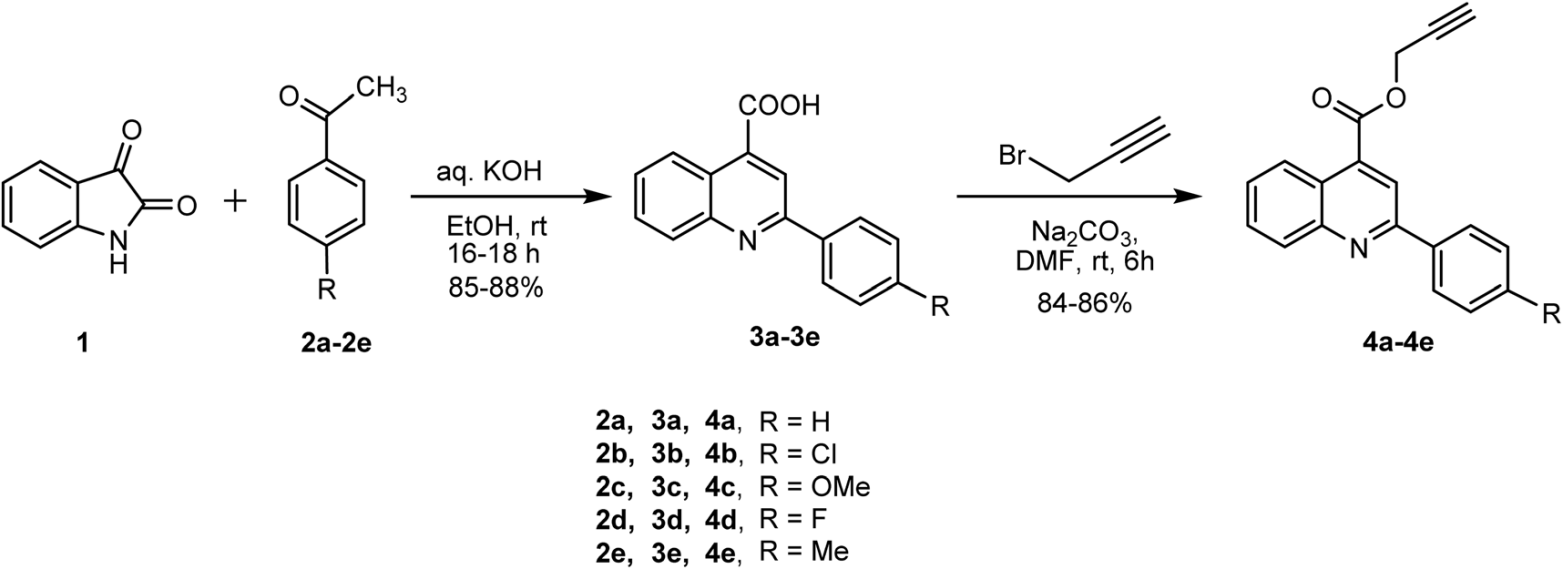
Synthesis of prop-2-yn-1-yl 2-phenylquinoline-4-carboxylate derivatives (4a–4e).

Subsequently, compounds 3(a–e) were subjected to propargylation using propargyl bromide in the presence of potassium carbonate in DMF at room temperature. This reaction furnished the corresponding prop-2-yn-1-yl 2-phenylquinoline-4-carboxylate derivatives 4(a–e) in good yields (84–86%) (Scheme 1). The successful formation of the propargylated products was confirmed by NMR and HRMS analyses. The ^1^H NMR spectra of compound 4a displayed peak at *δ* 2.60 ppm corresponding to the acetylenic proton (≡C*H*) and a doublet at *δ* 5.07 ppm assigned to methylene protons (–OC*H*_2_–). In the ^13^C NMR spectrum, signals observed at approximately *δ* 75 ppm corresponded to acetylenic carbon (C≡CH), and the signal at *δ* 53.25 ppm was resonated for the methylene carbon (–O*C*H_2_–). HRMS spectrum further supported the structure of compound 4a, showing a molecular ion peak at *m*/*z* 288.1012 [M + H]^+^, in agreement with the molecular formula C_19_H_14_NO_2_.

Next, glycopyranosyl azides 5a and 5b were synthesized starting from d-glucose and d-galactose, respectively. The sugars were first peracetylated using acetic anhydride in pyridine to afford the corresponding per-*O*-acetylated glycopyranosides, which were then converted to 2,3,4,6-tetra-*O*-acetyl-α-d-glycopyranosyl bromides using 33% HBr in glacial acetic acid at 0 °C to room temperature in dry dichloromethane. Subsequently, these intermediates were reacted with sodium azide in DMSO at room temperature, furnishing 2,3,4,6-tetra-*O*-acetyl-β-d-glycopyranosyl azides 5a and 5b in excellent yields. 5-Azido-5-deoxy-1,2-*O*-isopropylidene-α-d-xylofuranose (5c) was synthesized from the d-xylose using a series of reactions according to reported method.^22^

**Scheme 2:**
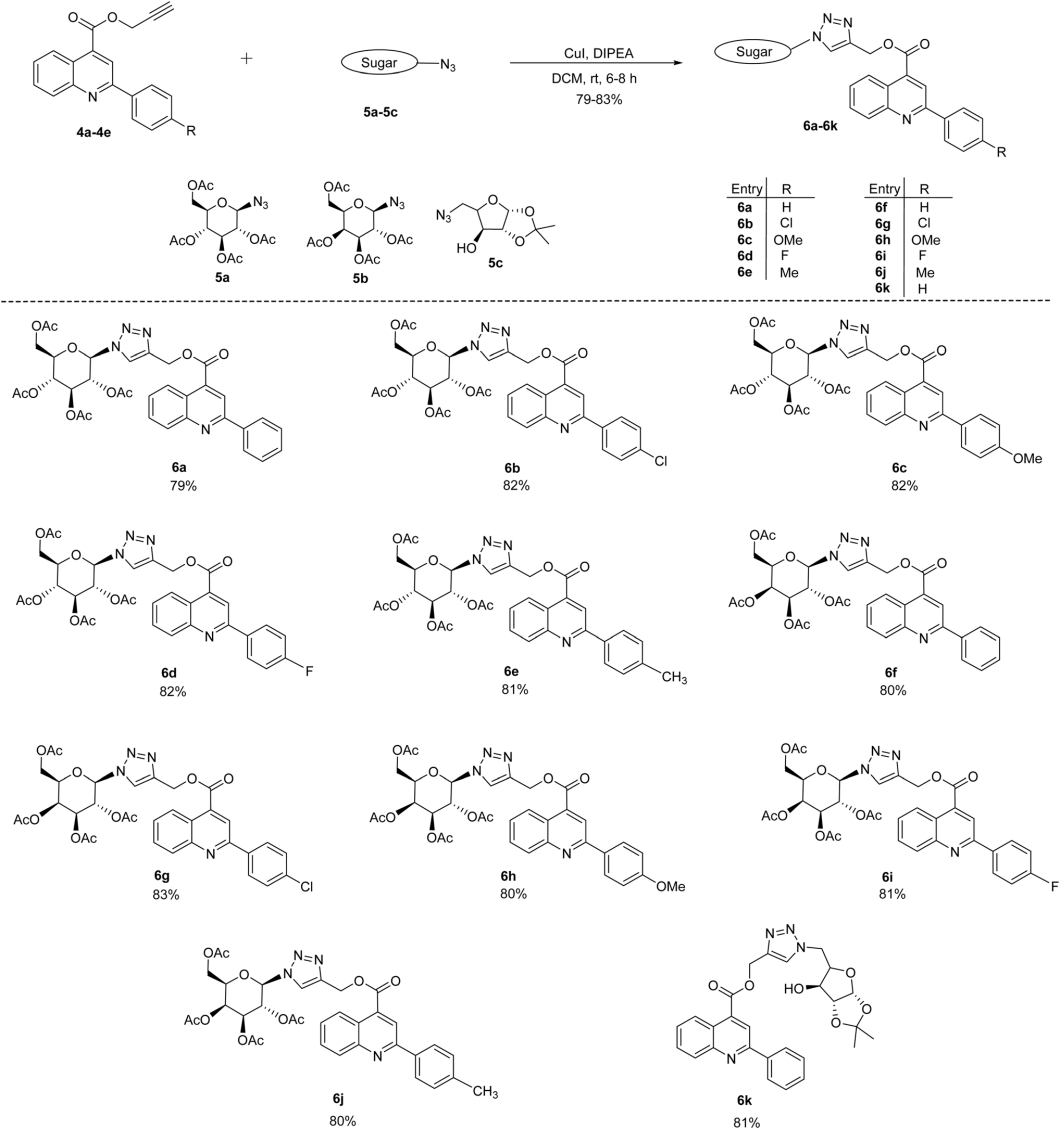
Synthesis of 1,2,3- triazole linked quinoline glycoconjugates 6(a–k).

A Cu-(I) catalyzed azide–alkyne cycloaddition reaction was performed between quinoline-derived alkynes (4a–4e) and glycopyranosyl azides (5a, 5b and 5c) in dichloromethane using catalytic amount of CuI and *N*,*N*-diisopropylethylamine at room temperature (Scheme 2). This reaction afforded the desired 1,4-disubstituted 1,2,3-triazole-linked 2-phenylquinoline-4-carboxylate derivatives 6(a–k) in 79–83% yield. The structures of compounds 6(a–k) were confirmed by IR, ^1^H & ^13^C NMR spectroscopy and HRMS analyses. The ^1^H NMR spectrum of compound 6a showed a characteristic singlet corresponding to triazolyl proton at *δ* 8.03 ppm, whereas two methylene (–C*H*_2_−) protons attached to the triazole ring resonated as a singlet at *δ* 5.62 ppm. Seven protons corresponding to sugar moiety resonated between *δ* 5.90–4.00 ppm. In the ^13^C NMR of 6a, the methylene carbon (–*C*H_2_−) was observed at *δ* 58.60 ppm, while sugar carbons appeared between *δ* 86.06–61.62 ppm. The HRMS spectrum of 6a exhibited a molecular ion peak at *m*/*z* 661.2141 [M + H]^+^, corresponding to the molecular formula C_33_H_33_N_4_O_11_. Further, the structures of compound 6a, 6b, and 6e was confirmed by single-crystal X-ray diffraction (SC-XRD) analyses.

### 3.2. Anticancer activity

The library of 2-phenylquinoline–triazole-bridged *N*-glycoconjugates 6(a–j) was evaluated for their anticancer potency against MCF-7 cancer cell lines. MTT assay was employed to determine the growth inhibitory effects and reduction in cell viability of MCF-7 cells. Initially, cells were treated with different concentrations of the compounds 6(a–j) (50–200 µg mL^−1^) for 48 h, The IC_50_ value were determined based on the percentage of cell viability, as summarized in (Table 1). Compounds 6c, 6d, 6f, and 6i exhibited weak cytotoxicity, however rest other derivatives were found inactive. The structure activity relationship (SAR) of glycoconjugates 6(a–j) against MCF-7 cells was preliminary analysed with respect to the nature of sugar scaffold and the electronic effect of aryl substituent attached to the quinoline ring. Overall, galactosyl derivatives exhibited enhanced antiproliferative activity corresponding to their glucosyl analogues. Among all the compounds, the compound 6f was the most potent, exhibiting an IC_50_ value of 108.59 µM. Notably, the electron donating substituent such as methoxy group provided moderate activity only in glucose derivatives (6c; IC_50_ = 131.25 µM) but loses potency in galactose derivatives (6h; IC_50_ = >200 µM). In contrast, weak donors like methyl were unfavourable (6e and 6j). Among the electron-withdrawing substituents, fluorine-containing analogues displayed better cytotoxic activity (6d; IC_50_ = 135.95 µM and 6i; IC_50_ = 151.36 µM) than their chloro-substituted analogues (6b and 6g). This indicates that substituents exhibiting high electronegativity with minimal steric hindrance are more favourable for activity. The SAR suggests that optimization of the sugar configuration together with electronic effects, steric balance at the 2-aryl quinoline position, is crucial for enhancing anticancer activity.

**Table 1:** Cell viability and IC_50_ value of compounds 6(a–j) against MCF-7 cell line

Compound no.	Cell viability % ± SD			IC_50_ (µM)
	Concentration (µg mL^−1^)			
	50	100	200	
6a	70.74 ± 2.10	66.61 ± 2.19	50.18 ± 0.85	>200
6b	99.46 ± 0.70	81.93 ± 2.12	79.79 ± 1.41	>200
6c	50.46 ± 0.32	54.30 ± 0.88	43.47 ± 3.40	131.25
6d	63.57 ± 0.93	50.96 ± 1.23	48.56 ± 2.67	135.95
6e	57.41 ± 0.70	57.83 ± 0.70	50.49 ± 0.21	>200
6f	75.15 ± 12.39	50.28 ± 7.10	27.98 ± 1.23	108.59
6g	79.76 ± 0.70	64.61 ± 1.41	50.90 ± 0.70	>200
6h	52.60 ± 2.59	50.40 ± 11.20	58.59 ± 3.80	>200
6i	55.92 ± 0.70	51.10 ± 1.22	22.68 ± 1.52	151.36
6j	72.37 ± 8.59	54.95 ± 3.68	49.95 ± 1.22	198.61
Tamoxifen	—	—	—	22

### 3.3. Crystal structure description

**Fig. 2:**
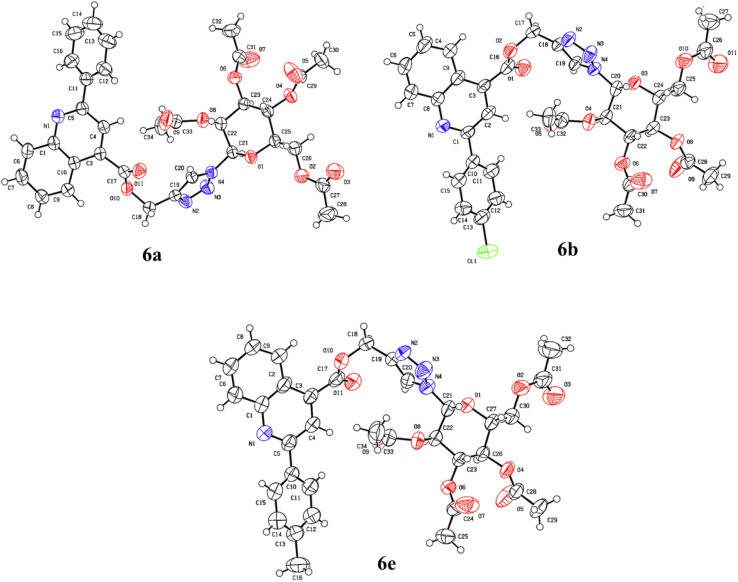
ORTEP plots showing thermal ellipsoids at 50% probability level.

**Fig. 3:**
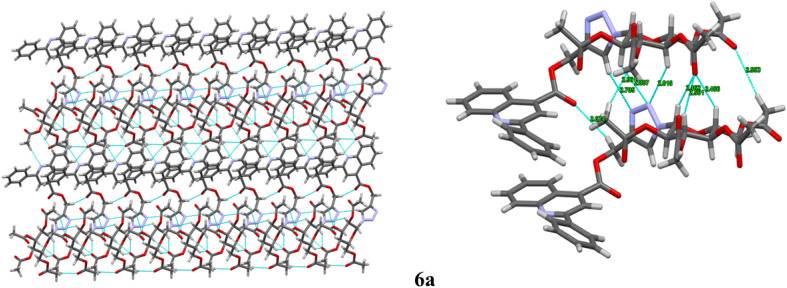
Intermolecular interaction along with hydrogen bonding between C–H, N–H⋯N, C–H⋯N, and C–H⋯O (sp^2^, sp^3^) along *b* axis of molecule 6a.

**Fig. 4:**
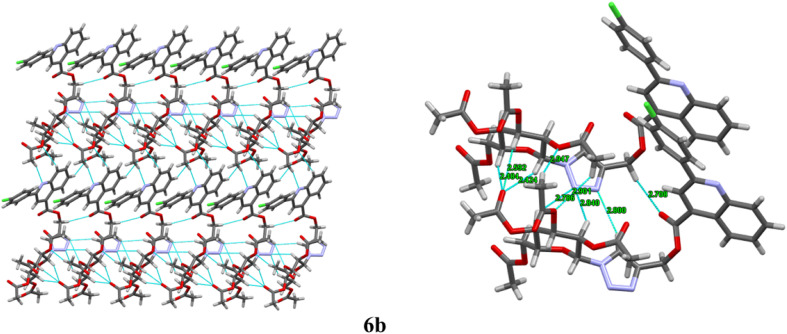
Intermolecular interaction along with hydrogen bonding between (C–H, N–H⋯N, C–H⋯N, and C–H⋯O)(sp^2^, sp^3^) along *b* axis of 6b.

**Fig. 5:**
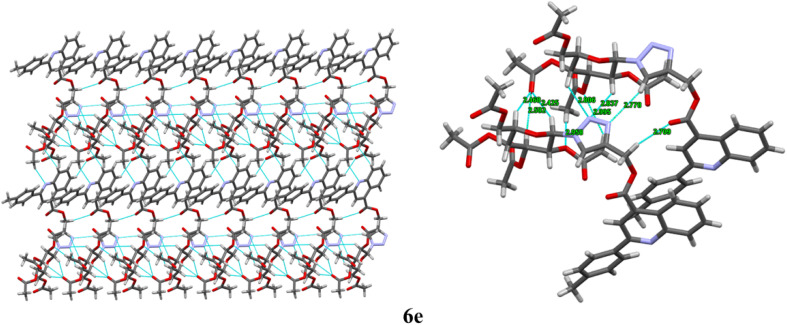
Intermolecular interaction along with hydrogen bonding between (C–H, N–H⋯N, C–H⋯N, and C–H⋯O) (sp^2^, sp^3^) along *b* axis of 6e.

The compounds 6a, 6b and 6e were crystallized from ethyl acetate and hexane. The diffraction data were obtained to solve their three-dimensional structures. The ortep plot of their thermal probability ellipsoids at 50% probability were given in (Fig. 2). The details of crystal structure solution and refinement are given in (Table 2). In compounds 6a, 6b and 6e the quinoline and triazole ring exhibit a non-coplanar orientation. Additionally, the β-anomeric configuration of the sugar ring was confirmed through analysis of the torsional angles at the anomeric centre.^31^ For the compound 6a, the torsional angles C23–C22–C21–N4 and C25–O1–C21–N4 were found to be −176.7° and 167.1°, respectively. In compound 6b, the corresponding torsional angles C22–C21–C20–N4 and C24–O3–C20–N4 were −177.4° and 165.5°, respectively. Similarly, compound 6e exhibited torsional angles of −177.5° for C23–C22–C21–N4 and 167.0° for C27–O1–C21–N4. The values of these dihedral angles, being close to 180°, clearly indicate a β-anomeric orientation of the sugar ring in all three compounds 6a, 6b, and 6e. Furthermore, the presence of sp^3^/sp^2^ C–H⋯N/O and N–H⋯N interaction along with hydrogen bonding is illustrated in (Fig. 3–5). The details of other crucial non covalent interaction that contributed significantly to the crystal packing stabilization of the compounds 6a, 6b, and 6e are summarized in the form of a table (ESI, Table S1).

**Table 2:** Crystal data and structure refinement details

Compound	6a	6b	6e
Empirical formula	C_33_H_32_N_4_O_11_	C_33_H_31_ClN_4_O_11_	C_34_H_34_N_4_O_11_
Formula weight	660.62	695.07	674.65
Temperature	293 K	293 K	298 K
Crystal system	Triclinic	Triclinic	Triclinic
Space group	*P*1	*P*1	*P*1
Unit cell dimensions	*a* = 5.7948 (10) Å	*a* = 5.8670 (2)	*a* = 5.8582 (3)
	*b* = 11.9278 (2) Å	*b* = 12.0574 (4)	*b* = 12.1432 (5)
	*c* = 12.1042 (2) Å	*c* = 12.1900 (4)	*c* = 12.2428 (4)
	*α* = 82.636 (2)° *β* = 87.276 (10)°	*α* = 81.389 (3)	*α* = 81.207 (3)
	*γ* = 81.538 (2)°	*β* = 80.238 (3)°	*β* = 80.608 (3)°
		*γ* = 86.153 (3)	*γ* = 86.419 (4)°
Volume	818.14 (2) Å^3^	839.50 (5) Å^3^	848.93 (6) Å^3^
*Z*	1	1	1
Density (calculated)	1.341 g cm^−3^	1.375 g cm^−3^	1.320 g cm^−3^
Absorption coefficient	0.858 mm^−1^	1.580 mm^−1^	0.838 mm^−1^
*F*(0 0 0)	346.0	362.0	354.0
Theta range for data collection	7.366°–135.926°	7.422°–136.502°	7.372°–144.198°
Index ranges	−6 ≤ *h* ≤ 6	−7 ≤ *h* ≤ 7	−7 ≤ *h* ≤ 7
	−14 ≤ *k* ≤ 14	−11 ≤ *k* ≤ 14	−14 ≤ *k* ≤ 14
	−13 ≤ *l* ≤ 14	−14 ≤ *l* ≤ 14	−15 ≤ *l* ≤ 15
Reflections collected	11 155	7523	8284
Independent reflections	4237 [*R*(int) = 0.0202]	3851 [*R*(int) = 0.0758]	4008 [*R*(int) = 0.039]
Absorption correction	‘Multi-scanʼ	‘Multi-scanʼ	‘Multi-scanʼ
Refinement method	Full-matrix least-squares on *F*^2^	Full-matrix least-squares on *F*^2^	Full-matrix least-squares on *F*^2^
Data/restraints/parameters	4237/3/437	3851/3/446	4008/3/448
Goodness-of-fit on *F*^2^	1.058	1.196	1.185
Final *R* indices	*R*_1_ = 0.0332, w*R*_2_ = 0.0823	*R*_1_ = 0.0905, w*R*_2_ = 0.2669	*R*_1_ = 0.0908, w*R*_2_ = 0.2972
*R* Indices (all data)	*R*_1_ = 0.0342 w*R*_2_ = 0.0834	*R*_1_ = 0.1060 w*R*_2_ = 0.2917	*R*_1_ = 0.1094 w*R*_2_ = 0.3171
Largest diff. Peak and hole	0.12/−0.17*e* Å^−3^	0.48/−0.48*e* Å^−3^	0.41/−0.36*e* Å^−3^
CCDC number	2457702	2424982	2445773

### 3.4. Molecular docking analysis

Molecular docking analyses were performed for the most potent compounds 6c, 6d, and 6f to explore their binding interactions and potential inhibitory mechanisms against the ERα enzyme (PDB ID: 3ERT). Normally, ERα activity is precisely controlled; however, its dysregulation in various cancer types contributes to unchecked cellular proliferation, genomic instability, and tumour development. Tamoxifen acts as a selective ERα modulator that binds to the receptor's ligand-binding domain, blocking estrogen-mediated transcriptional activation and thereby suppressing cancer cell growth. Consequently, the crystal structure of the human estrogen receptor alpha (ERα) ligand-binding domain complexed with 4-hydroxytamoxifen was utilized for the docking studies. The 2-phenylquinoline triazole glycoconjugates 6c, 6d, and 6f displayed strong binding affinity within the ERα active site, with docking energies of −9.1, −9.5 and 8.9 kcal mol^−1^, respectively (ESI, Table S2). To confirm the reliability of the docking procedure, the native co-crystallized ligand, 4-hydroxytamoxifen, was re-docked into the ERα active site, resulting a docking score of −9.7 kcal mol^−1^ and an RMSD value of 1.514 Å. The close alignment of the re-docked conformation with the experimentally determined binding poses further validated the accuracy and robustness of the docking protocol (Fig. S62).

**Fig. 6:**
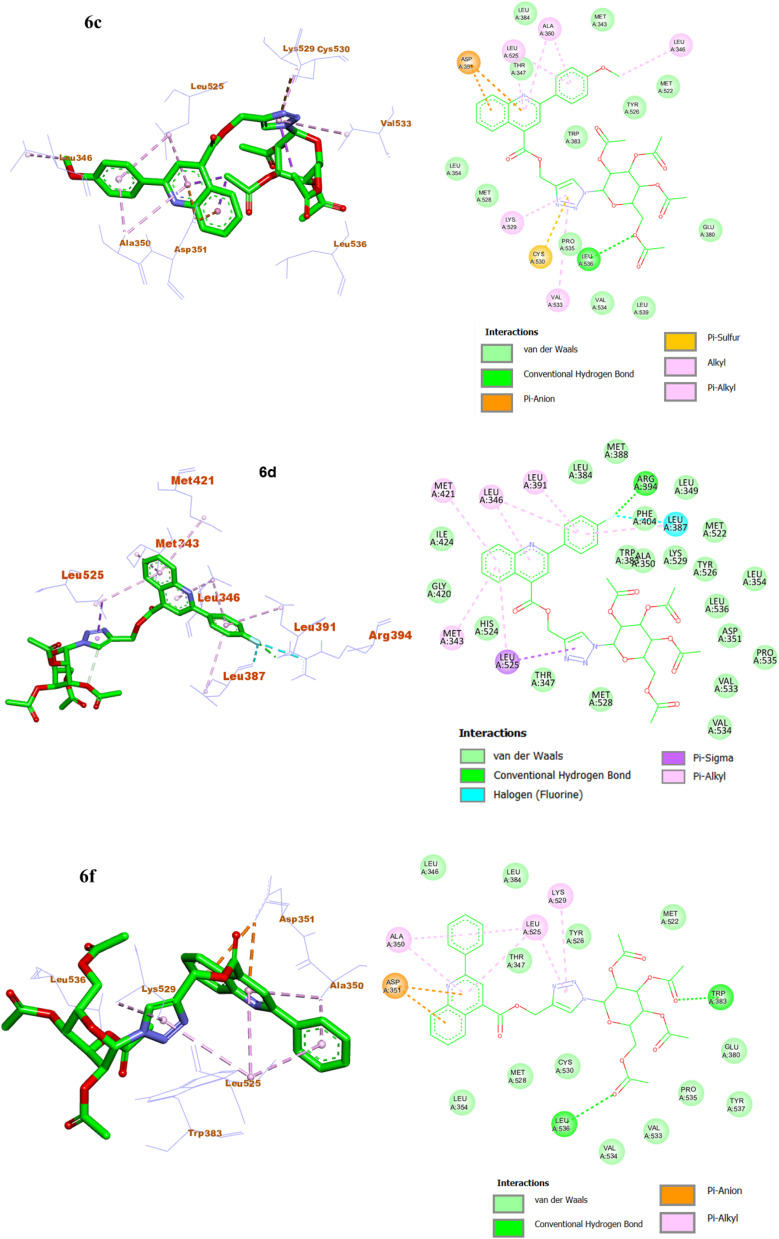
The 3D and 2D bindings mode of compound 6f, 6c, and 6d within the active site of ERα.

Compound 6c displayed a strong affinity and was stabilized within the ERα active site by a hydrogen bond between the acetoxy oxygen of the 6-acetyl group on the sugar moiety and Leu536, along with a π–sulfur interaction between the triazole nitrogen and Cys530. Additional stabilization arose from π-alkyl and alkyl interactions involving the methoxyphenyl, quinoline and triazole rings with residues Ala350, Leu525, and Val533, as well as an interaction between the methoxy substituent and Leu346. A π–anion interaction between Asp351 and the quinoline ring as well as multiple van der Waals contacts with surrounding residues collectively contributed to the overall stability of the 6c–ERα complex (Fig. 6).

The compound 6d formed stable complex with ERα *via* a hydrogen bond interaction between the fluorine atom and Arg394, along with a halogen interaction with the Leu387 residue. Quinoline nucleus showed π–alkyl interactions with Met421 and Met343, whereas the phenyl substituent of quinoline scaffold interacted with Leu391. Moreover, Leu525 formed π–sigma contacts with both the quinoline and triazole cores, and Leu346 involved π–alkyl interactions with the quinoline and fluorophenyl rings. Additionally, van der Waals contacts with neighbouring residues further enhanced the stability of compound 6d with receptor (Fig. 6).

Similarly, compound 6f established favourable binding interactions with receptor Erα through two hydrogen bonds between the acetoxy oxygens of the 6-acetyl and 3-acetyl groups on the sugar moiety and the amino acid residues Leu536 and Trp383, respectively. The binding was further stabilized by π–alkyl interactions involving the quinoline and triazole moieties with Ala350, Leu525, and Lys529 residues. Furthermore, a π–anion interaction between Asp351 and the quinoline framework along with several van der Waals interactions contributed a stable 6f–ERα association (Fig. 6).

Despite the favourable molecular docking scores of compounds 6c, 6d, and 6f against ERα, their *in vitro* antiproliferative activity against the MCF-7 cell line was poor. This discrepancy may be attributed to inherent limitations of molecular docking, which does not fully account for receptor dynamics, solvation, solubility, cellular uptake, or metabolic stability, and often shows limited correlation with experimental potency. Accordingly, these compounds may be considered preliminary leads, warranting further structure-guided optimization to improve biological activity.^32^

### 3.5. Hirshfeld analysis

**Fig. 7:**
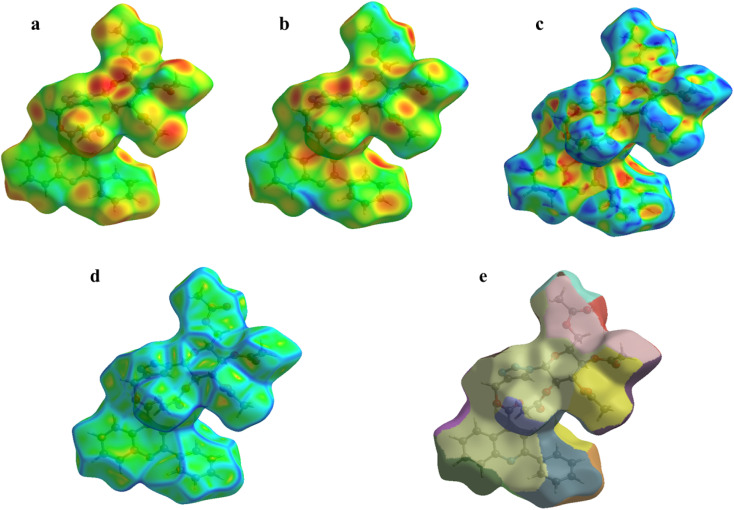
(a) di (b) de (c) shape index (d) curvedness (e) fragment Patch along the *b*-axis of the compound 6a.

**Fig. 8:**
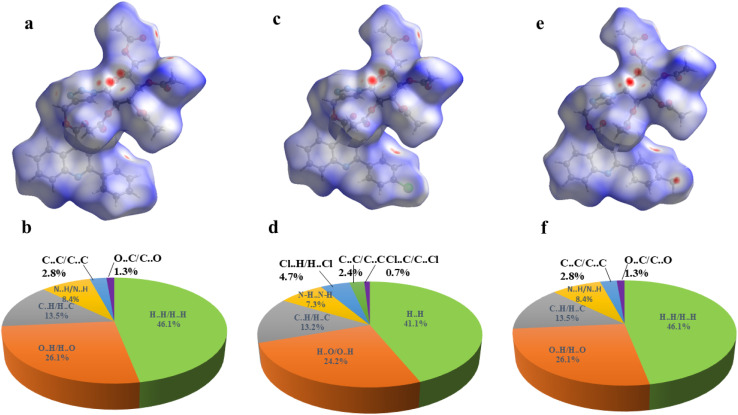
*d*_norm_ surfaces and 2D fingerprint plots for 6a (a and b), 6b (c and d), and 6e (e and f), Pie chart representing the percentage contributions of different 2d Finger interactions in the Hirshfeld surface.

The crystallographic information files (CIFs) of compounds 6a, 6b, and 6e were employed as an input for Hirshfeld surfaces (HS) using Crystal Explorer 17.5 in the default Tonto mode^33^ (ESI, Fig. S63 and S64). The Hirshfeld surfaces were mapped over *d*_norm_, where the color scale represents intermolecular contact distances: red regions indicate close contacts, white regions denote contacts near van der Waals separations, and blue regions correspond to longer intermolecular distances. The surfaces were rendered semi-transparent to facilitate visualization of molecular fragments within the crystal environment. Representative *d*_norm_ surfaces for compound 6a are illustrated in Fig. 7 (ESI, Fig. S65).^29^ The *d*_norm_ maps of 6a, 6b, and 6e reveal distinct red patches, signifying strong intermolecular interactions, mainly of the types O⋯H/H⋯O and N⋯H/H⋯N, which play a crucial role in stabilizing the crystal packing. To quantitatively evaluate these interactions, two-dimensional (2D) fingerprint plots were generated from the corresponding three-dimensional Hirshfeld surfaces. The relative contributions of various intermolecular contacts were calculated and are summarized in Fig. 8, while the percentage contributions of reciprocal contacts are illustrated using pie charts (ESI, Fig. S66 and S67).

### 3.6. DFT study

#### 3.6.1. Geometrical optimization

**Fig. 9:**
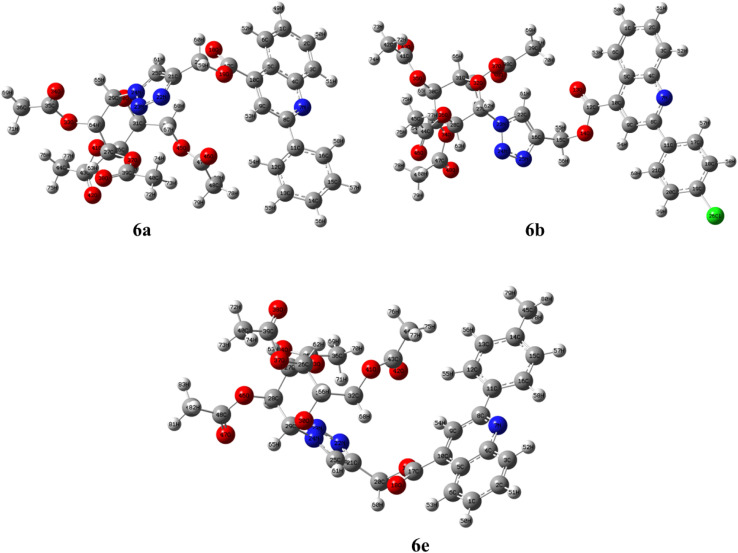
Ground state optimized geometry (in the gas phase) of quinoline −1,2,3-triazole based glycohybrids 6a, 6b, and 6e (along with the atom number and symbol notation).

The three-dimensional geometries of compounds 6a, 6b, and 6e were obtained using DFT calculations in the gas phase at 298.15 K and 1 atm, employing the B3LYP/6–31 G(d,p) basis set. Compounds 6a and 6b consist of 80 atoms each, while compound 6e contains 83 atoms; the optimized structures, along with atomic labeling, are depicted in Fig. 9. To assess the reliability of the computational approach, the geometrical parameters obtained from DFT calculations were compared with the experimental X-ray crystallographic (XRD) data for compounds 6a, 6b, and 6e. The results showed that the bond lengths and bond angles of the compounds closely align with the crystal structure data, *i.e.*, 6a, 6b, and 6e, supporting the successful synthesis of the compounds.

In addition, thermodynamic parameters were calculated at the same level of theory (B3LYP/6-31G(d,p)) under gas-phase conditions at 298.15 K and 1 atm, and the results are summarized in ESI, Table S3. The computed quantities include zero-point vibrational energy (ZPVE), thermal corrections to energy, enthalpy, and Gibbs free energy, as well as the total electronic energy and the sums of electronic and thermal energies, enthalpies, and free energies. Other relevant thermodynamic parameters, such as total entropy and heat capacity at constant volume, were also determined. The calculated entropy values, which provide insight into molecular flexibility and disorder relevant to binding behaviour, were found to be 275.04 cal mol^−1^ K^−1^ for compound 6a, 286.332 cal mol^−1^ K^−1^ for 6b, and 285.82 cal mol^−1^ K^−1^ for 6e.

#### 3.6.2. Atomic charge analysis

**Fig. 10:**
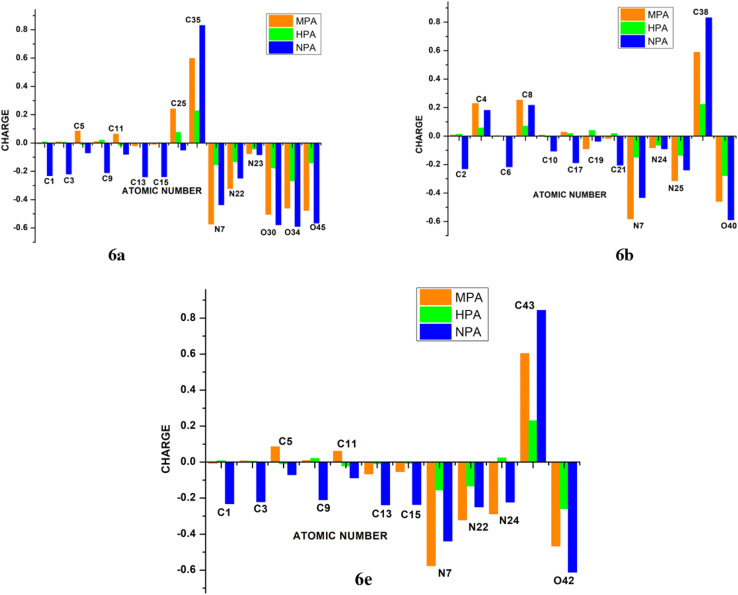
Atomic charge presentation at the selected atoms in 6a, 6b, and 6e based on MPA, HPA, and NPA charge schemes.

Effective atomic charges for compounds 6a, 6b, and 6e were evaluated using Mulliken (MPA), Hirshfeld (HPA), and Natural Population Analysis (NPA) at the B3LYP/6-31G(d,p) level in the gas phase (Fig. 10). Although atomic charges are not directly observable, they provide valuable insight into molecular polarity, electronic distribution, and reactivity. While MPA is computationally efficient, it is highly basis-set dependent, whereas HPA and NPA yield more consistent charge distributions, with NPA typically producing larger charge magnitudes. Accordingly, NPA results were primarily considered for discussion.^34^ According to the NPA results, the C35 atom exhibited the highest positive charge (0.830 a.u.) in compound 6a, while in compound 6b the C38 atom showed the maximum positive charge (0.831 a.u.). For compound 6e, the most positively charged atom was C43, with a value of 0.603 a.u. In all three compounds, the atoms carrying the highest positive charges correspond to the carbonyl carbon atoms of the acetyl sugar moieties. These high positive charges are attributed to the influence of the nearby electronegative oxygen atoms of the acetyl groups, specifically O34 and O33 in 6a; O40 and O37 in 6b; and O42 and O41 in 6e. Several atoms in each compound displayed negative charges. In compound 6a, the negatively charged atoms included N7, N22, N23, C1, C3, C9, C13, and C15. In compound 6b, negatively charged atoms were identified as C2, C6, C10, C17, C19, C21, N7, N24, and N25. For compound 6e, the atoms with negative charges were C1, C3, C9, C13, C15, O42, N7, N22, and N24. Among all atoms, the three oxygen atoms O30, O34, and O45 in compound 6a, O40 in 6b, and O42 in 6e exhibited notably high negative charges in their neutral states. These oxygen atoms were identified as the most basic sites in their respective molecules.

#### 3.6.3. Molecular electrostatic potential

**Fig. 11:**
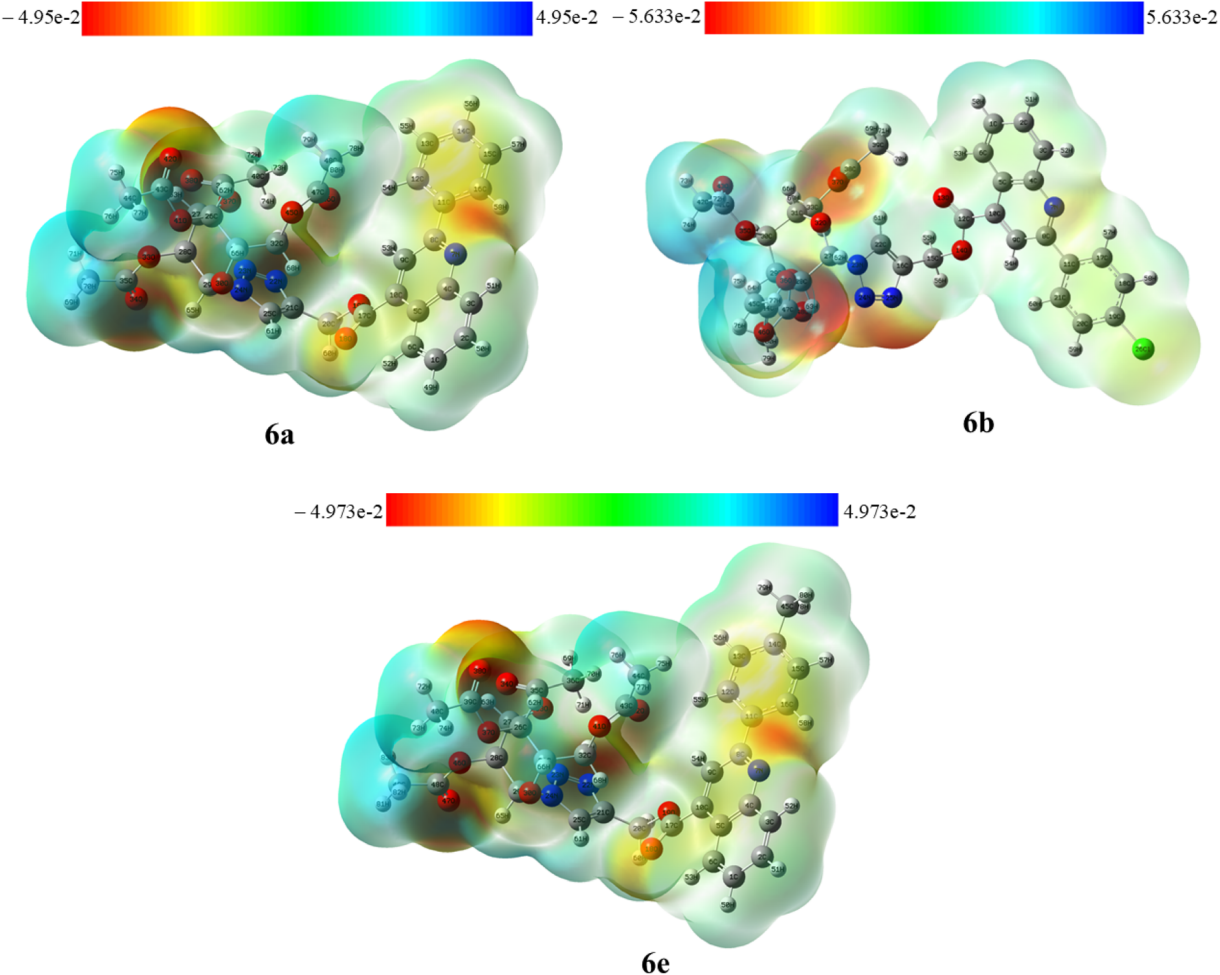
Molecular electrostatic potential (MEP) of the 1,2,3-triazole linked quinoline glycoconjugates 6a, 6b and 6e in the gas phase.

The Molecular Electrostatic Potential (MEP) represents the spatial distribution of electrostatic potential produced by the combined influence of the atomic nuclei and the surrounding electron cloud. It helps visualize how charge is arranged across a molecule and identifies electron-rich (negative, red), electron-poor (positive, blue), and neutral (green) regions. In the colour mapping sequence red → orange → yellow → green → blue, the potential increases correspondingly. MEP mapping serves as an essential computational approach for assessing reactivity trends, molecular recognition patterns, and drug–target interactions.^35^ The MEP surfaces of compounds 6a, 6b, and 6e were generated in the gas phase using B3LYP/6-31G(d,p) optimized geometries (Fig. 11). The potential ranges were −4.95 × 10^−2^ to 4.95 × 10^−2^ a.u. for 6a, −5.633 × 10^−2^ to 5.633 × 10^−2^ a.u. for 6b, and −4.973 × 10^−2^ to 4.973 × 10^−2^ a.u. for 6e. In compounds 6a and 6e, the carbonyl oxygen of the acetylated sugar, oxygens (O18 and O19) near the coumarin ring, and nitrogens of the triazole and quinoline rings showed high electron density areas favourable for electrophilic attack. In 6b, similar red regions were observed near the acetyl carbonyl oxygen and the triazole-quinoline nitrogen's, reflecting comparable charge localization. Conversely, the methyl groups attached to the acetylated sugar in all three molecules were visualized in blue, denoting electron-deficient canters susceptible to nucleophilic attack.

#### 3.6.4. Global reactivity descriptors

**Fig. 12:**
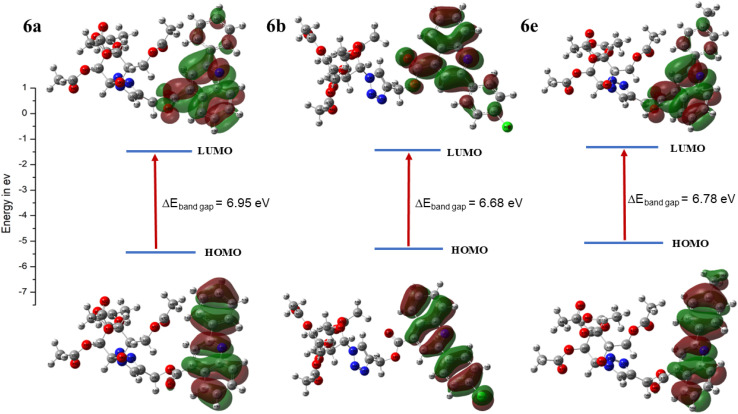
Frontier molecular orbitals contributing in electronic absorption along with band gap (Δ*E*) of compounds 6a, 6b and 6e calculated at B3LYP/6-31G (d,p) level of theory.

Global reactivity parameters derived from conceptual Density Functional Theory (DFT) were evaluated for the ground-state optimized geometries of compounds 6a, 6b, and 6e in the gas phase, and the corresponding results are summarized in Fig. 12, Table 3. In molecular orbital theory, the Highest Occupied Molecular Orbital (HOMO) represents the electron-donating ability of a molecule and is linked to the ionization potential, whereas the Lowest Unoccupied Molecular Orbital (LUMO) acts its electron-accepting capacity and correlates with the electron affinity.^36^ The HOMO–LUMO energy difference (Δ*E*) serves as an indicator of electronic stability and reactivity; a larger gap denotes greater molecular stability and lower reactivity, while a smaller gap suggests enhanced chemical responsiveness.^37^ These electronic indices are widely employed to describe drug–receptor interaction sites and binding behaviour. According to frontier molecular orbital (FMO) theory, the biological response of a compound is significantly influenced by its HOMO–LUMO energy gap. In the present study, compounds 6a, 6b, and 6e displayed nearly identical and relatively large HOMO–LUMO energy gaps (∼7 eV), indicating low molecular reactivity. Notably, all three compounds exhibited poor cytotoxic activity, with IC_50_ values greater than 200 µM. This reduced biological performance can be attributed to the large energy gap, which likely limits efficient electronic interactions with biological targets.^38^

**Table 3:** Global reactivity descriptors for compound 6a, 6b and 6e

Compounds	Dipole moment (debye)^a^	I.P. (eV)^b^	E.A. (eV)^c^	HOMO (eV)	LUMO (eV)	Δ*E* (eV)	*µ* (eV)^d^	*χ* (eV)^e^	*η* (eV)^f^	*S* (1/eV)^g^	*ω* (eV)^h^
6a	5.10	7.51	0.57	−7.51	−0.57	6.95	−4.04	4.05	3.47	0.28	2.36
6b	8.49	7.60	0.91	−7.60	−0.91	6.68	−4.25	4.25	3.34	0.29	2.71
6e	4.71	7.34	0.56	−7.34	−0.56	6.78	−3.95	3.95	3.40	0.30	2.30

a Dipole moment for the sysytem containing *N* number of electrons.

b Ionization potential (I.P.) = *E*_N−1_ − *E*_N_.

c Electron affinity (E.A.) = *E*_N_ − *E*_N+1_.

d Electronic chemical potential (*µ*)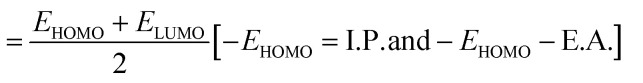.

e Electronegativity (*χ*)
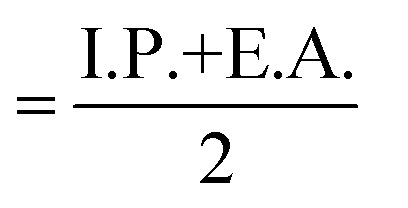
.

f Global hardness (*η*)
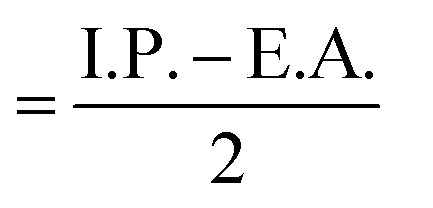
.

g Global softness (*S*) = 1/(2*n*).

h Electrophilicity index (*ω*) = *µ*^2^/(2*n*).

Conversely, a molecule with higher global hardness exhibits a greater resistance to changes in its electronic distribution during chemical reactions.^36^ The calculated global hardness (*η*) values for compounds 6a, 6b, and 6e, ranging from 3.3 to 3.5 eV, indicate that these molecules possess a significant resistance to charge transfer processes. The electronegativity (*χ*) of a molecule reflects its tendency to attract electrons, while a more negative chemical potential (*µ*) generally corresponds to enhanced stability in receptor binding.^39^ The studied compounds exhibit notably negative chemical potentials (−3.95 to −4.25 eV), suggesting higher sensitivity to variations in electron number and a stronger propensity to form stable interactions with biological targets. Furthermore, the electrophilicity index (*ω*) values for compounds 6a, 6b, and 6e, ranging from 2.30–2.71 eV, indicate a strong tendency to accept electrons from biomolecular receptors, thereby enhancing potential receptor interactions. The calculated dipole moments further support these findings, with compound 6b displaying the highest value (8.49 D) compared to 6a (5.10 D) and 6e (4.71 D), implying a greater degree of charge separation within its molecular framework and, consequently, stronger polar interactions with receptor sites.

#### 3.6.5. Local reactivity descriptor

**Fig. 13:**
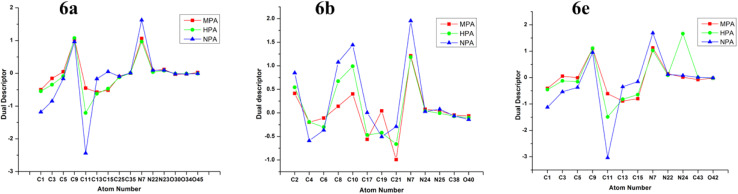
Variation of dual descriptor, at the selected atoms of compounds 6a, 6b and 6e based on MPA, HPA, and NPA charges based, calculated at B3LYP/6-31G (d,p) level of theory (eV).

The NPA charge distribution was employed to evaluate the Fukui functions, which serve as local reactivity indicators, for specific atoms in compounds 6a, 6b, and 6e that participate in binding interactions with the ERα receptor during docking studies. The Fukui function *f*^*+*^(*k*) represents an atom's ability to accept electrons, signifying its potential involvement with nucleophilic (electron-rich) residues, whereas *f*^−^(*k*) characterizes the electron-donating ability of an atom, indicating its possible interaction with electrophilic (electron-deficient) residues.^36^ The dual descriptor was determined using the expression *f*^2^(*k*) = [*f*^*+*^(*k*) – *f*^−^(*k*)], which provides an integrated perspective on whether an atom preferentially donates or accepts electrons.^40^ A positive *f*^2^(*k*) value designates an atom as an electron acceptor (electrophilic site), while a negative value indicates an electron donor (nucleophilic site). This analysis aids in pinpointing the reactive canters within each molecule that are likely to engage in electronic interactions with the receptor residues. The computed results revealed that, in both 6a and 6e, atoms such as C9 and N7 exhibited positive *f*^2^(*k*) values, identifying them as electrophilic centers, while C1, C3, and C11 displayed negative *f*^2^(*k*) values, suggesting their nucleophilic character. Additionally, atoms O30, O34, and O45 in 6a, O40 in 6b, and O42 in 6e were recognized as moderately reactive sites, likely contributing to complex stabilization through hydrogen bonding interactions. In compound 6b, the atoms C8, C10, and N7 were identified as preferred nucleophilic centers, whereas C4, C6, and C21 were characterized as electrophilic sites (Fig. 13).

#### 3.6.6. Natural bond orbital analysis (NBO)

The NBO study for compounds 6a, 6b, and 6e was carried out at the B3LYP/6-31G(d,p) level of theory in the gas phase to investigate the intramolecular charge transfer (ICT) phenomena (ESI, Tables S4–S6). The analysis was based on the second-order perturbation theory of the Fock matrix, which quantifies the donor–acceptor interactions in terms of the associated stabilization energy *E*^(2)^. Higher *E*^(2)^ values signify more efficient electron delocalization between bonding and antibonding orbitals, indicating strong hyperconjugative effects and enhanced electronic communication within the molecular system.^41^ The results of NBO analysis showing a significant hyperconjugative interactions: LP(2)O19 → π*(C17–O18), LP(2)O41 → π*(O42–C43), LP(2)O33 → π*(O34–C35), LP(1)N24 → π*(N22–N23), LP(2)O37 → π*(O38–C39), LP(2)O34 → σ*(O33–C35), LP(2)O38–σ*(O37 – C39), LP(2)O42 → σ*(O41–C43), in both 6a and 6e. Some more high energy hyperconjugative interactions were LP(2)O45 → π*(O46–C47), LP(2)O46 → σ*(O45–C47) in 6a and LP(2)O46 → π*(O47–C48), LP(2)O47 → σ*(O46–C48) in 6e. The most prominent interactions in compound 6b were LP(2)O14 → π*(C12–O13), LP(2)O36 → π*(C44–O46), LP(2)O35 → π*(C41–O43), LP(2)O37 → π*(C38–O40), LP(1)N23 → π*(N24–N25), LP(2)O13 → σ*(C12–O14), LP(2)O40 → σ*(O37–C38), LP(2)O43 → σ*(O35–C41), LP(2)O46 → σ*(O36–C44), LP(2)O48 → σ*(O34–C47). Other high energy stabilization interactions are π*(N7–C8) → π*(C9–C10), π(C21–C25) → π*(N22–N23), π (C9–C10) → π*(N7–C8), π(C15–C16) → π*(C13–C14), in both 6a and 6e, and π*(N7–C8) → π*(C9–C10), π(C16–C22) → π*(N24–N25), and π (C9–C10) → π*(C12–O13), π (C11–C21) → π*(C19–C20), π(N7–C8) → π*(C4–C5), in 6b.

## 4. Conclusion

A novel series of phenyl-substituted quinoline–1,2,3-triazole glycoconjugates (6a–6k) was efficiently synthesized using a Cu(i)-catalyzed azide–alkyne cycloaddition (CuAAC) strategy, demonstrating the versatility of this approach for incorporating diverse sugar azides to phenyl-substituted quinoline precursors. The synthesized glycoconjugates were fully characterized by spectroscopic techniques, and the structures of selected derivatives (6a, 6b, and 6e) were unequivocally confirmed through single-crystal X-ray diffraction. Crystallographic analysis revealed that intermolecular hydrogen-bonding interactions play a significant role in stabilizing the crystal packing. *In vitro* evaluation against the MCF-7 breast cancer cell line revealed that, while most compounds were inactive, a few derivatives (6f, 6c, and 6d) exhibited weak cytotoxicity, indicating the need for further structural optimization to enhance biological activity. Molecular docking studies showed favourable binding interactions with estrogen receptor alpha (ERα), supporting their potential as ERα-interacting scaffolds. Complementary DFT analyses provided detailed insights into the electronic structure, stability, reactivity, and charge distribution of the compounds, contributing to a deeper understanding of their chemical behaviour.

## Conflicts of interest

The authors declare that the research was conducted in the absence of any commercial or financial relationships that could be construed as a potential conflict of interest.

## Supplementary Material

RA-016-D5RA09700B-s001

RA-016-D5RA09700B-s002

## Data Availability

CCDC 2457702, 2424982 and 2445773 contain the supplementary crystallographic data for this paper.^42*a*–*c*^ All data supporting the findings of this study are provided in the supporting information (SI). Supplementary information: including detailed experimental procedures, complete characterization data, NMR and HRMS spectra, X-ray crystallographic analyses, and DFT computational results. See DOI: https://doi.org/10.1039/d5ra09700b.
