# Supplementary material for: Design, synthesis, structural characterization, anticancer evaluation, and computational studies of novel quinoline-1,2,3-triazole glycohybrids
Source: RSC Adv. 2026 Feb 11;16(10):8466–86. doi: 10.1039/d5ra09700b (PMC12893069; doi:10.1039/d5ra09700b)
Supplement: RA-016-D5RA09700B-s001 [file RA-016-D5RA09700B-s001.pdf]

Supplementary Information (SI) for RSC Advances.
This journal is © The Royal Society of Chemistry 2026

Electronic Supporting Information

for

Synthesis, Structural Characterization, Anticancer Evaluation, and Computational Studies of Novel Quinoline-1,2,3-Triazole Glycohybrids

Ravendra Kumar[a], Deepanshi Chauhan[a], Rakesh Kumar Gupta[b], Pawan K. Dubey[b], and Divya Kushwaha[a*]

[a] Department of Chemistry, MMV, Banaras Hindu University, Varanasi-221005, India.
[b]Centre for Genetic Disorder, I.Sc., Banaras Hindu University, Varanasi-221005, India.
Corresponding author email. divyakush.mmvbhu@ac.in

S. No.	Contents	Page No.
1.	NMR, HRMS and FT-IR Spectra	2-32
2.	X-ray Crystallography study	33
3.	Molecular Docking	34
4.	HirshField and 2D Analysis	35-37
5.	DFT studies	38-43

1. NMR and HRMS spectra of Compounds

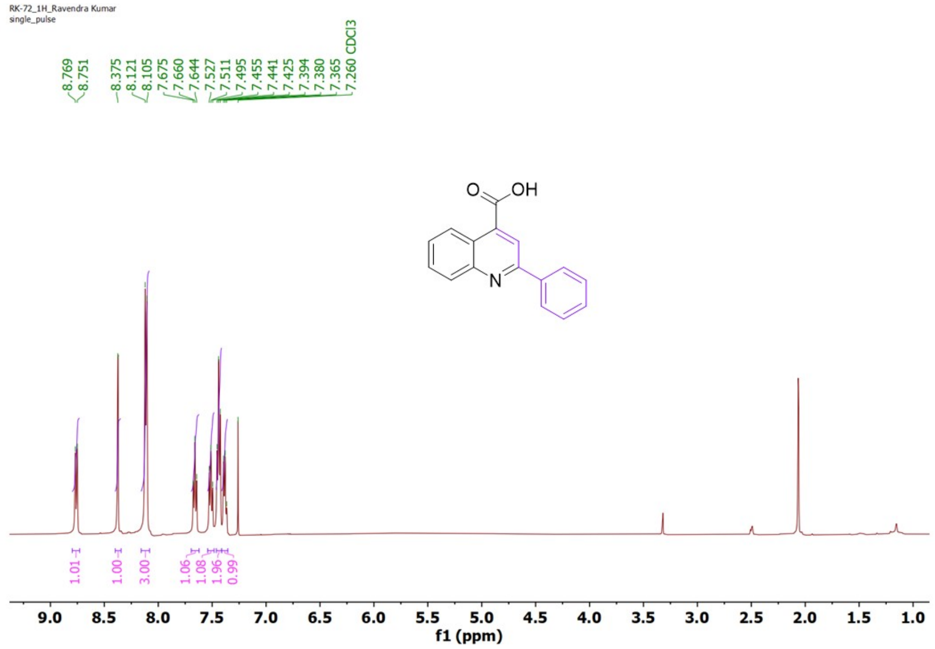

Fig. S1: ^{1}H NMR spectrum of **3a**

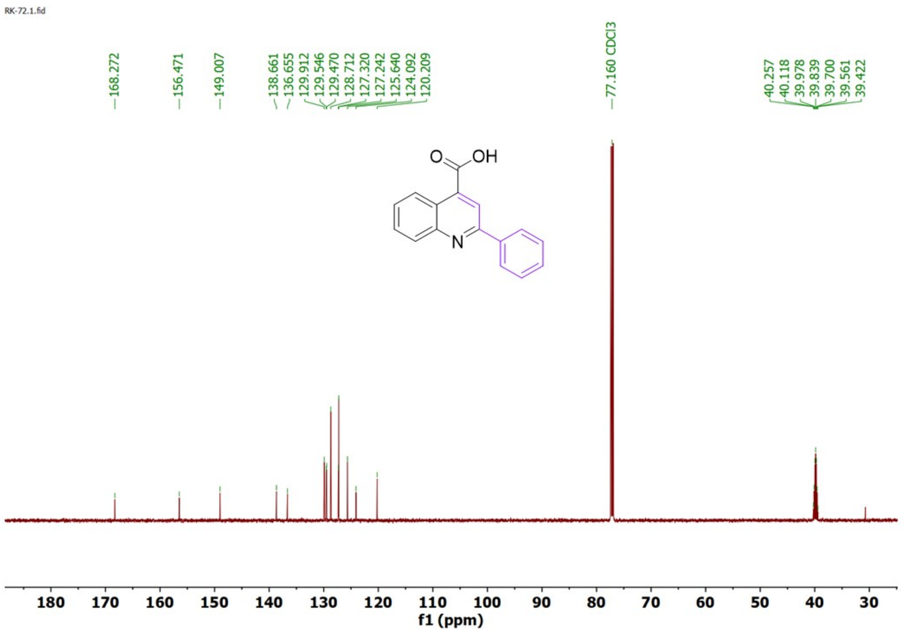

Fig. S2: ^{13}C NMR spectrum of **3a**

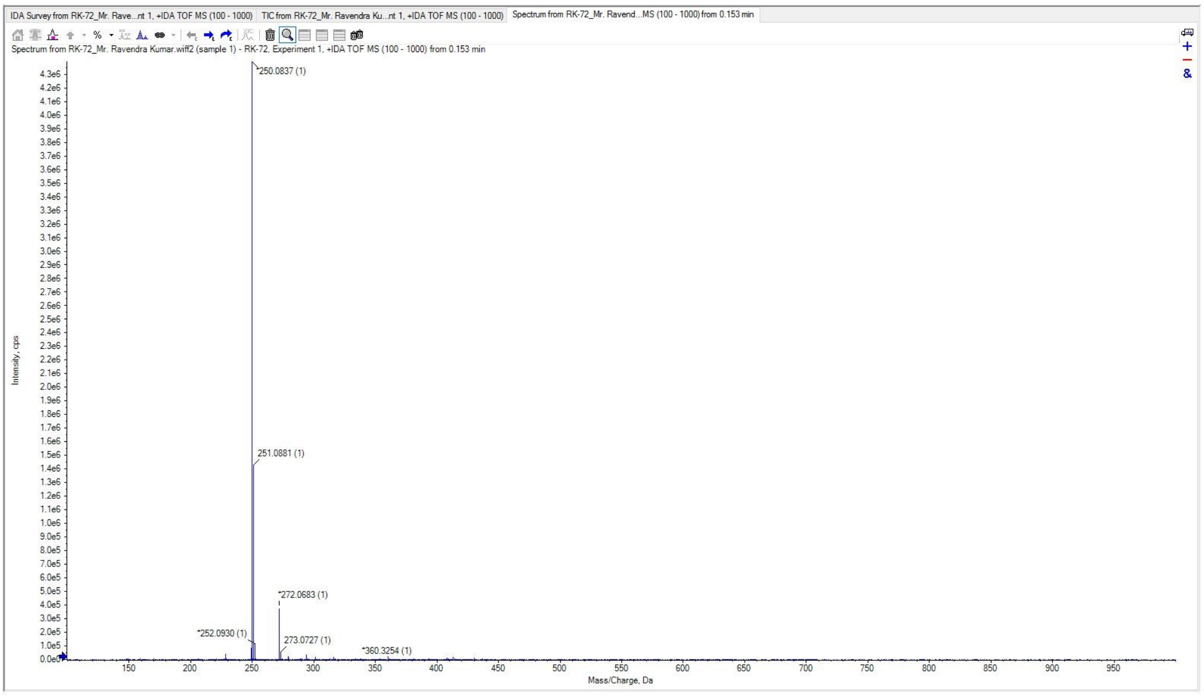

Fig. S3: HRMS spectrum of **3a**

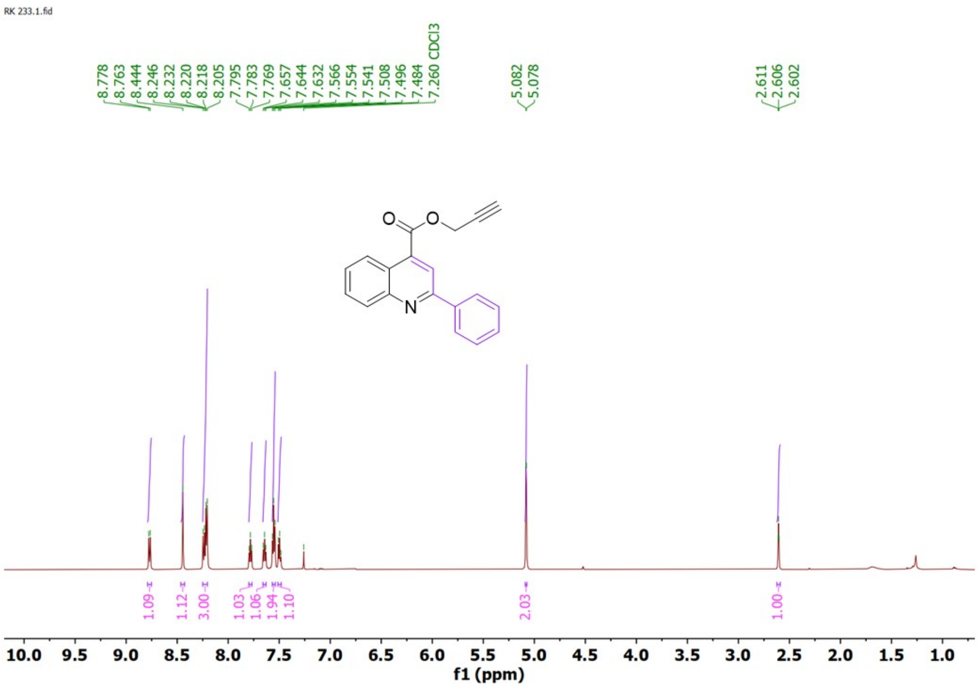

Fig. S4: ^{1}H NMR spectrum of **4a**

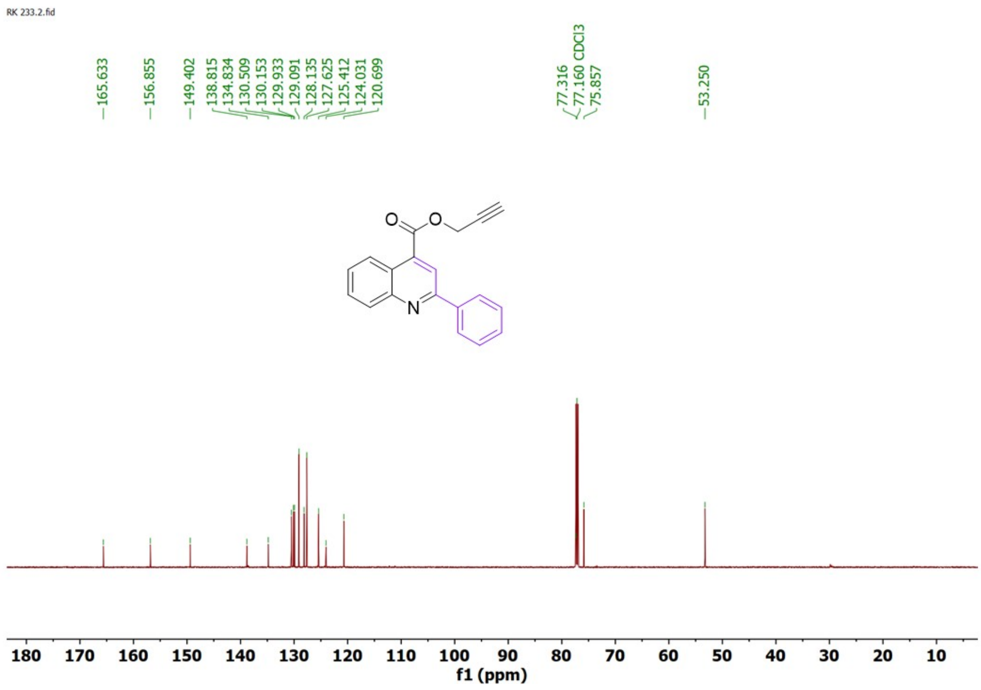

Fig. S5: ^{13}C NMR spectrum of **4a**

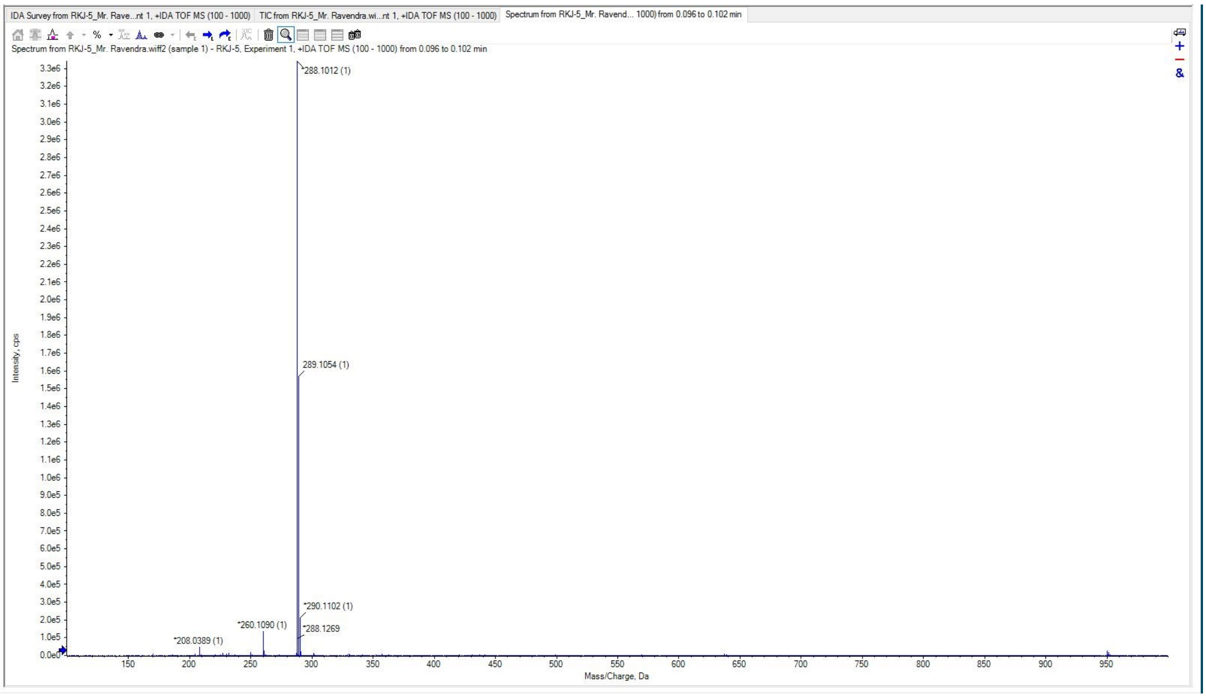

Fig. S6: HRMS spectrum of **4a**

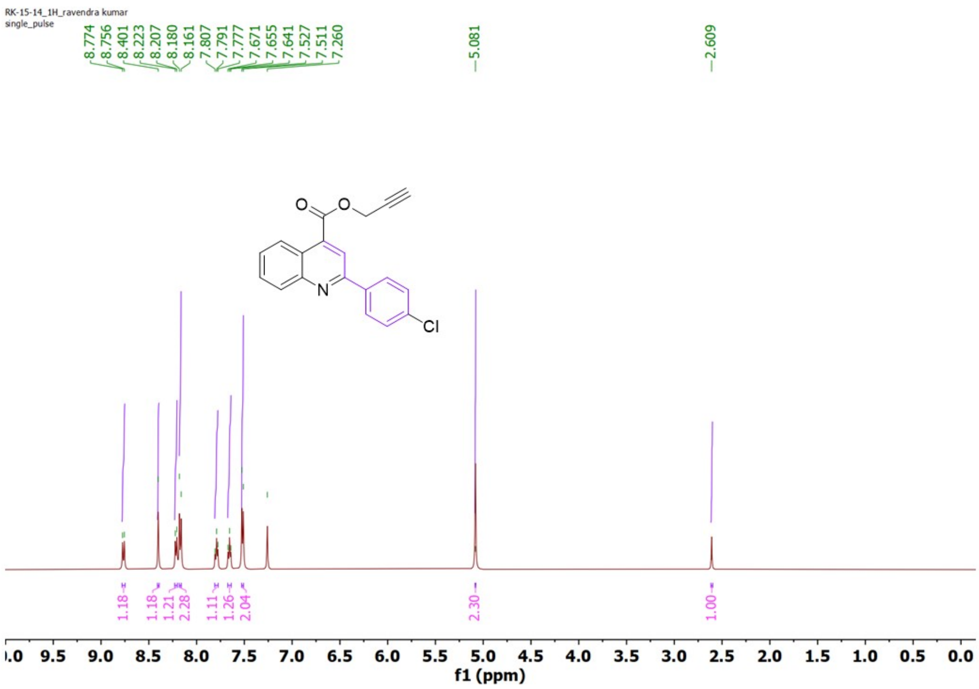

Fig. S7: ^{1}H NMR spectrum of **4b**

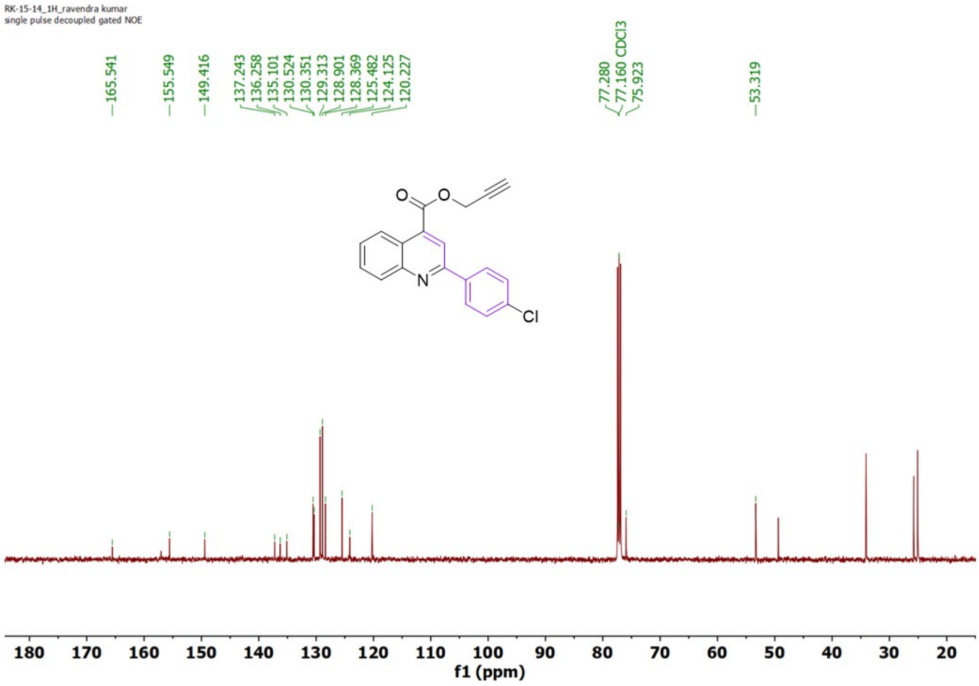

Fig. S8: ^{13}C NMR spectrum of **4b**

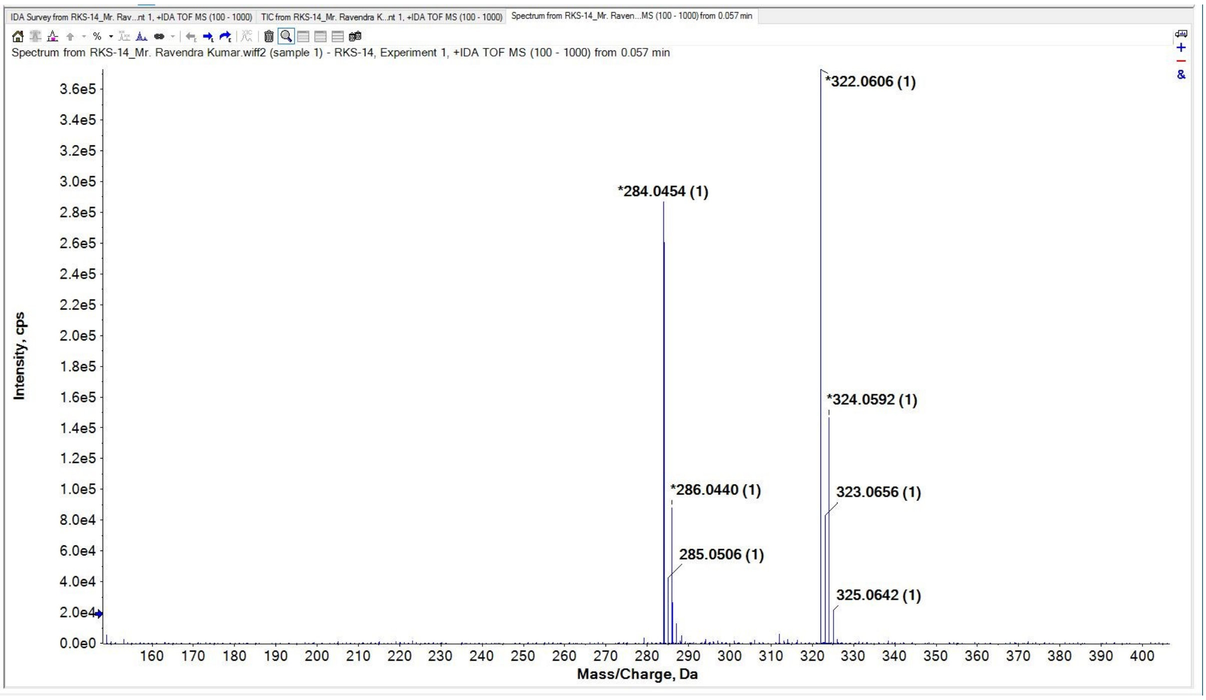

Fig. S9: HRMS spectrum of **4b**

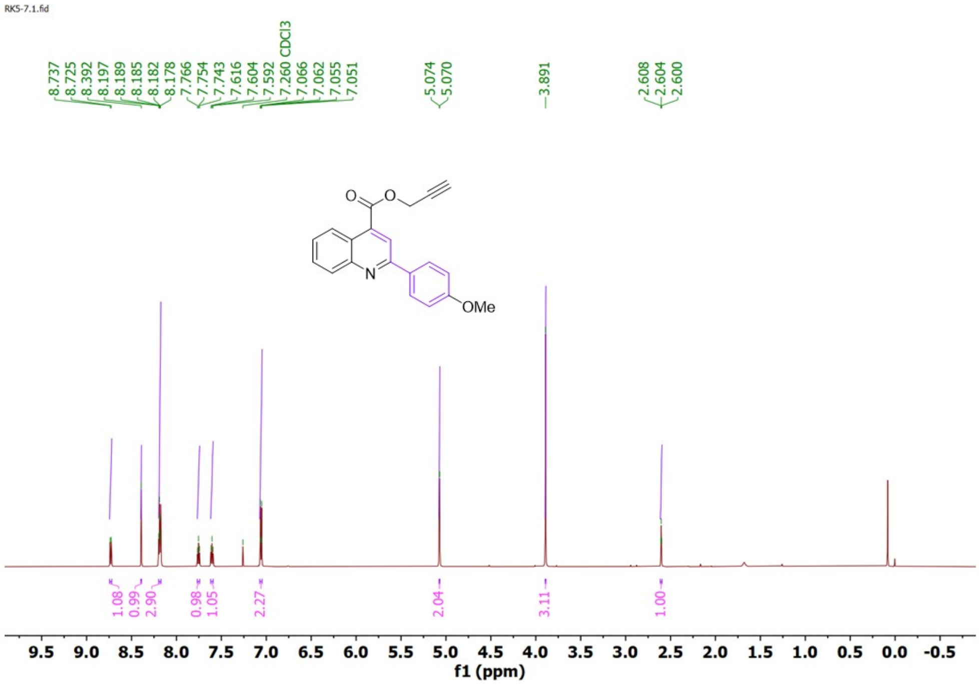

Fig. S10: ^{1}H NMR spectrum of **4c**

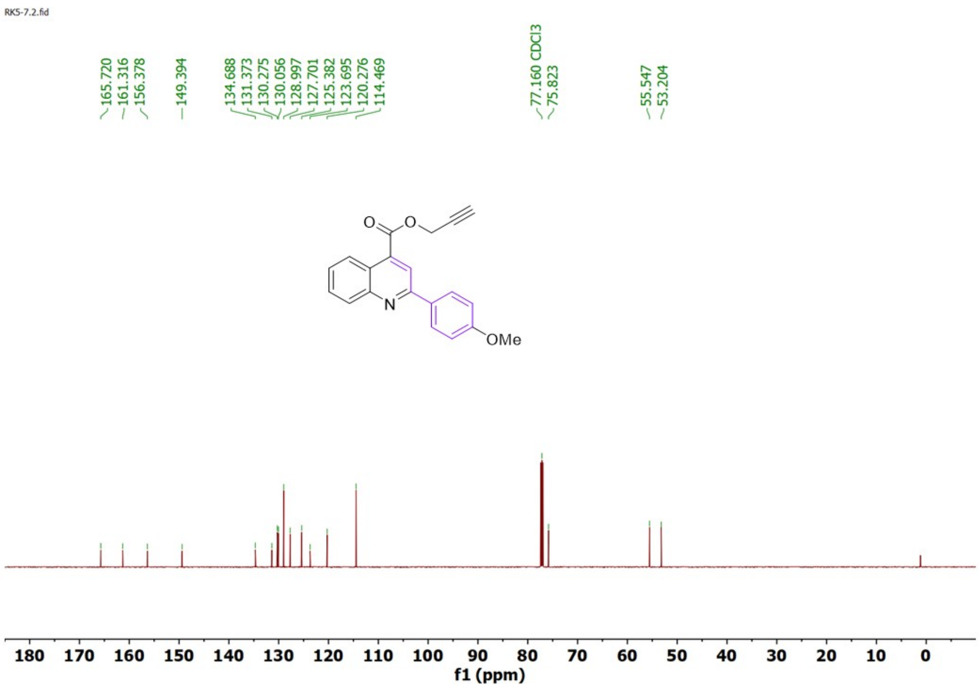

Fig. S11: ^{13}C NMR spectrum of **4c**

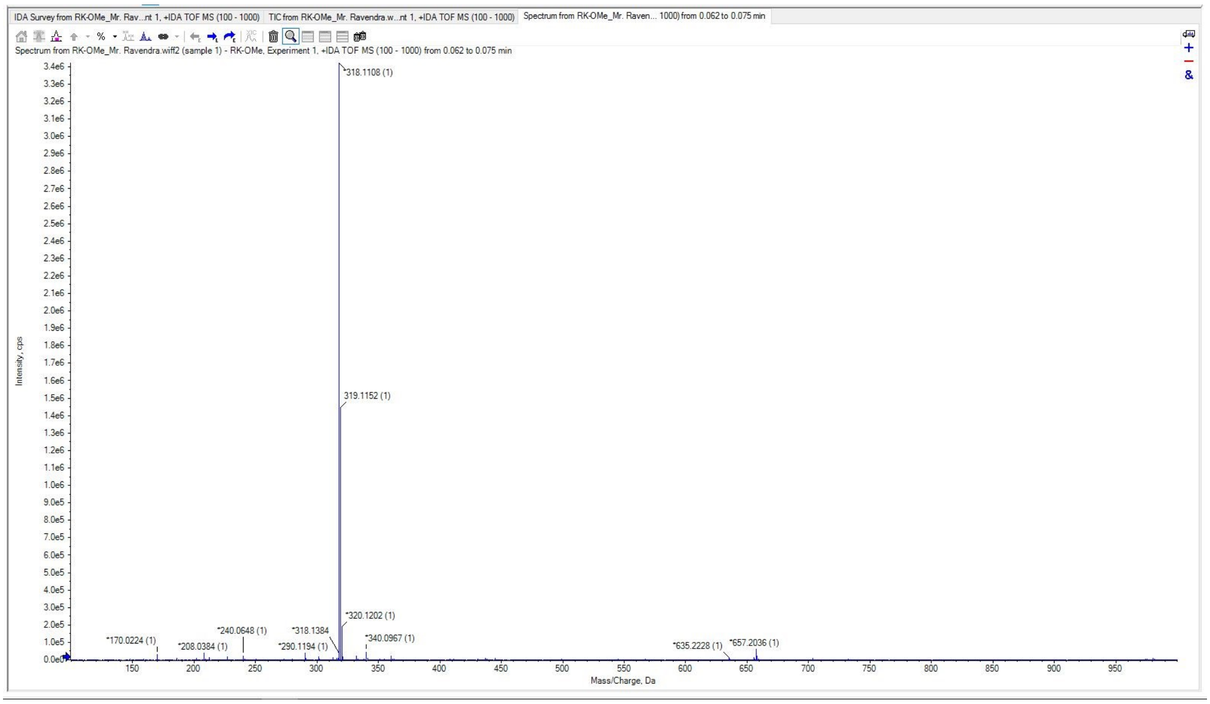

Fig. S12: HRMS spectrum of **4c**

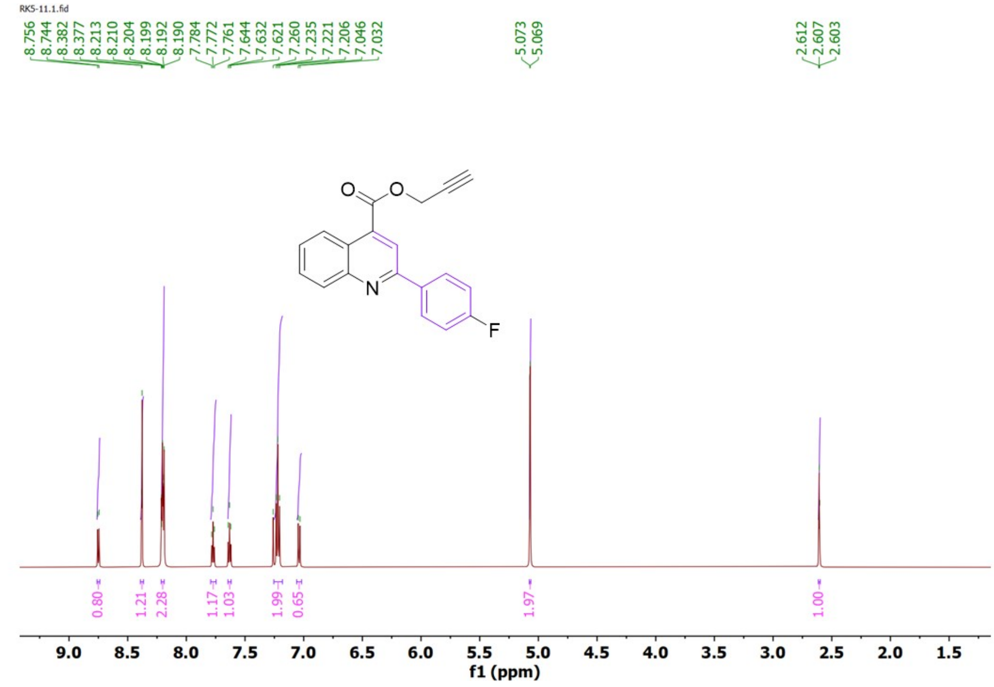

Fig. S13: ^{1}H NMR spectrum of **4d**

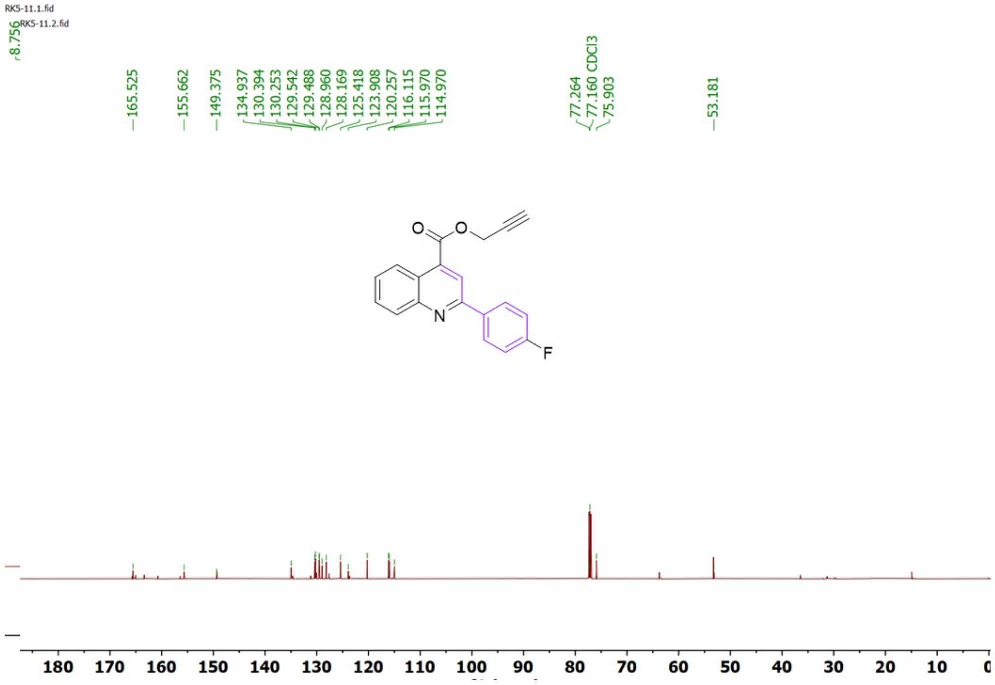

Fig. S14: ^{13}C NMR spectrum of **4d**

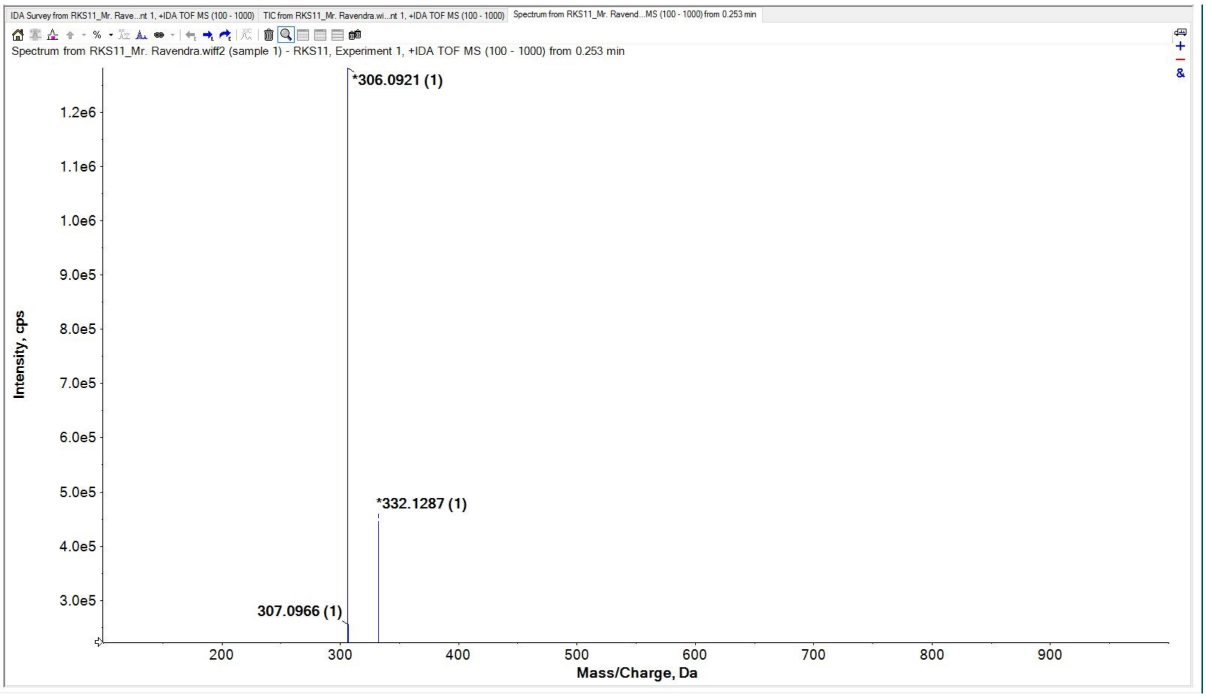

Fig. S15: HRMS spectrum of **4d**

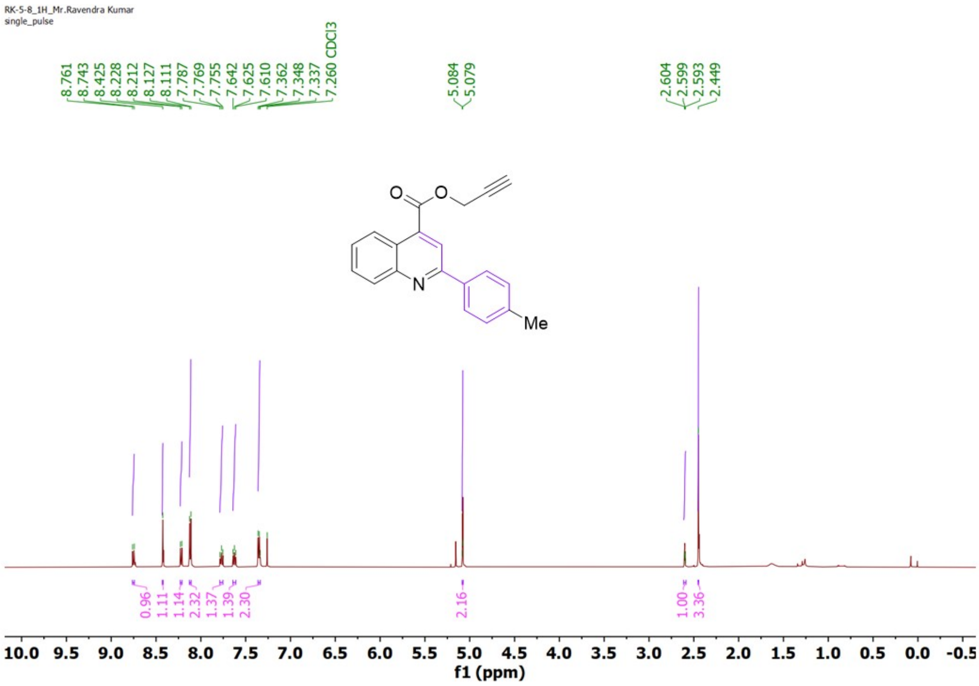

Fig. S16: ^{1}H NMR spectrum of **4e**

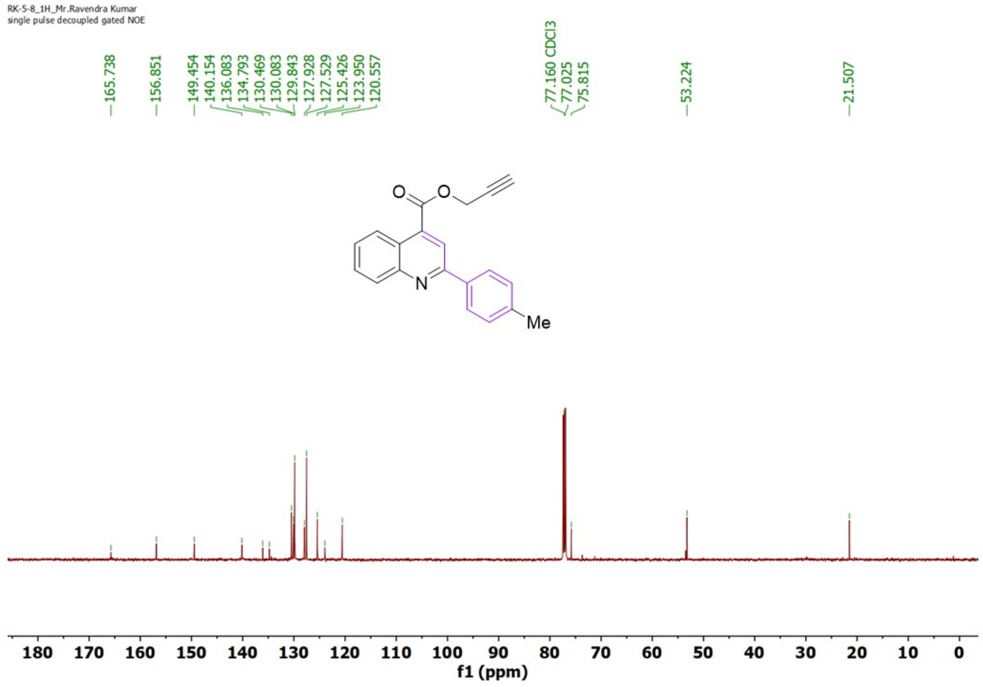

Fig. S17: ^{13}C NMR spectrum of **4e**

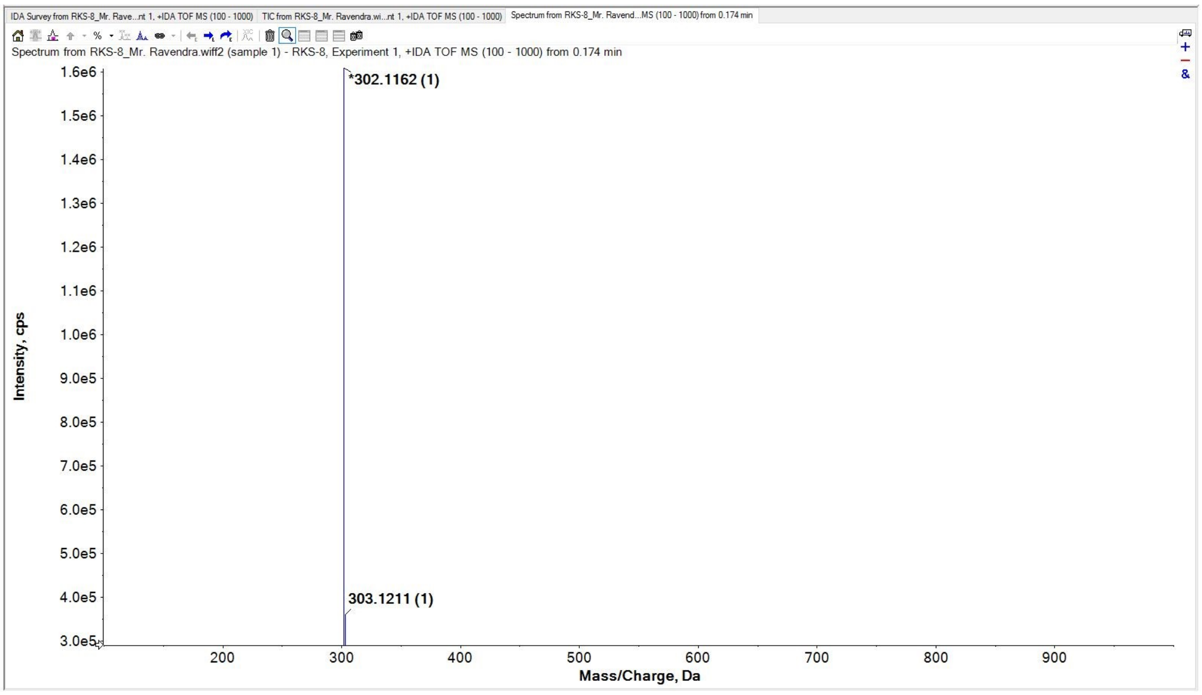

Fig. S18: HRMS spectrum of **4e**

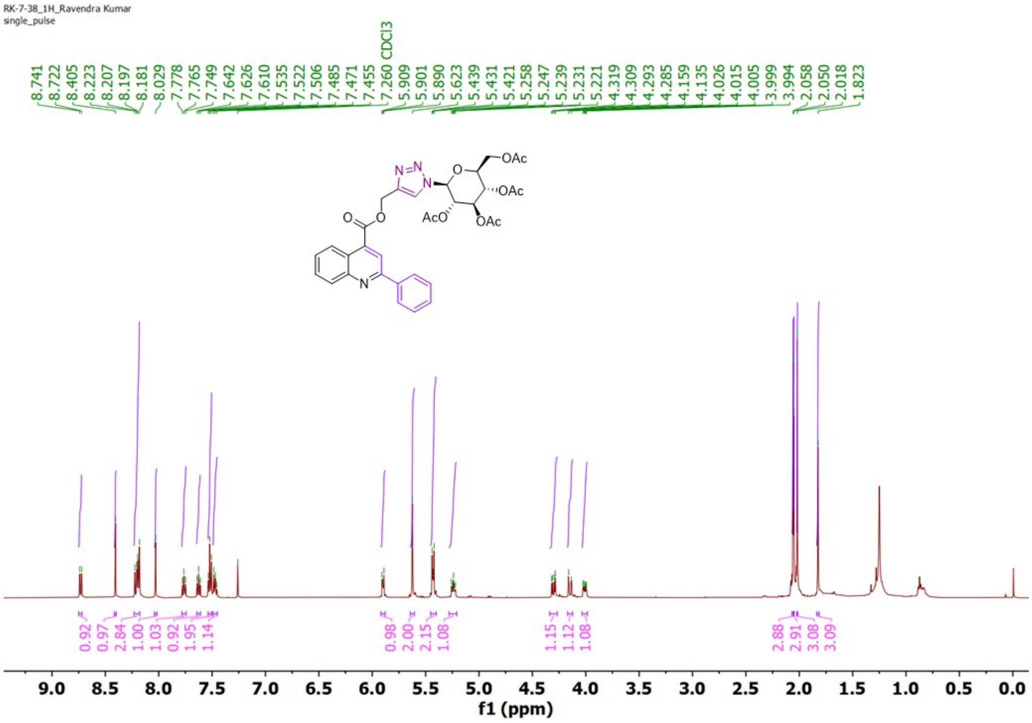

Fig. S19: ^{1}H NMR spectrum of **6a**

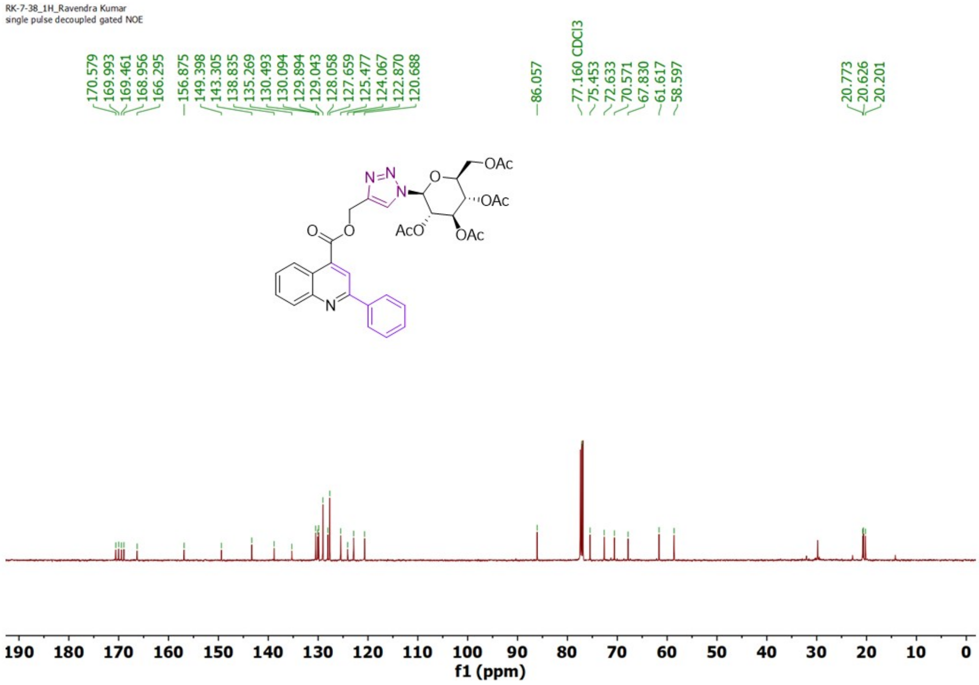

Fig. S20: ^{13}C NMR spectrum of **6a**

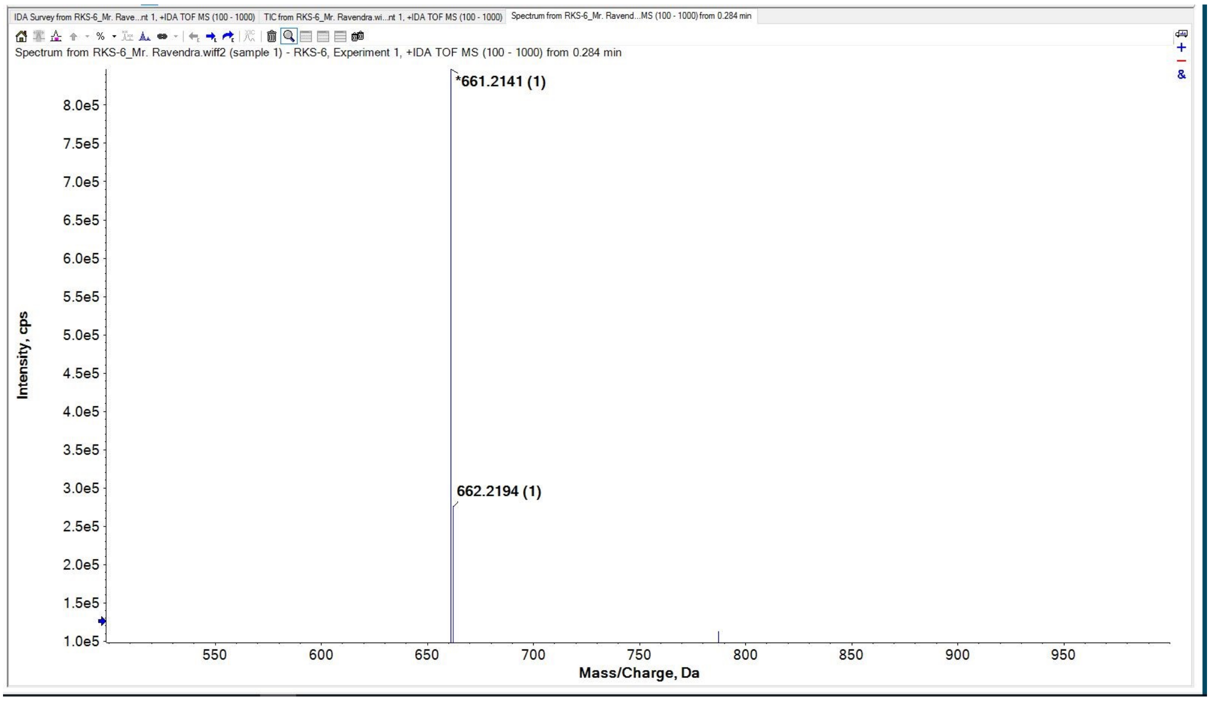

Fig. S21: HRMS spectrum of **6a**

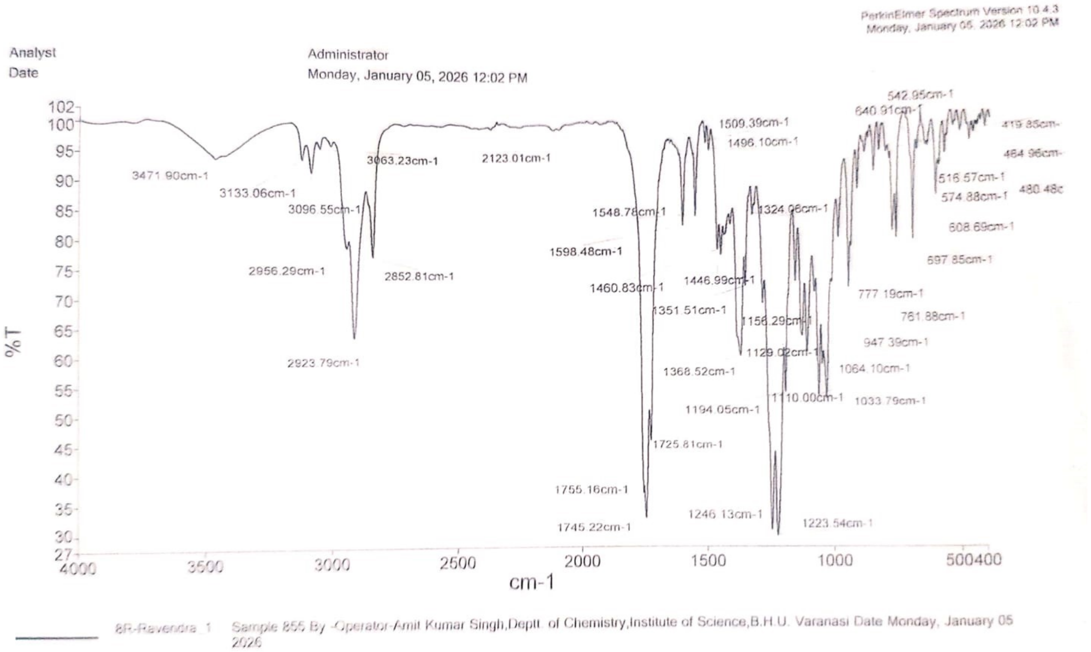

Fig. S22: FT-IR spectrum of **6a**

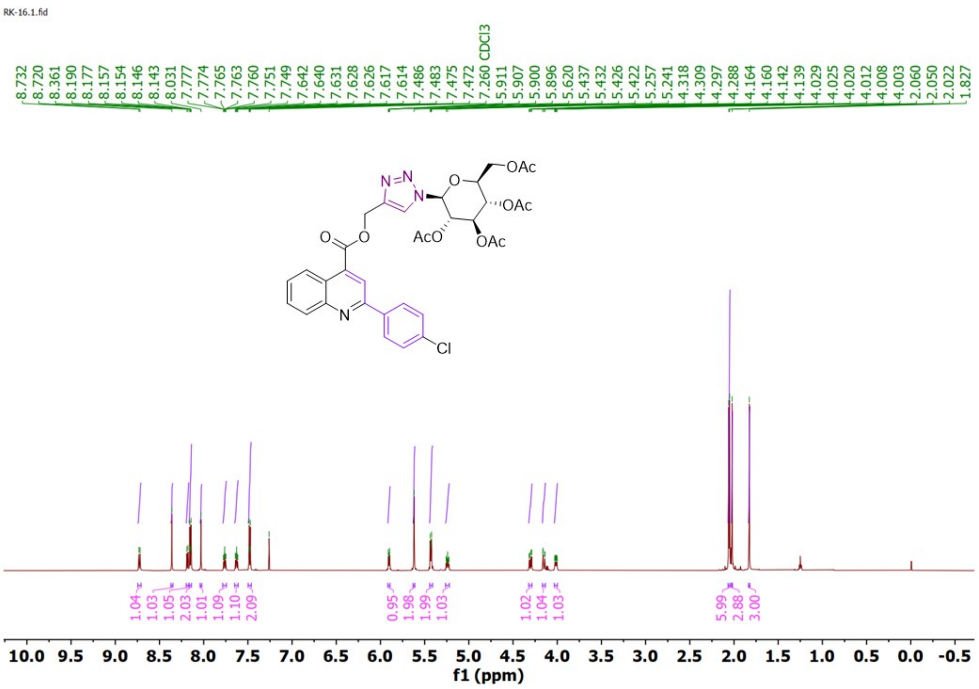

Fig. S23: ^{1}H NMR spectrum of **6b**

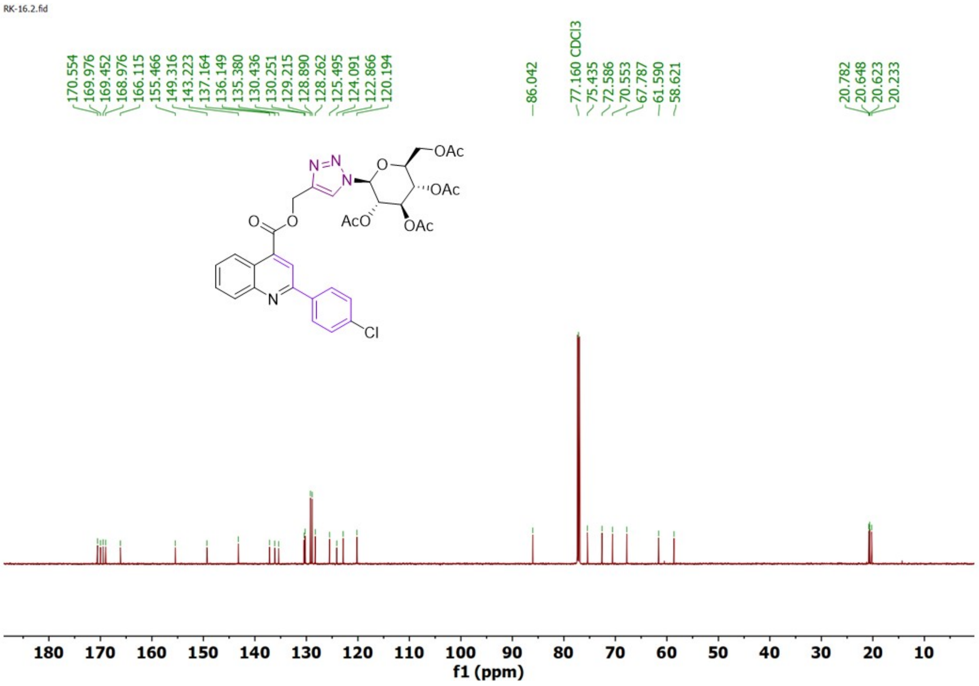

Fig. S24: ^{13}C NMR spectrum of **6b**

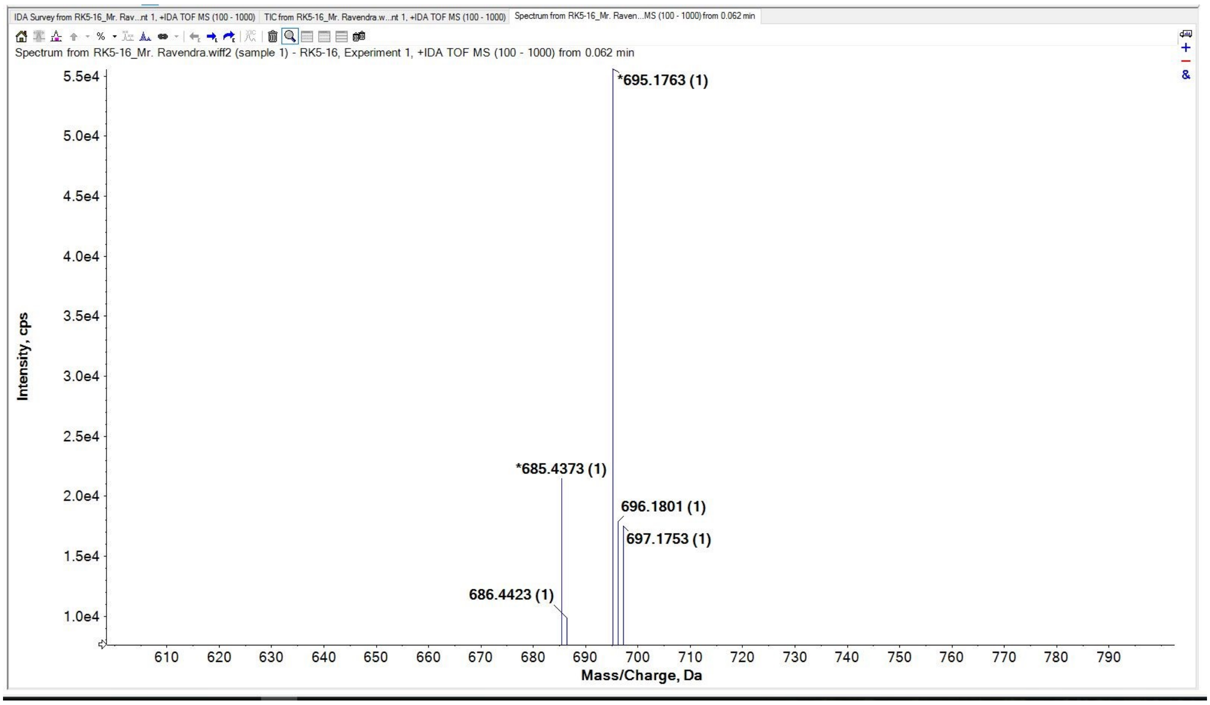

Fig. S25: HRMS spectrum of **6b**

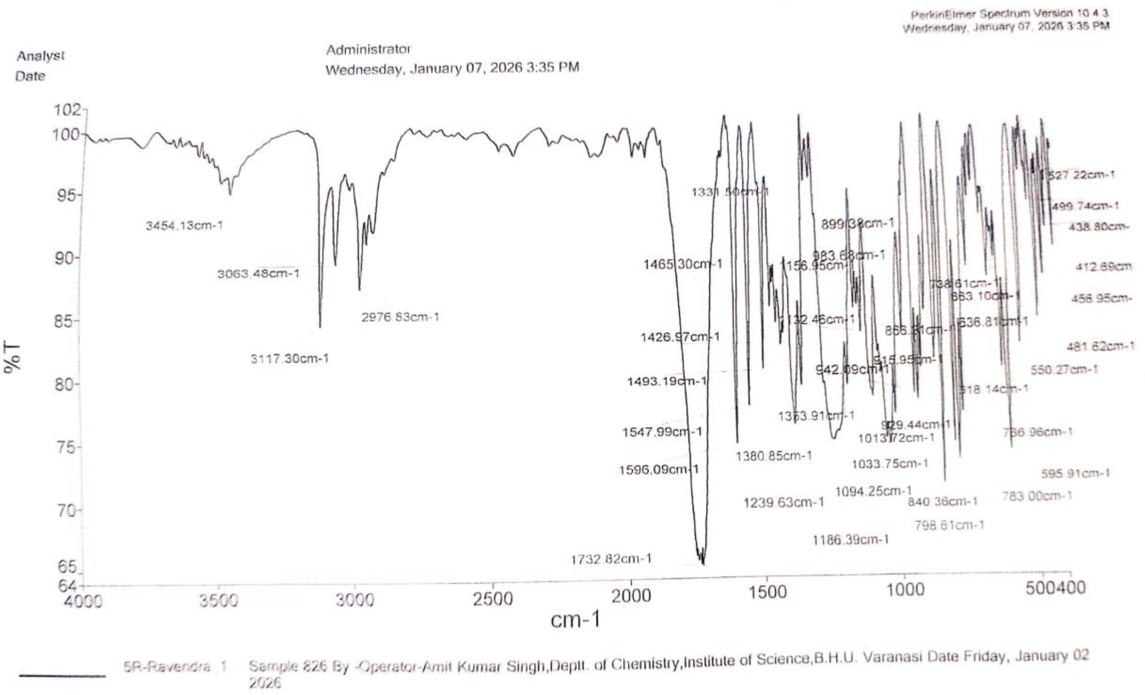

Fig. S26: FT-IR spectrum of **6b**

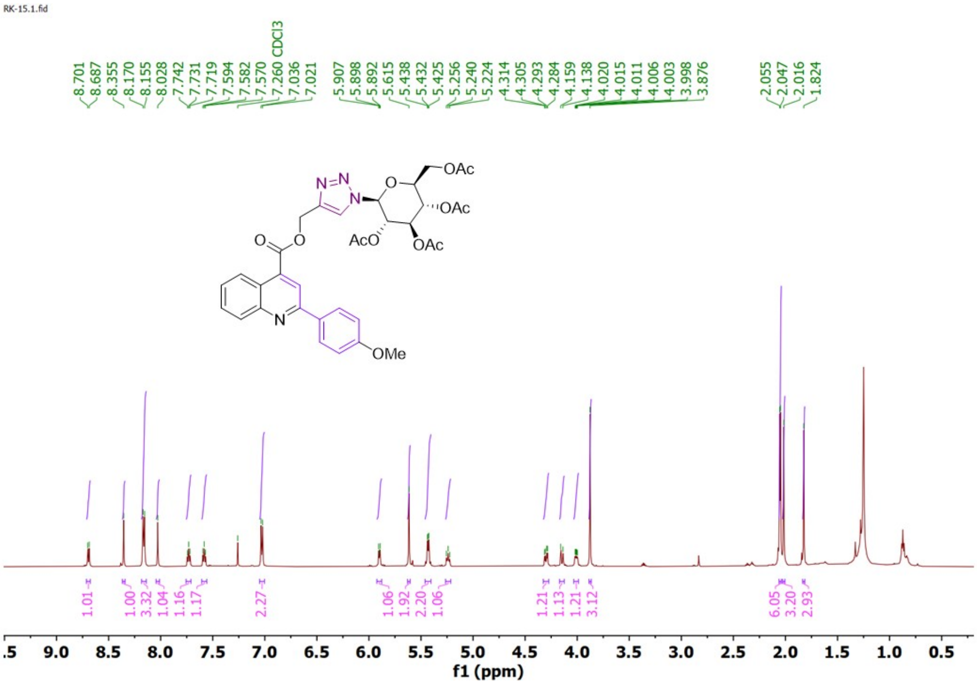

Fig. S27: ^{1}H NMR spectrum of **6c**

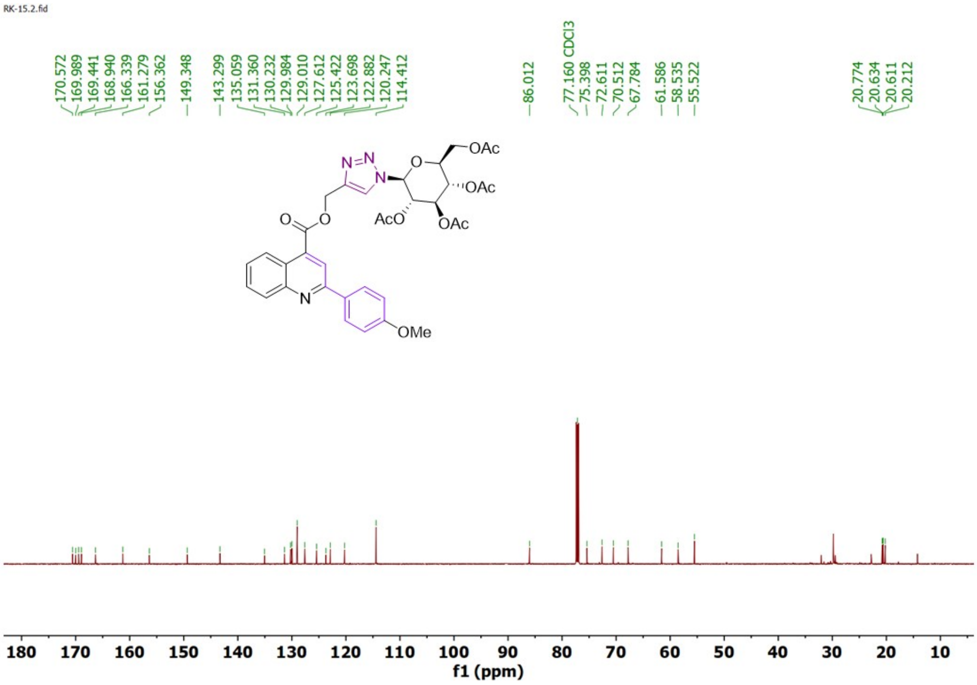

Fig. S28: ^{13}C NMR spectrum of **6c**

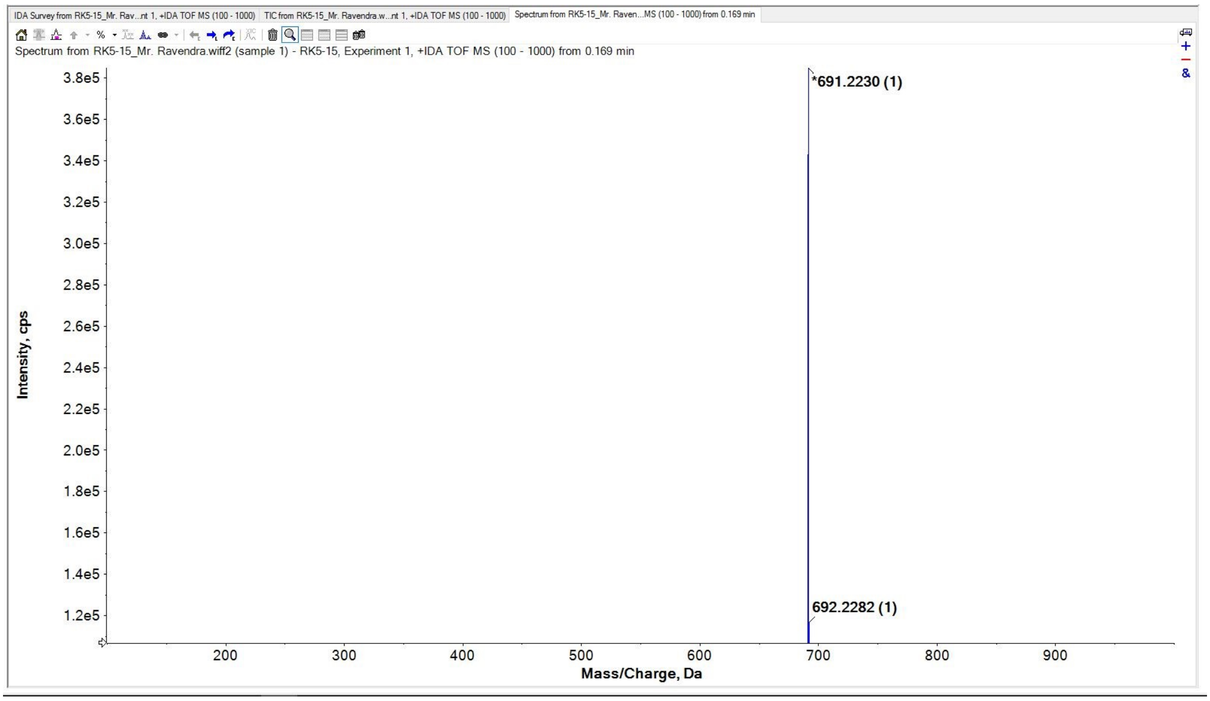

Fig. S29: HRMS spectrum of **6c**

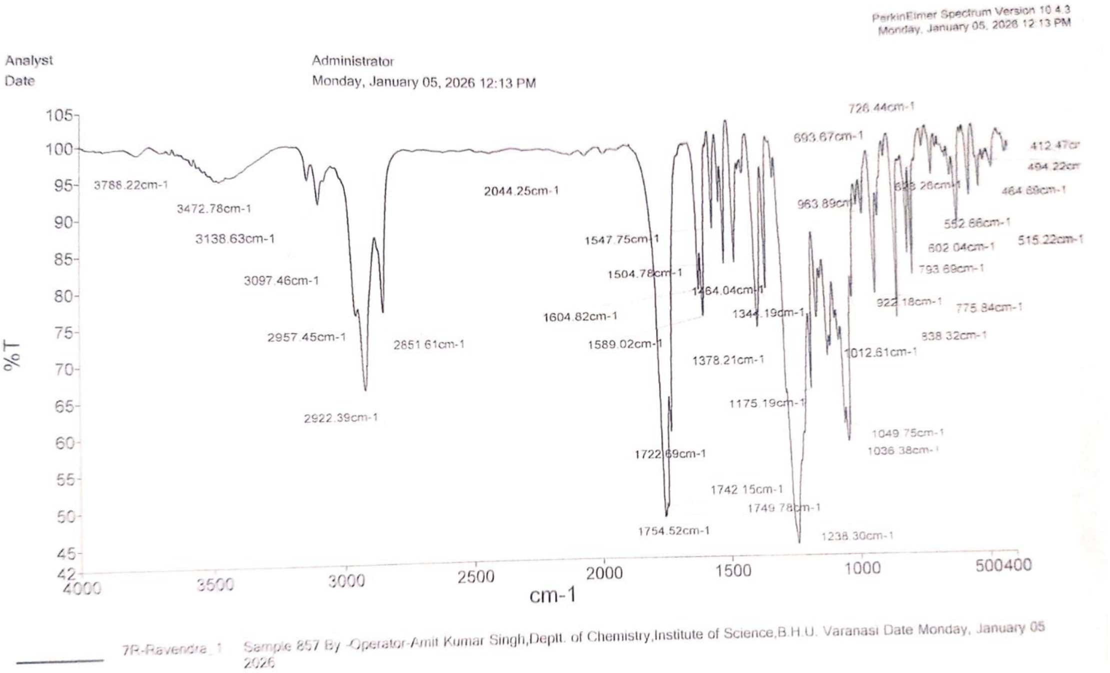

Fig. S30: FT-IR spectrum of **6c**

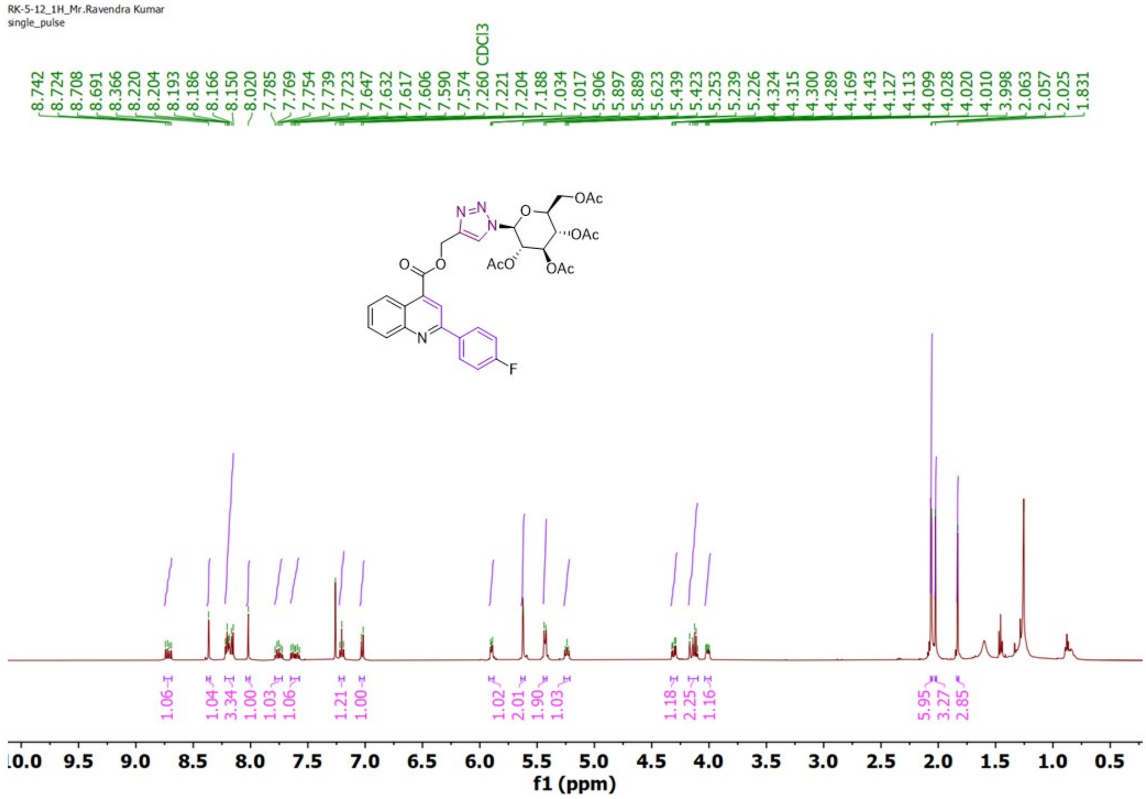

Fig. S31: ^{1}H NMR spectrum of **6d**

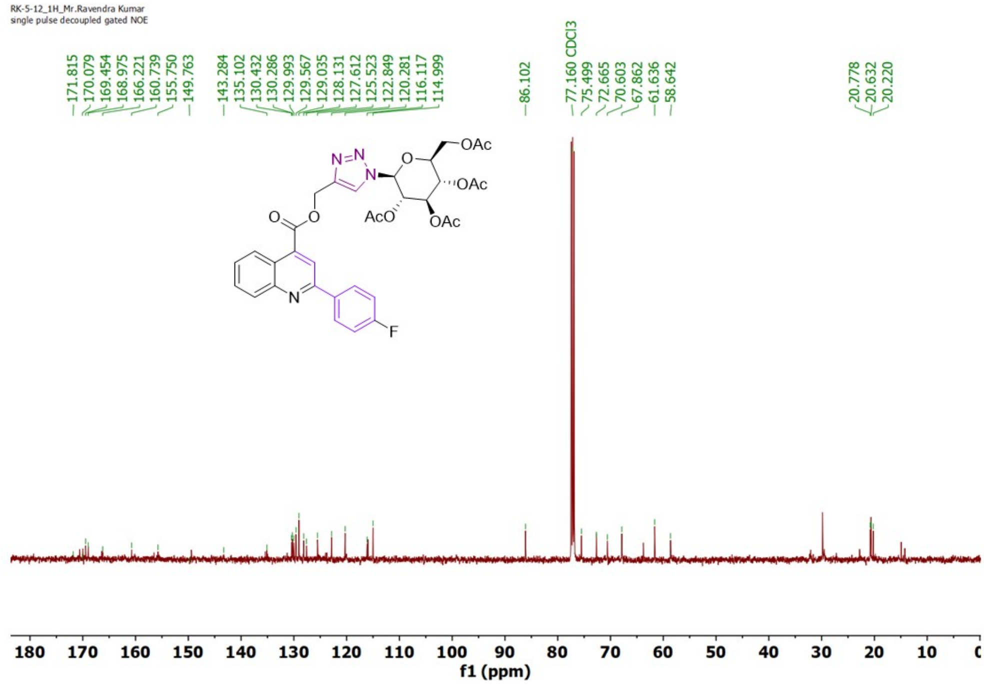

Fig. S32: ^{13}C NMR spectrum of **6d**

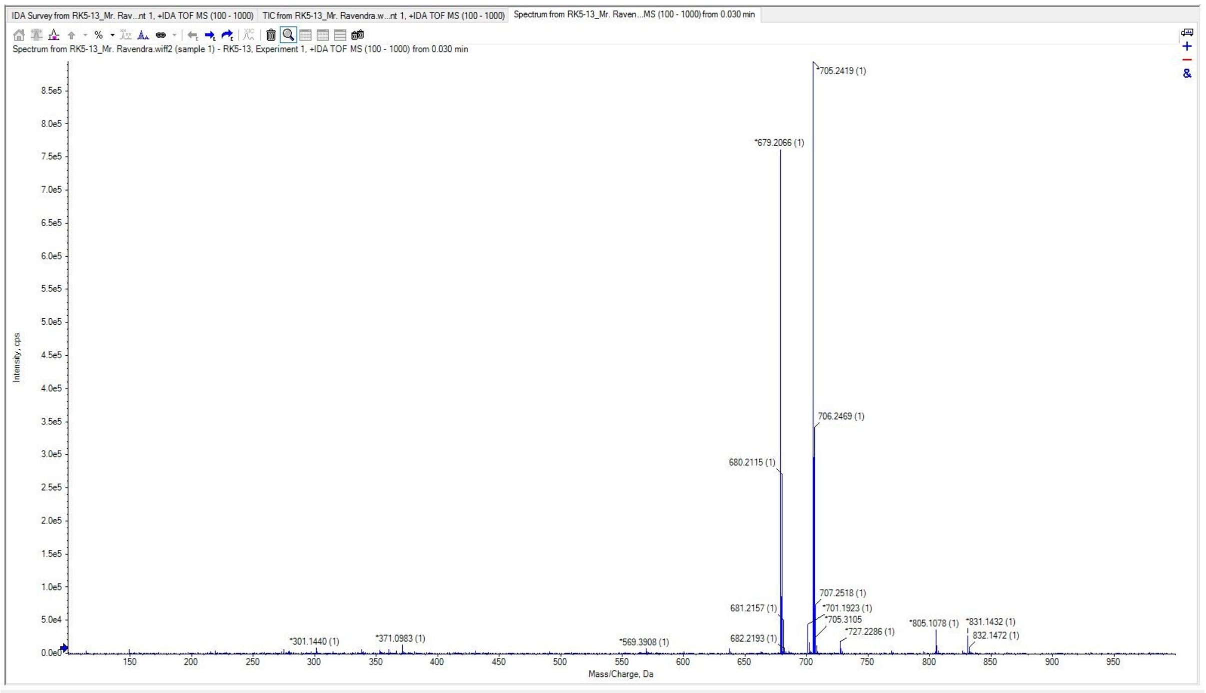

Fig. S33: HRMS spectrum of **6d**

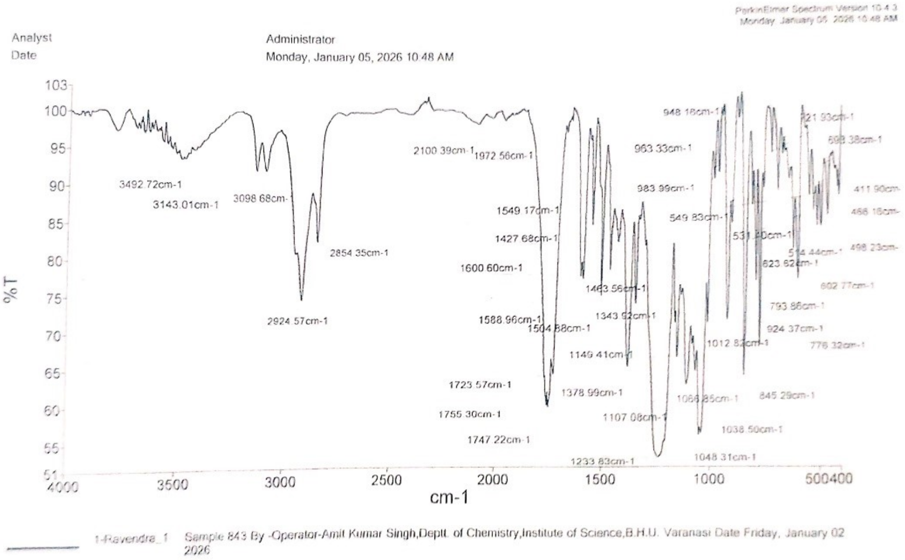

Fig. S34: FT-IR spectrum of **6d**

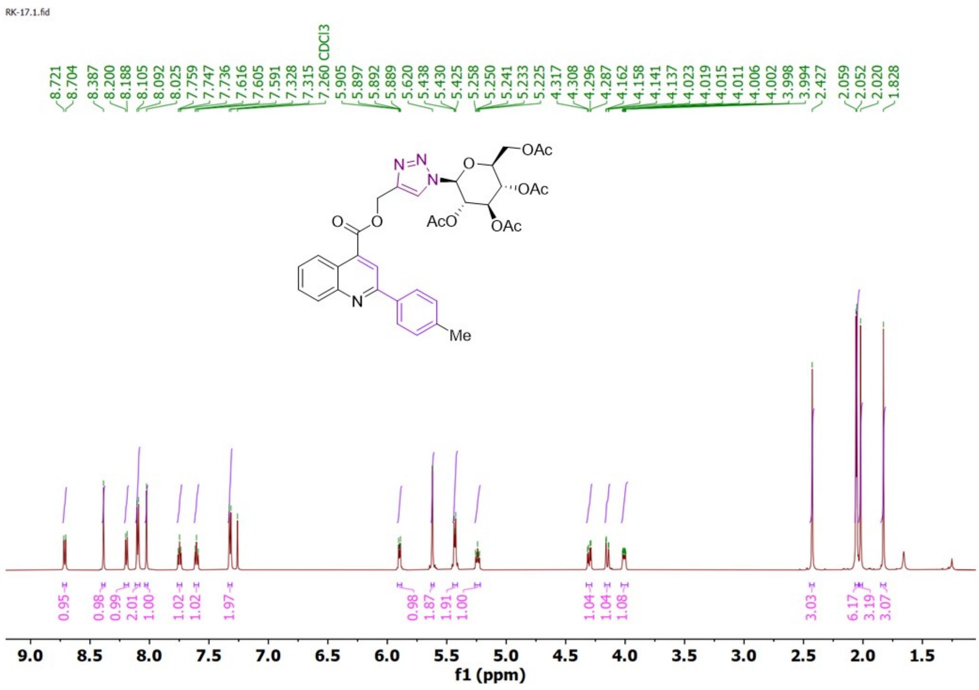

Fig. S35: ^{1}H NMR spectrum of **6e**

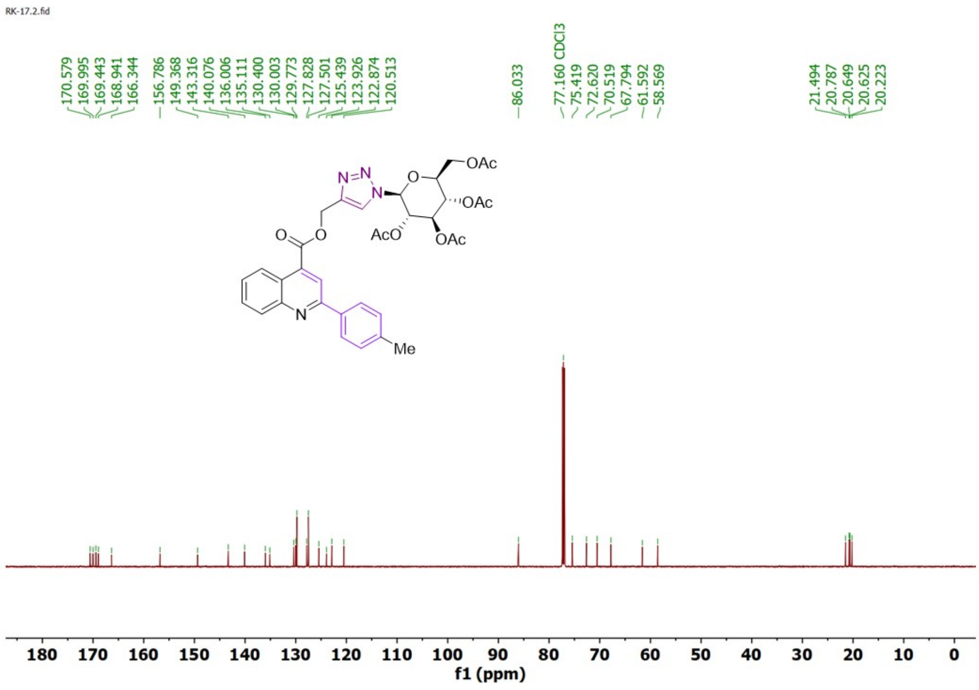

Fig. S36: ^{13}C NMR spectrum of **6e**

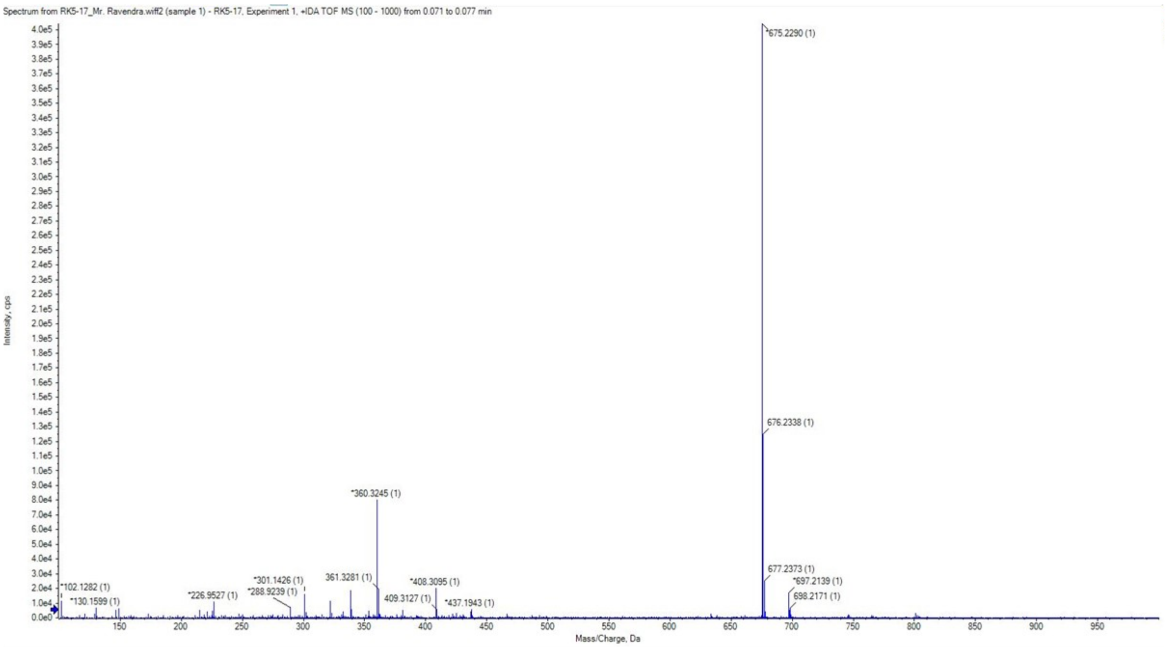

Fig. S37: HRMS spectrum of **6e**

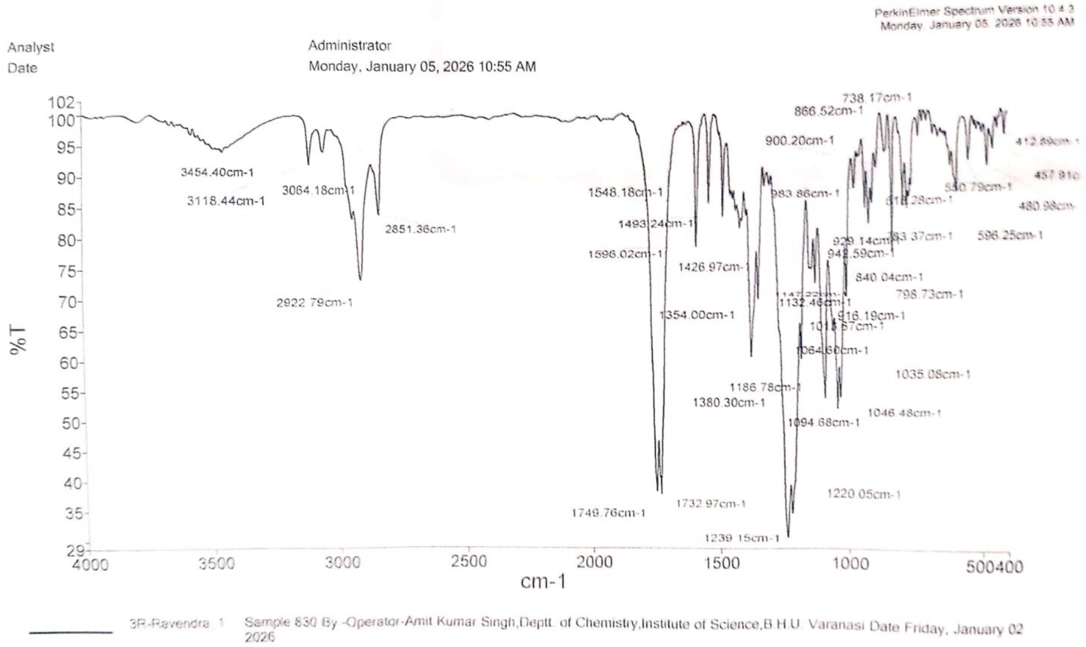

Fig. S38: FT-IR spectrum of **6e**

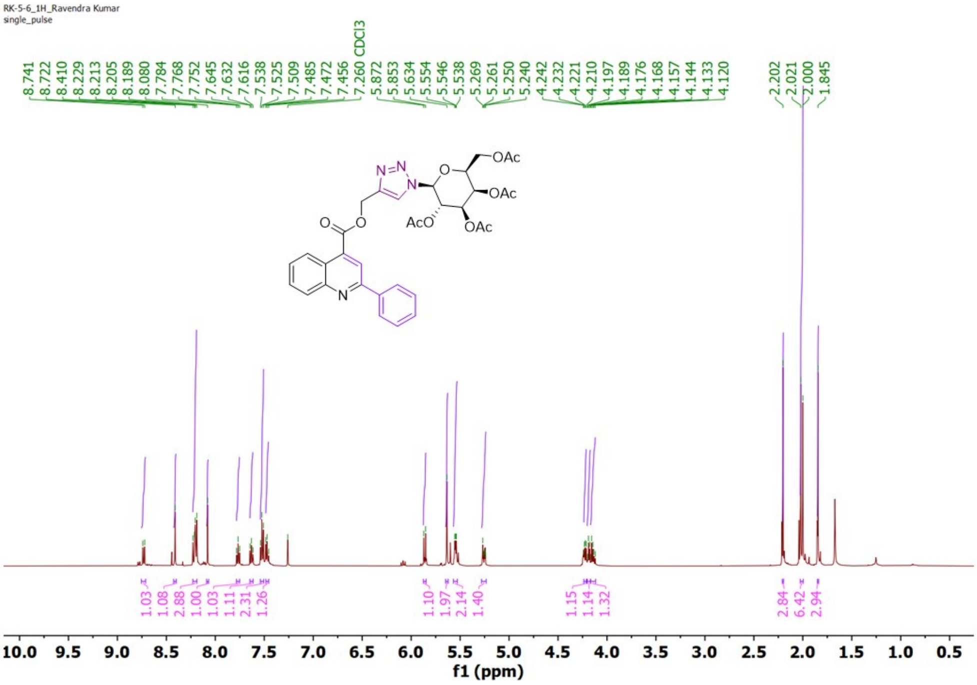

Fig. S39: ^{1}H NMR spectrum of **6f**

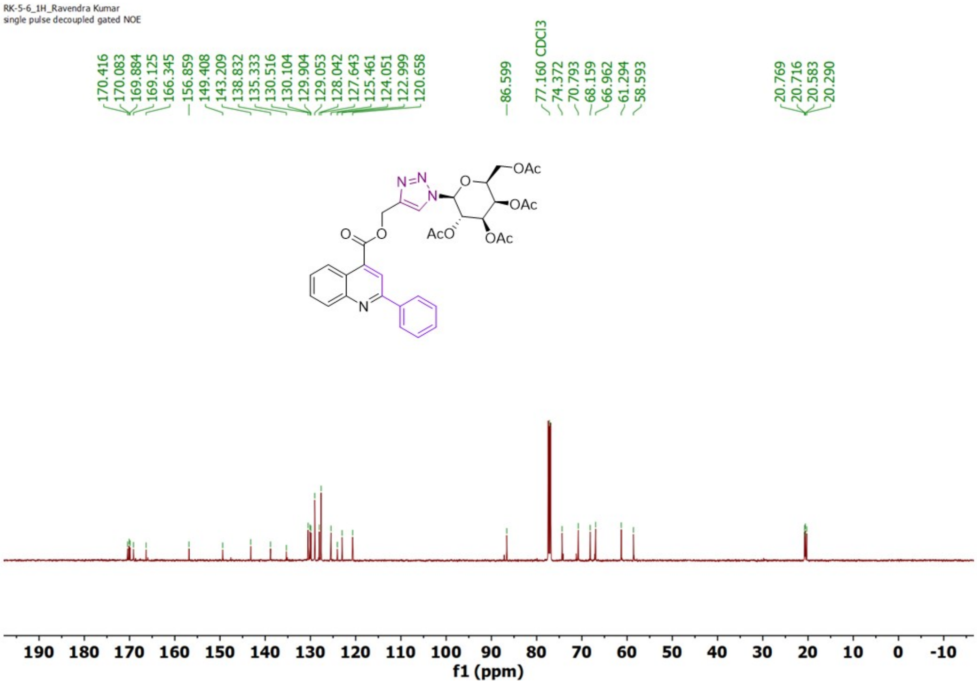

Fig. S40: ^{13}C NMR spectrum of **6f**

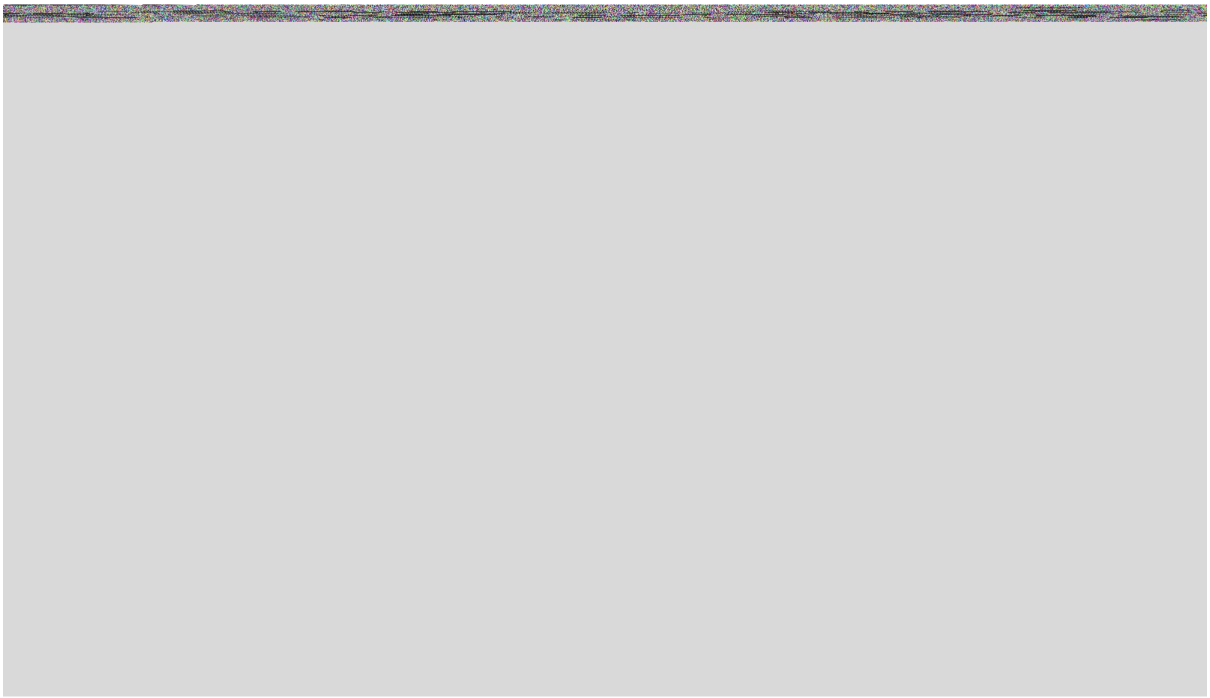

Fig. S41: HRMS spectrum of **6f**

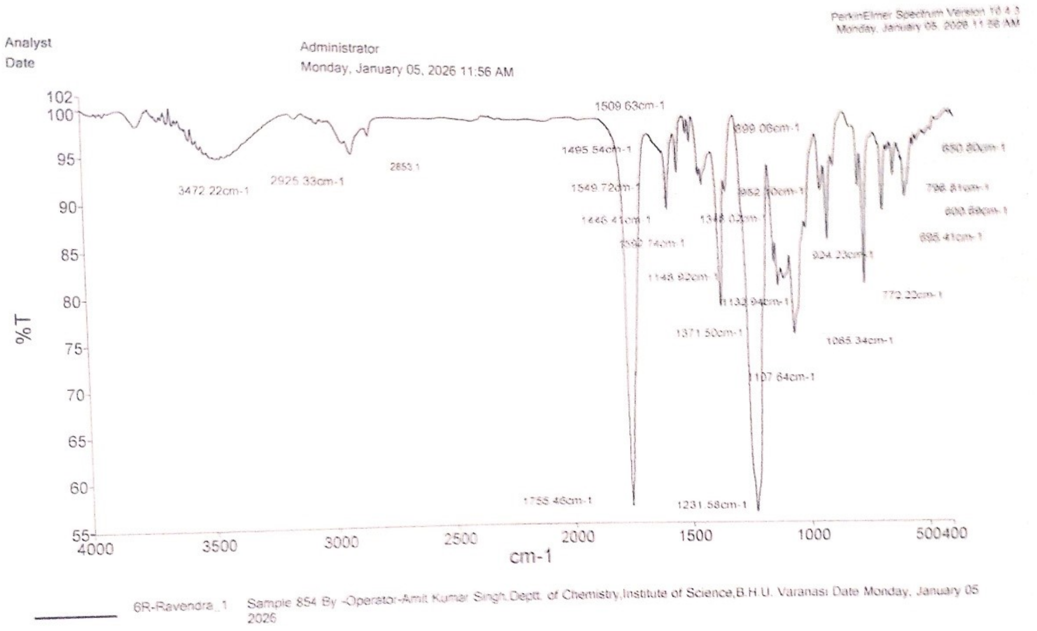

Fig. S42: FT-IR spectrum of **6f**

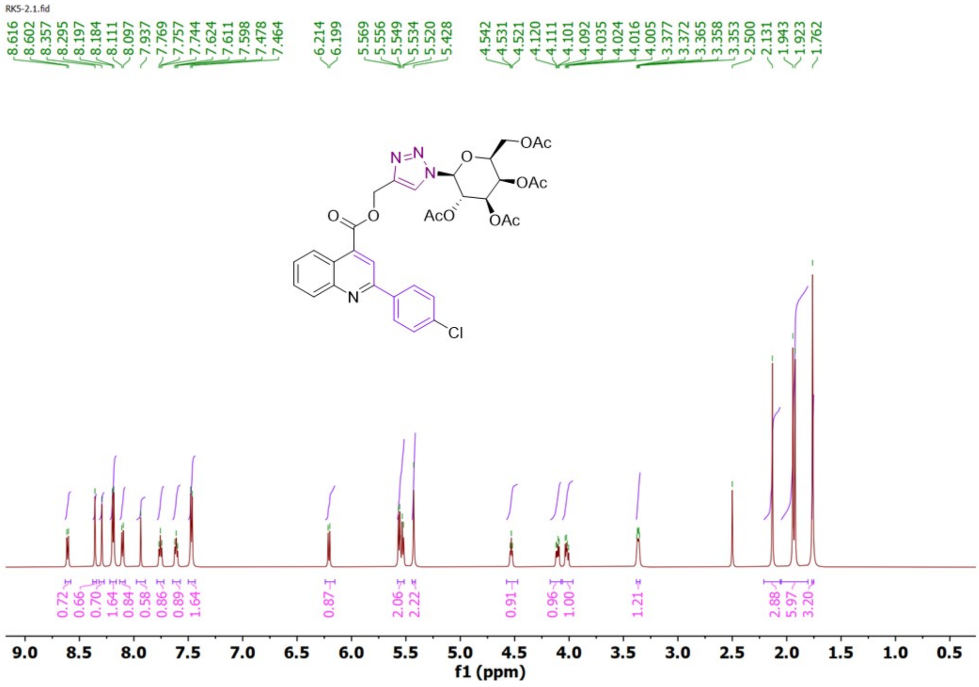

Fig. S43: ^{1}H NMR spectrum of **6g**

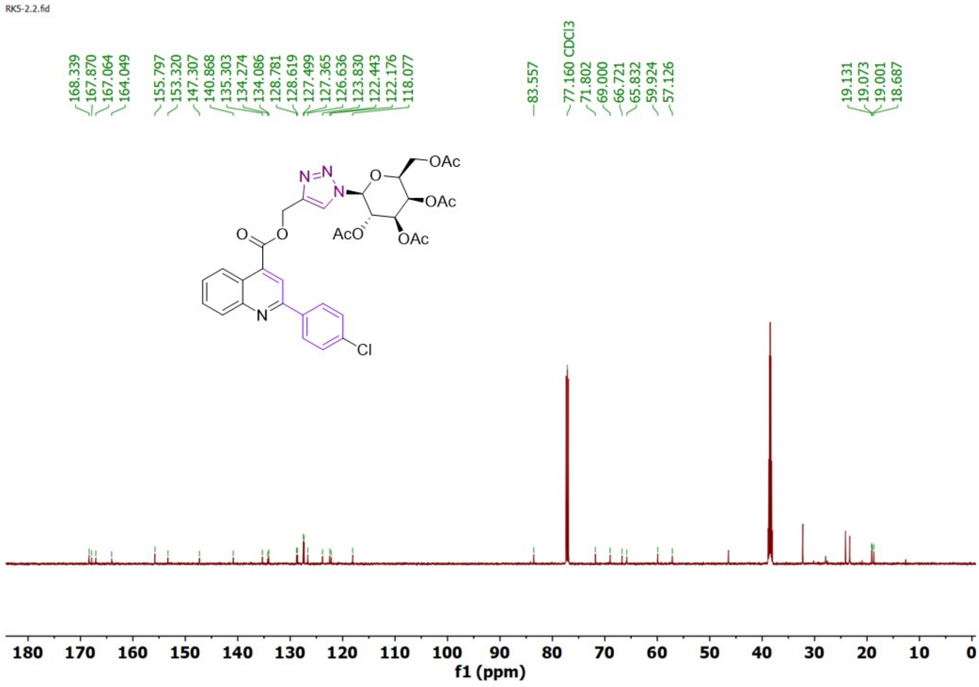

Fig. S44: ^{13}C NMR spectrum of **6g**

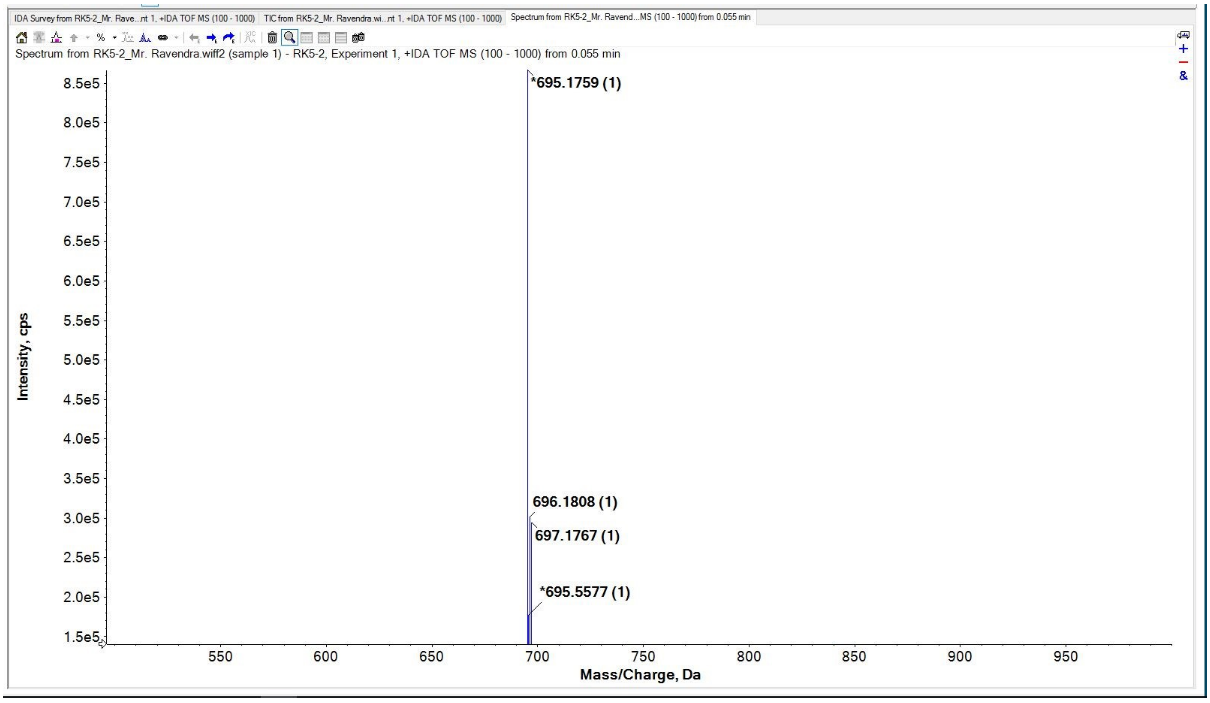

Fig. S45: HRMS spectrum of **6g**

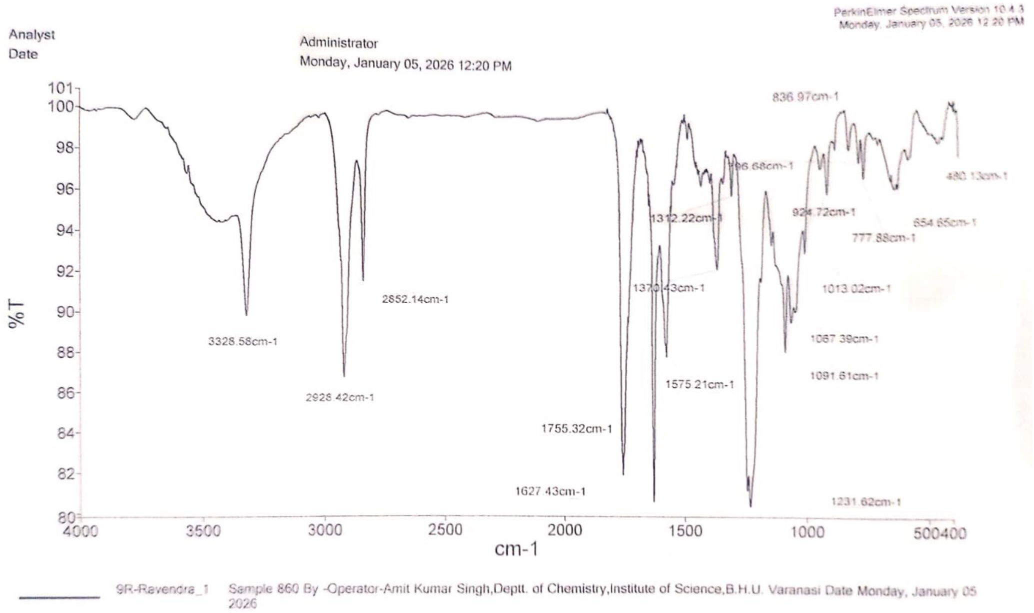

Fig. S46: FT-IR spectrum of **6g**

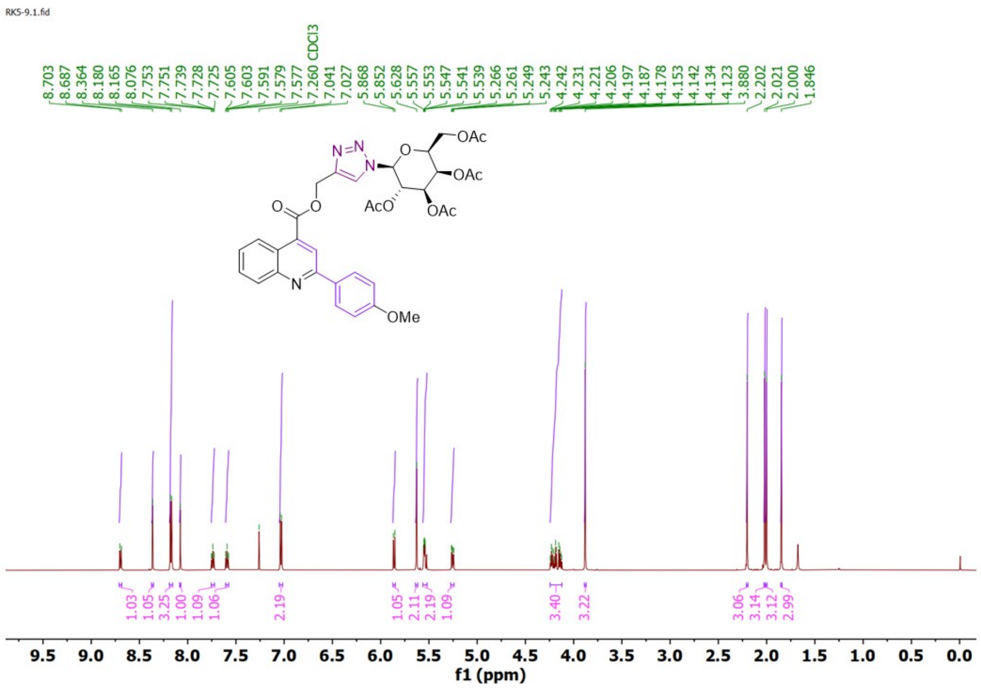

Fig. S47: ^{1}H NMR spectrum of **6h**

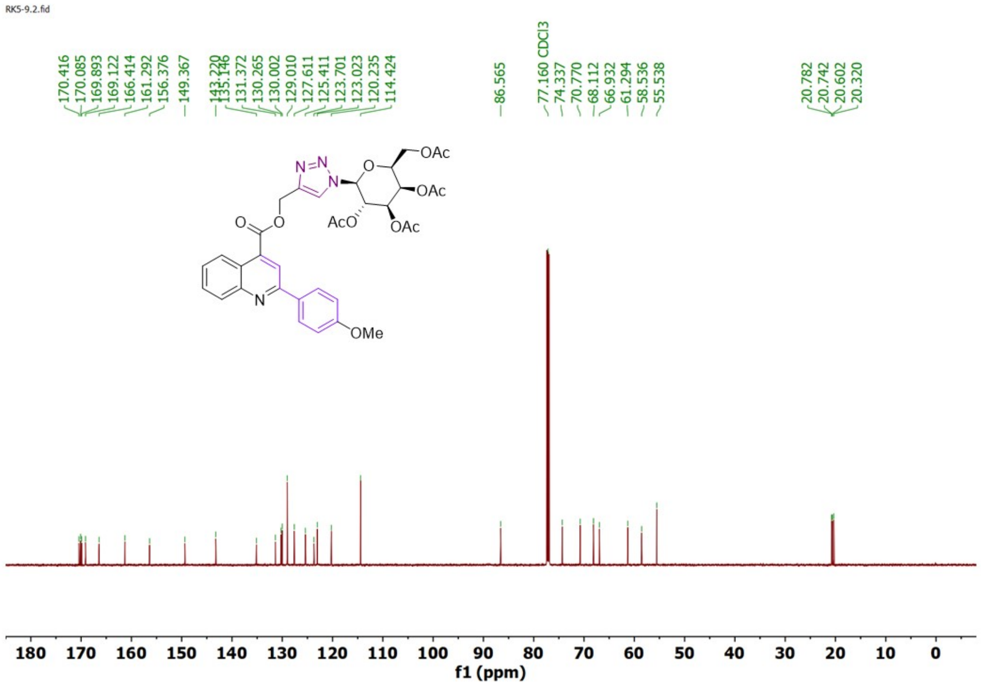

Fig. S48: ^{13}C NMR spectrum of **6h**

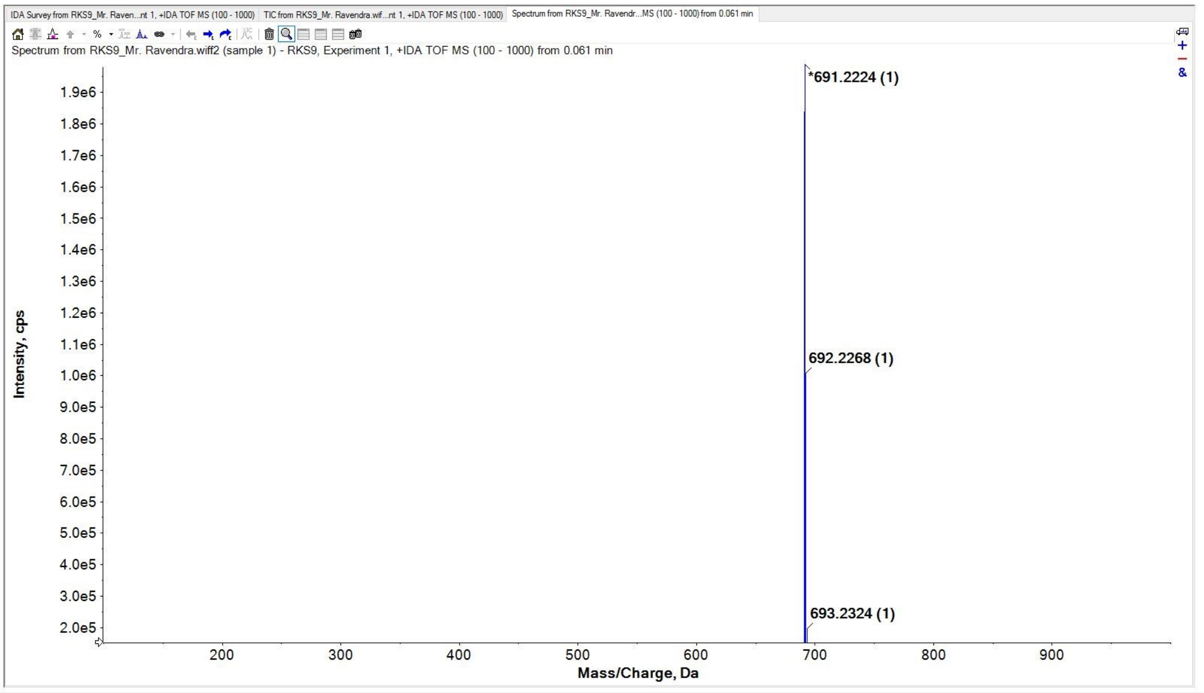

Fig. S49: HRMS spectrum of **6h**

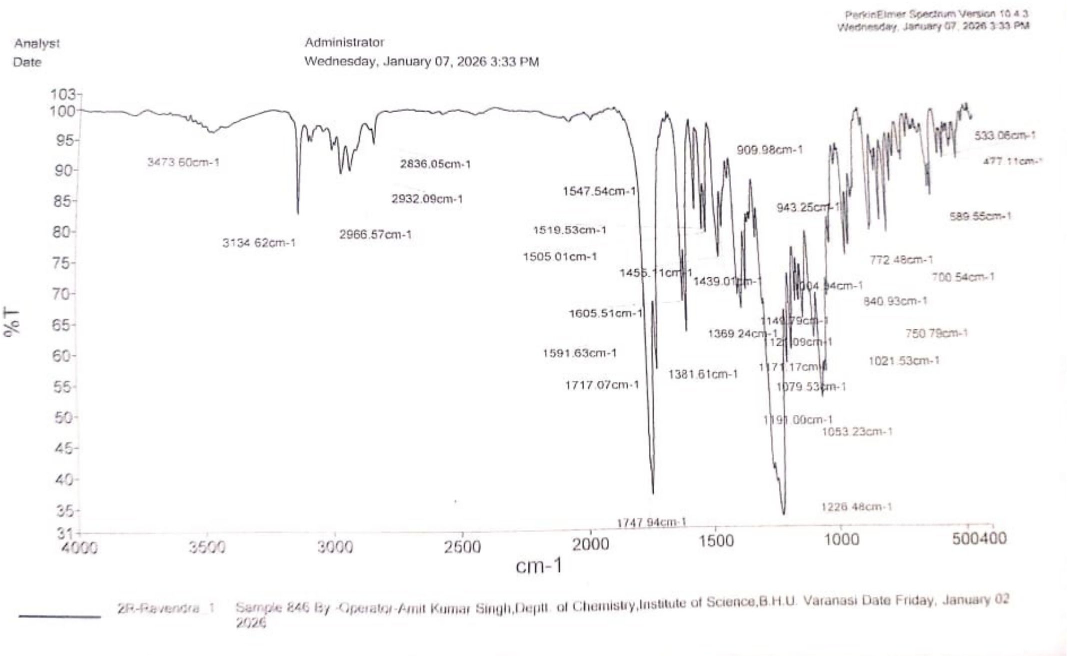

Fig. S50: FT-IR spectrum of **6h**

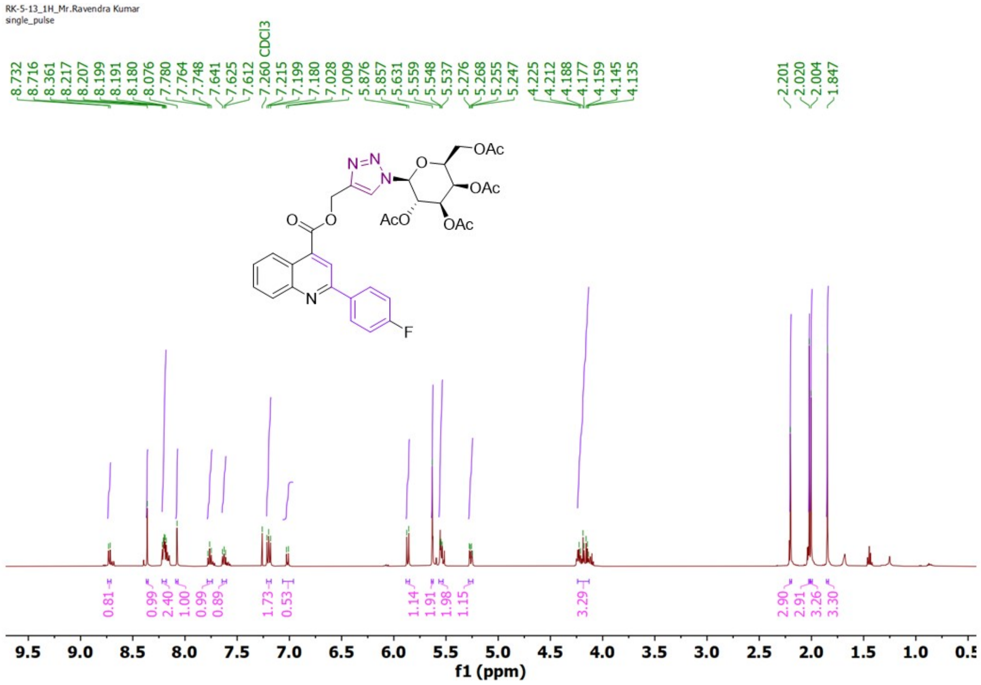

Fig. S51: ^{1}H NMR spectrum of **6i**

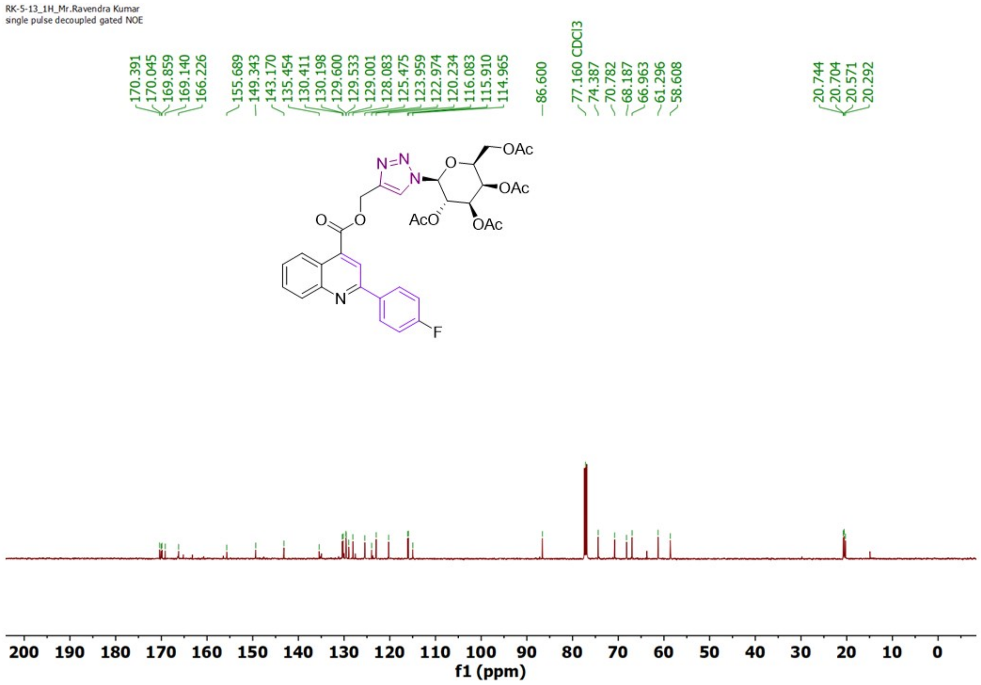

Fig. S52: ^{13}C NMR spectrum of **6i**

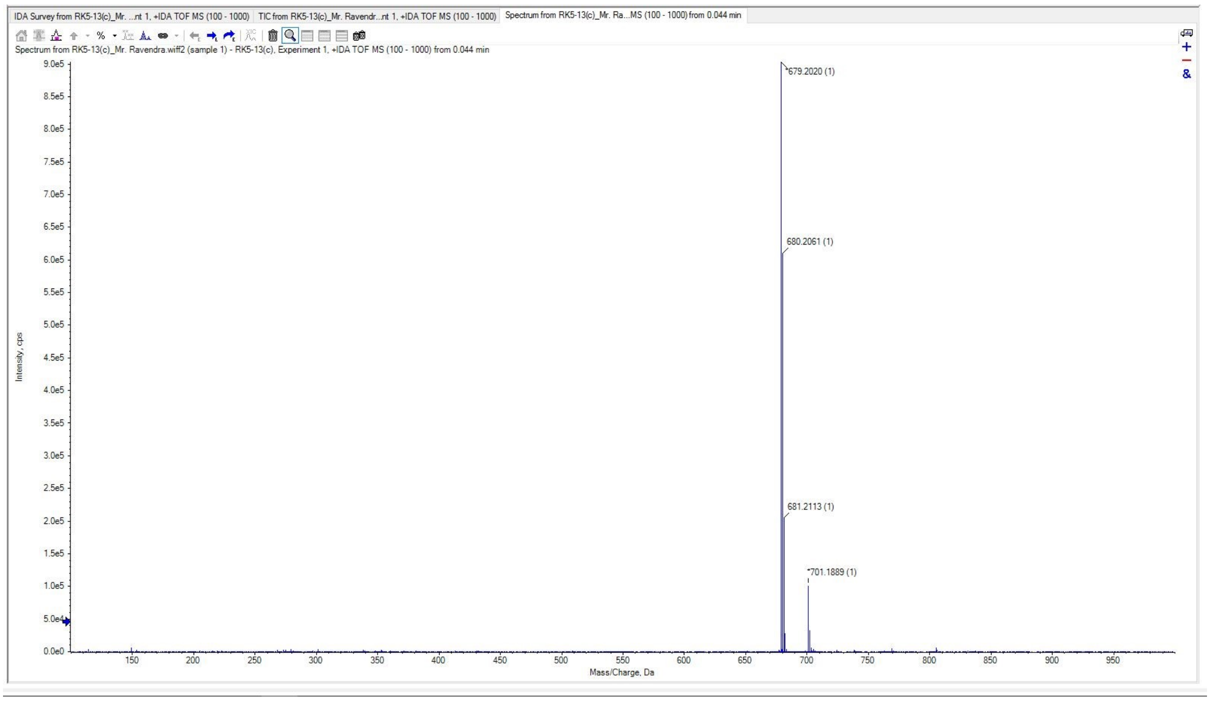

Fig. S53: HRMS spectrum of **6i**

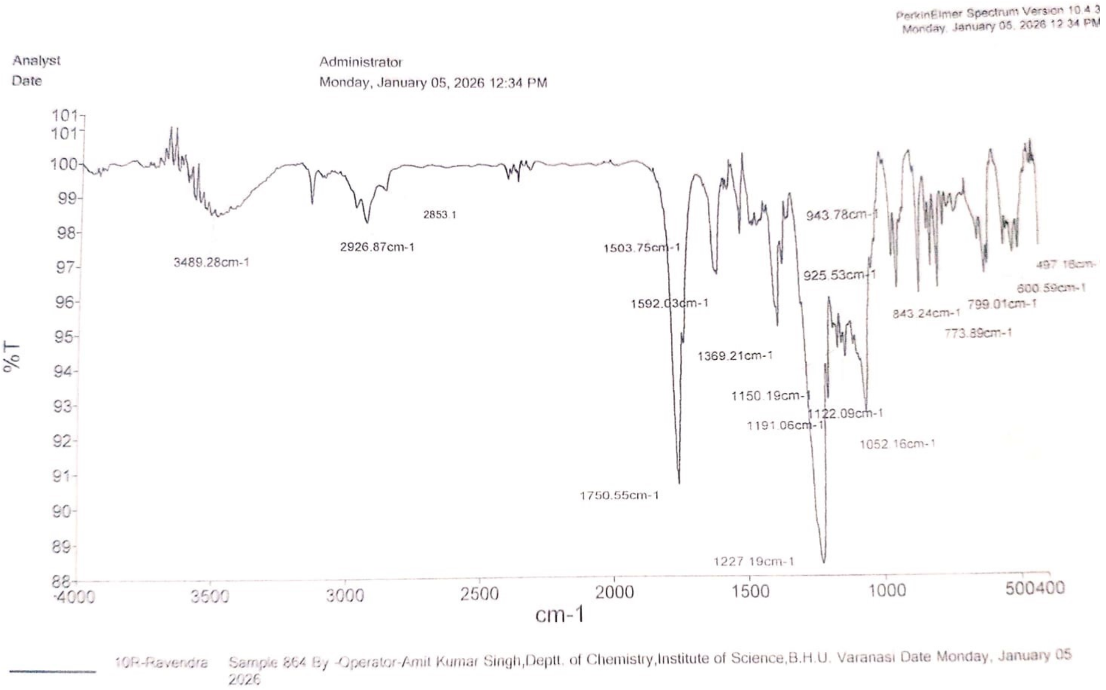

Fig. S54: FT-IR spectrum of **6i**

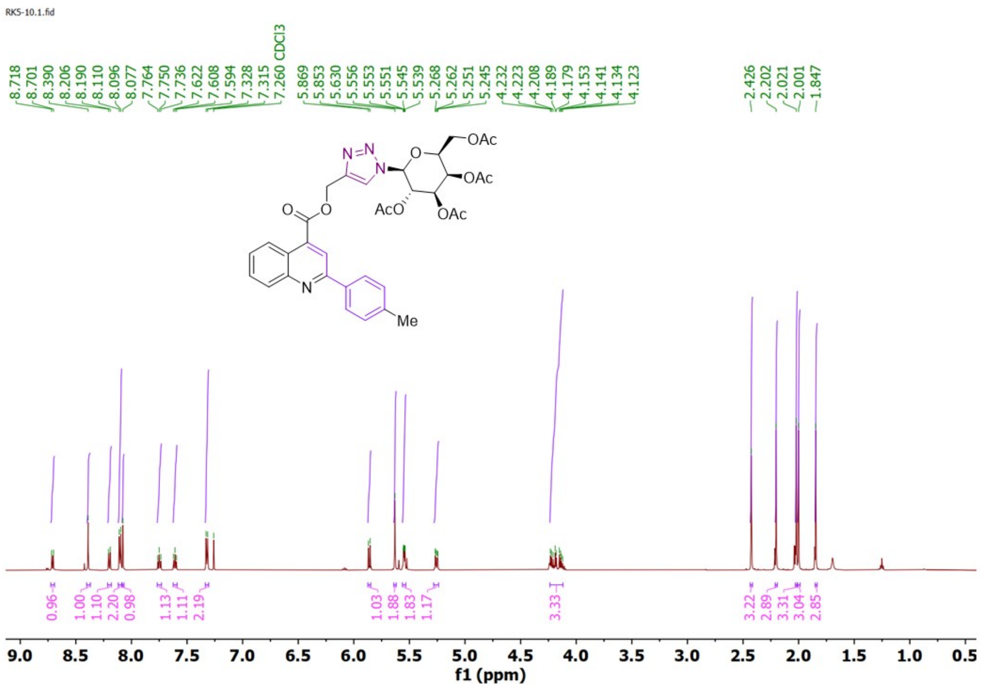

Fig. S55: ^{1}H NMR spectrum of **6j**

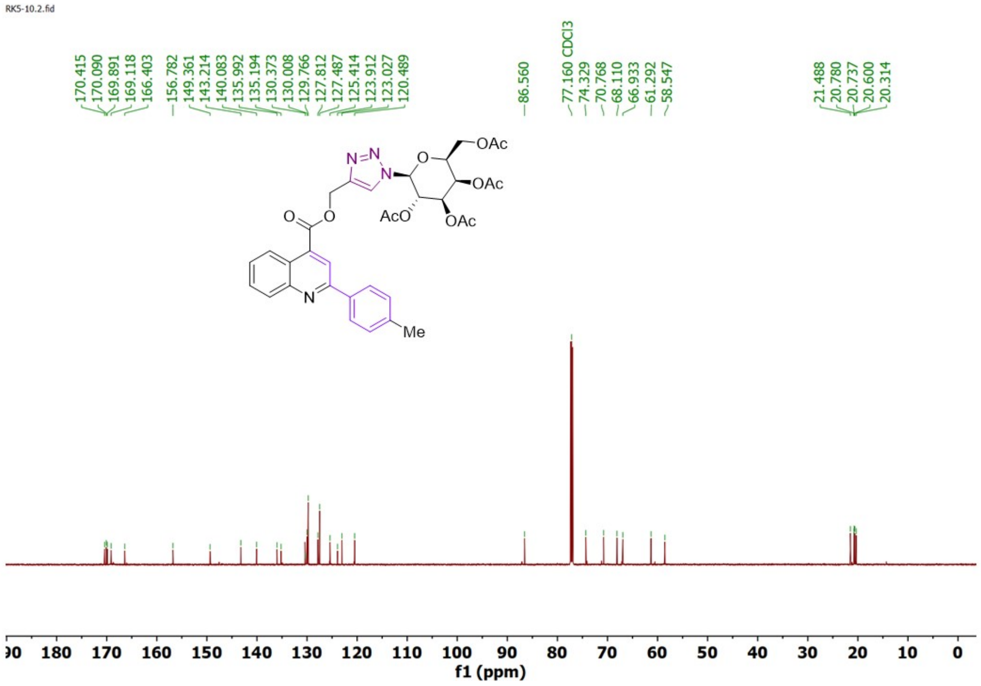

Fig. S56: ^{13}C NMR spectrum of **6j**

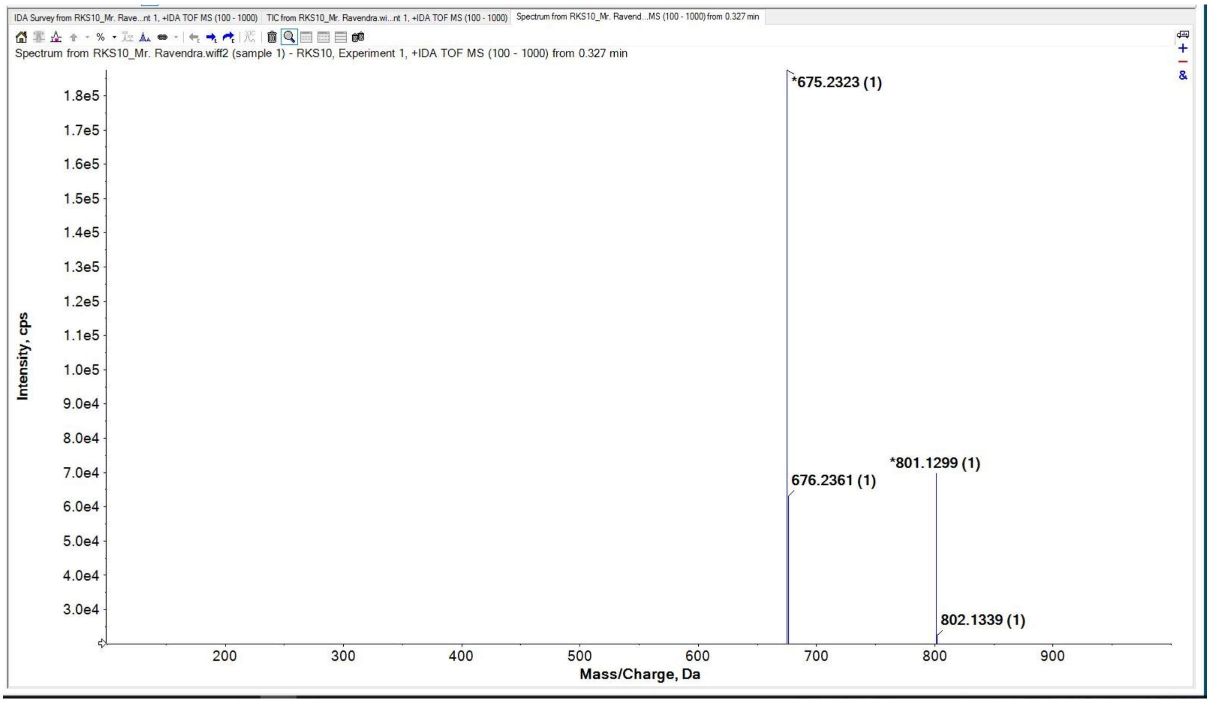

Fig. S57: HRMS spectrum of **6j**

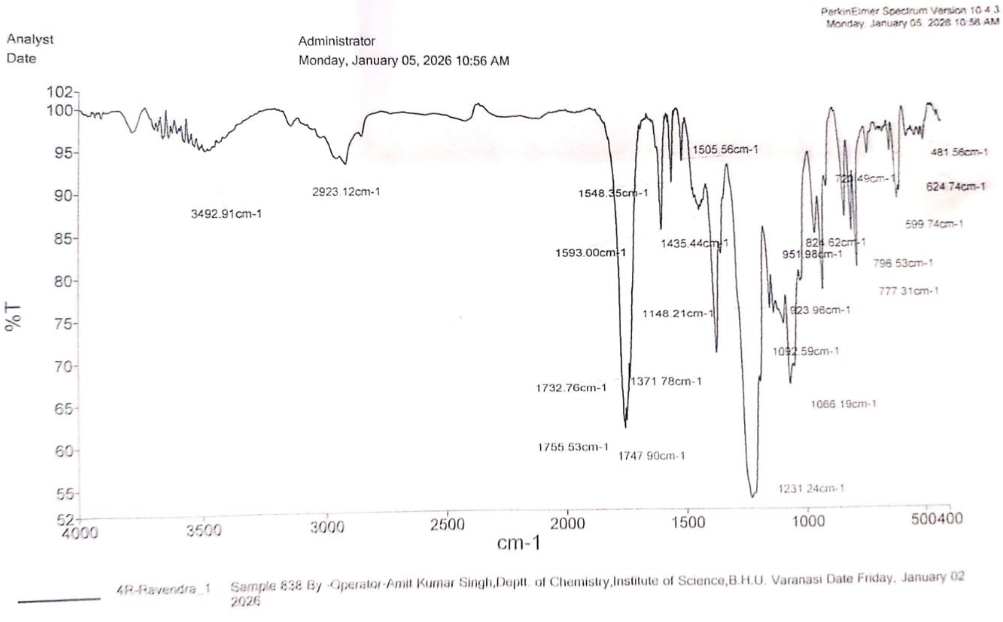

Fig. S58: FT-IR spectrum of **6j**

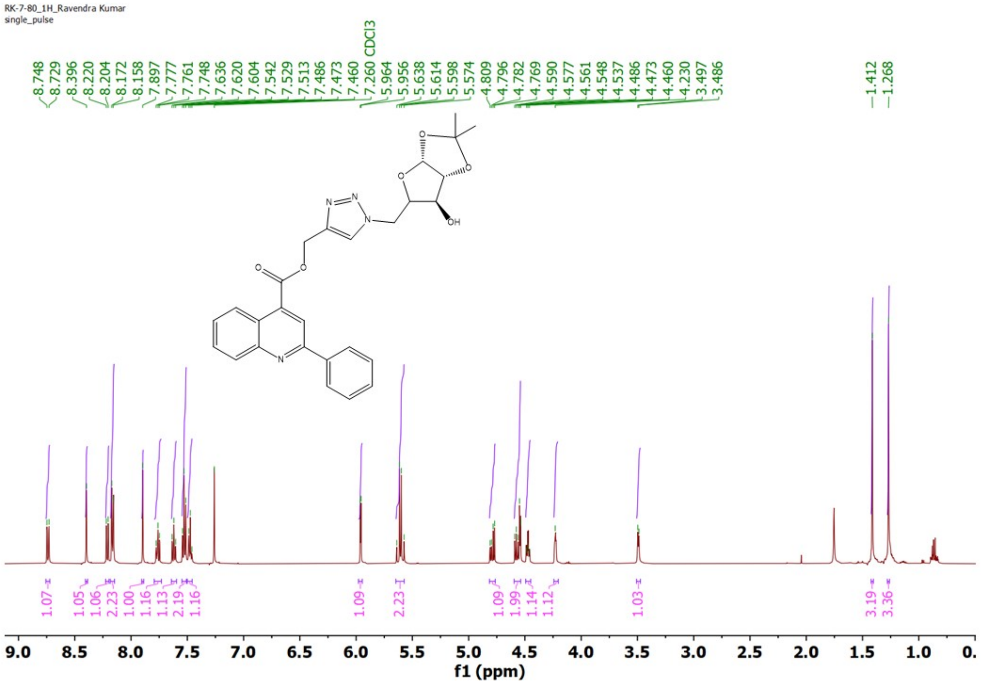

Fig. S59: ^{1}H NMR spectrum of **6K**

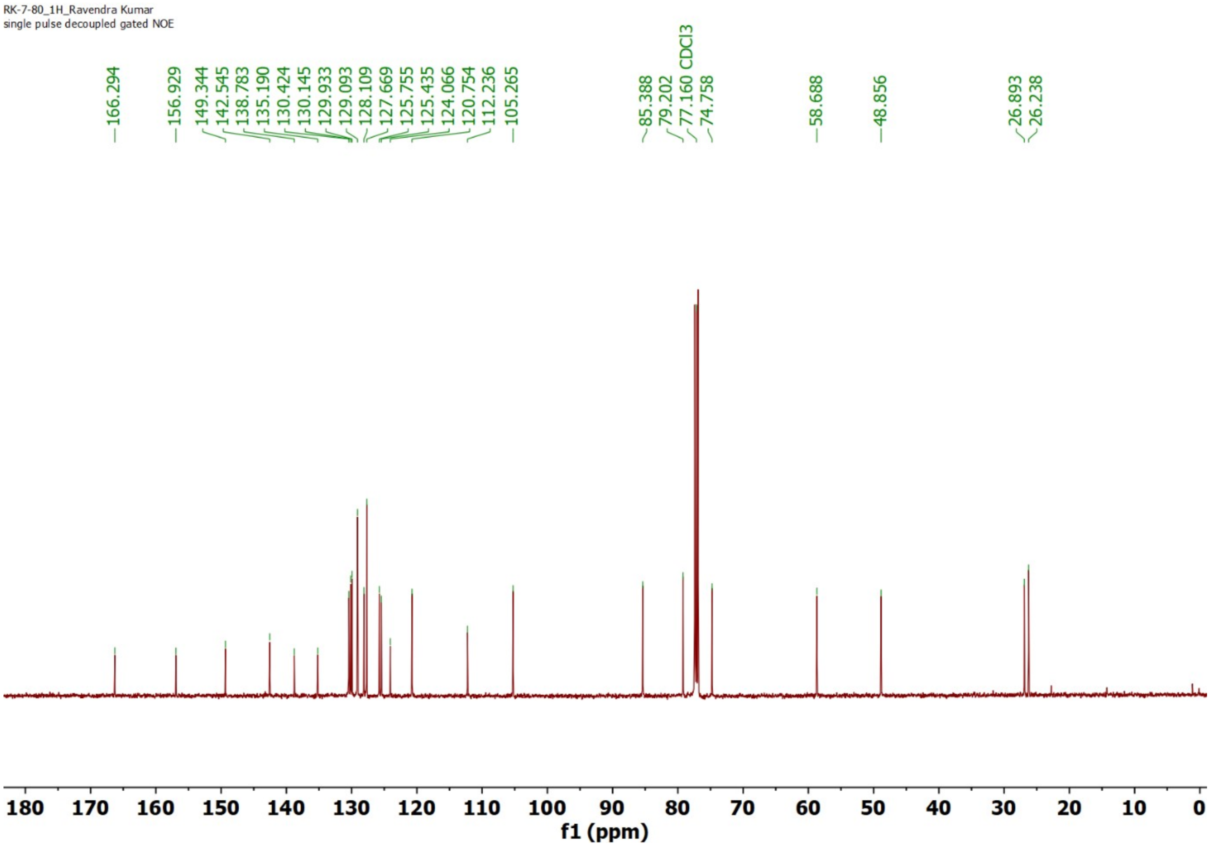

Fig. S60: ^{13}C NMR spectrum of **6K**

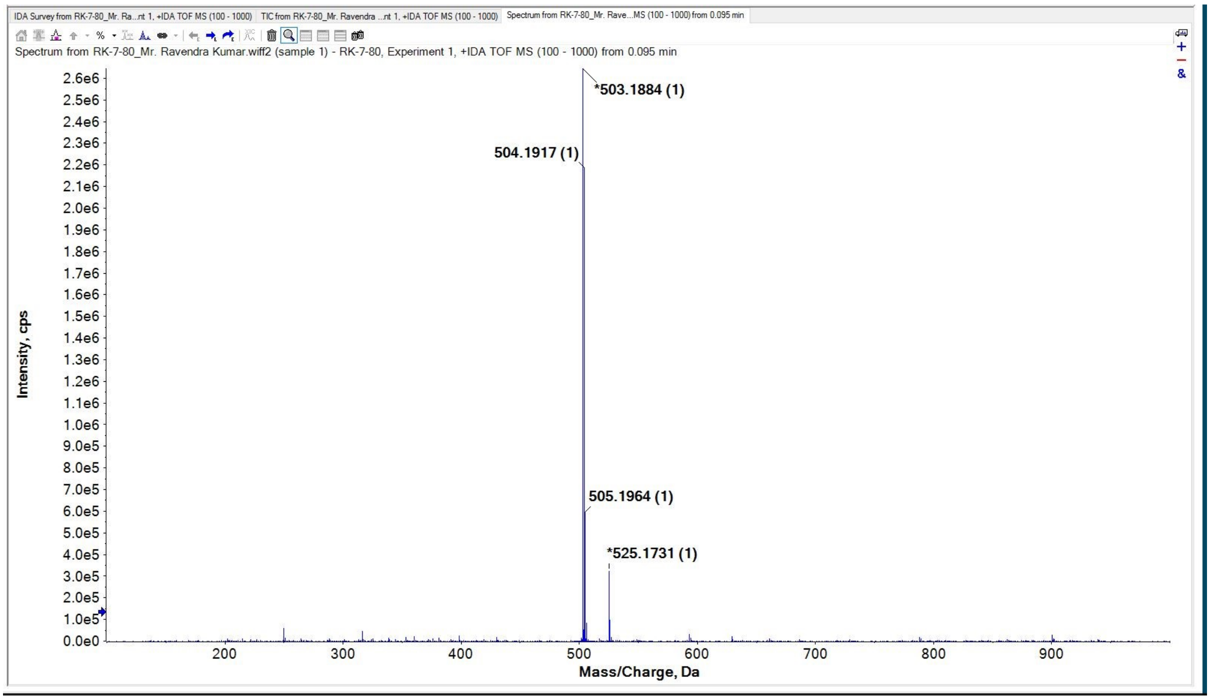

Fig. S61: HRMS spectrum of **6K**

2.0 Crystal structure Intraction

Table S1: Intermolecular C–H, N–H---N, C–H---N, and C–H---O interactions in compound **6a** and **6b** and **6e.**

6a		6b		6e	
Interaction	Distance(Å)	Interaction	Distance(Å)	Interaction	Distance(Å)
sp^2C–N…H(C sp^3 sugar)	2.816	sp^2C–H…O(Csp^2)	2.708	sp^2C–H…O(Csp^2)	2.709
sp^2C–N…H(Csp^3 sugar)	2.807	sp^2C–N…H(Csp^3)	2.800	sp^2C–N…H(Csp^2)	2.778
sp^2C–N…H(Csp^2)	2.705	sp^2C–N…H(Csp^3)	2.840	sp^2C–N…H(Csp^3)	2.806
sp^3C–H…O(C sp^3)	2.674	sp^2C–N…H(Csp^3)	2.786	sp^3C–H…O(Csp^3 sugar)	2.425
sp^3C–H…O(C sp^3)	2.462	sp^3C–H…O(Csp^3 sugar)	2.901	sp^3C–H…O(Csp^3 sugar)	2.528
sp^3C–H…O(C sp^2)	2.561	Sp^3C–H…O(C sp^2)	2.947	sp^3C–H…O(Csp^3 sugar)	2.468
sp^3C–H…O(C sp^2)	2.466	Sp^3C–H…O(C sp^2)	2.494	sp^3C–H…O(Csp^3 sugar)	2.408
sp^3C–H…O(C sp^2)	2.953	Sp^3C–H…O(C sp^2)	2.590	-	-
-	-	Sp^3C–H…O(C sp^2)	2.484	-	-

3.0 Molecular Docking

Table S2. Docking scores (free binding energies in kcal/mol) of compounds **6c, 6b, 6j,** and co-crystalized ligand 4-Hydroxy tamoxifen into the ERα active site (PDB: 3ERT).

Compounds	Binding energy (kcal/mol)	C-H bond/Hydrogen bond	π- alkyl, π- anion, π- sigma, π- sulphur Amide- π- stacked	Vander waals interaction
**6c**	–9.1	Leu536	Leu525,Ala350, Leu346,Asp351 Cys530	Leu384,Met343,Thr347,Tyr526, Trp383,Met522,Leu354,Met528 , Pro535,Glu380,Leu539,Val534
**6f**	–8.9	Leu536,Trp383	Lys529,Leu525, Ala350,Asp351	Leu346,Leu384,Met522,Tyr526, Thr347,Leu353,Met528,Cys530, Glu380,Tyr537,Pro535,Val533, Val534
**6d**	–9.5	Arg394	Met421,Leu346, Leu391,Met343, Leu525	Leu384, Met388, Leu349, Met522, Phe404, Tyr526, Leu354, Leu536, Asp351, Pro535, Val533, Val534, Met528, Thr347, His524, Gly420, Ile424, Ala350, Trp383
**4-Hydroxy Tamoxifen (Control)**	–9.7	Arg394,Glu353, Asp351,	Met421,Leu525, Ala350,Leu346, Leu387,Met343	Phe404,Ile424,Leu428,Met388, Leu391Gly521,His524,Leu384, Thr347,Gly420,Glu419,Trp383, Leu349

*Control: Co-crystallized ligand (original pose)

3.1. Molecular Docking Analysis

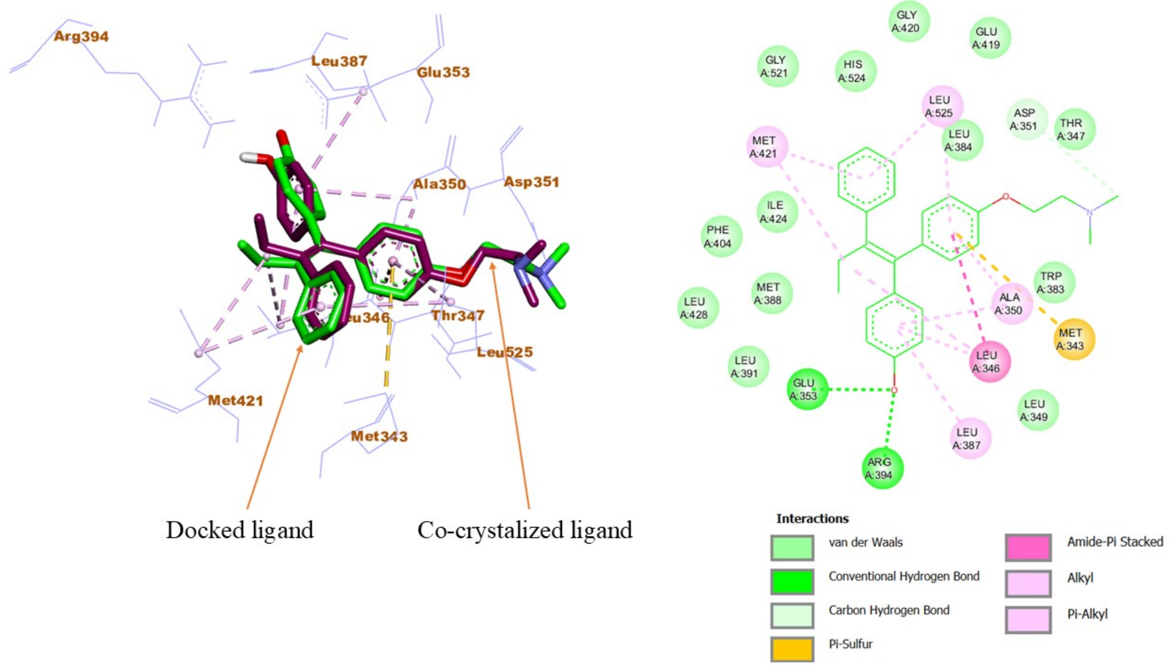

Fig. S48. Validation pose and redocking 3d and 2d pose with the 4-hydrxoy tamoxifen (control) (RMSD-1.541 Å)

4.0 HirshField surface (HS) Analysis

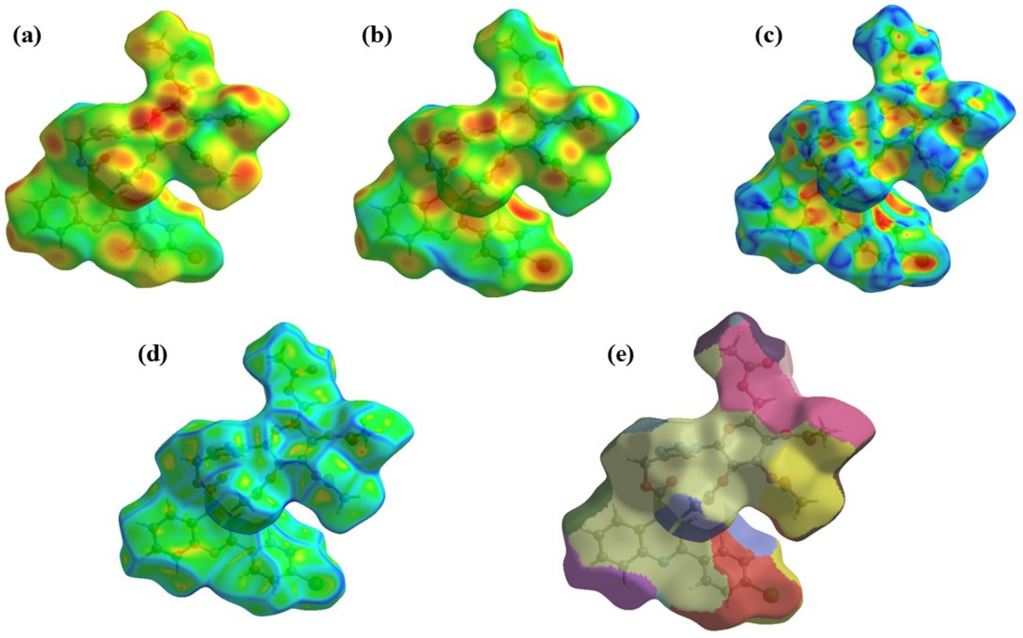

Fig. S49. Hirshfeld surface graph for compound **6b**

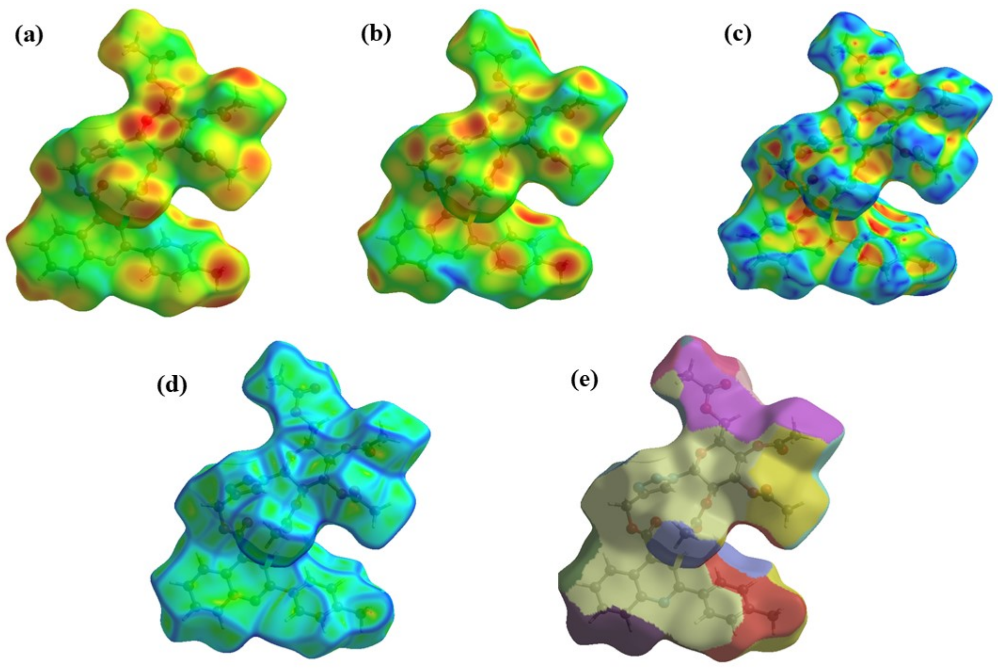

Fig. S50. Hirshfeld Surface graph for compound **6e**

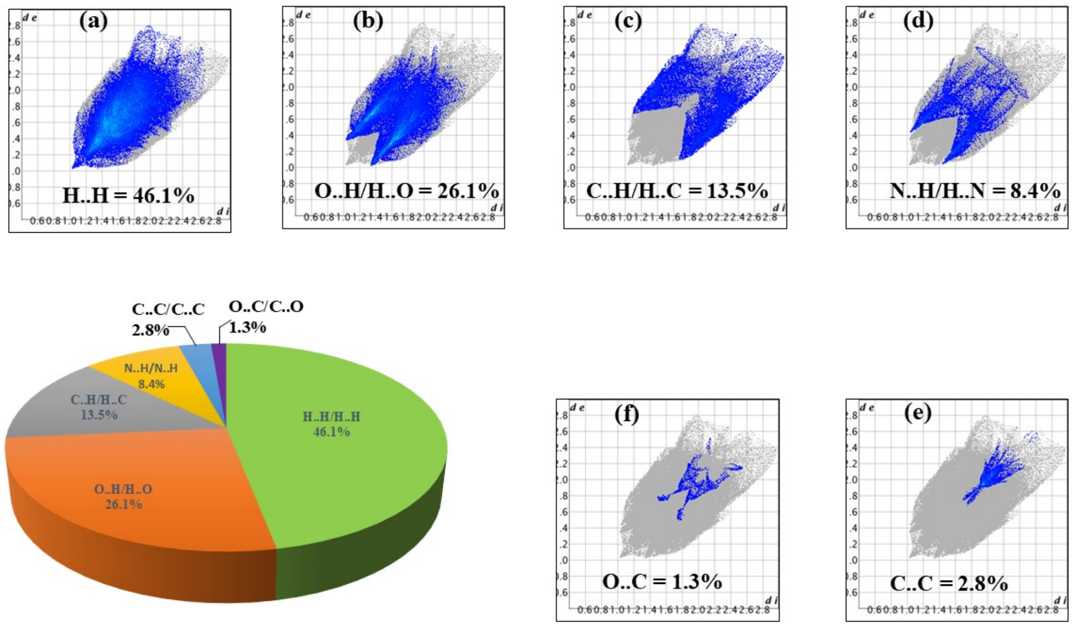

Fig. S51. 2D finger plot % contribution graph for compound **6a**

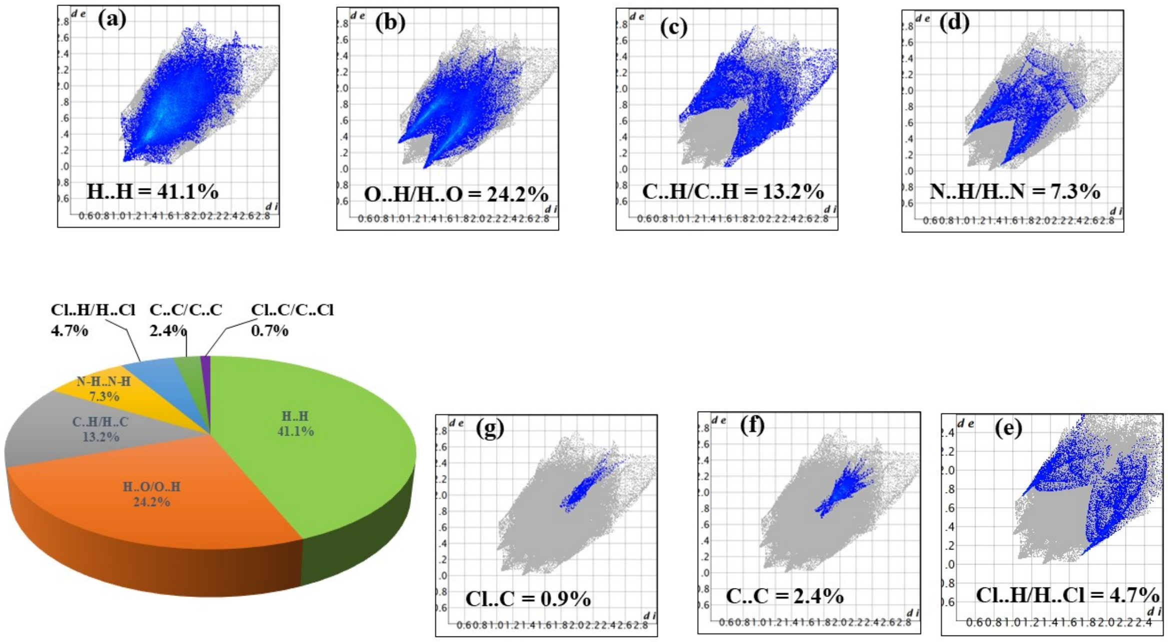

Fig. S52. 2D finger plot % contribution graph for compound **6b**

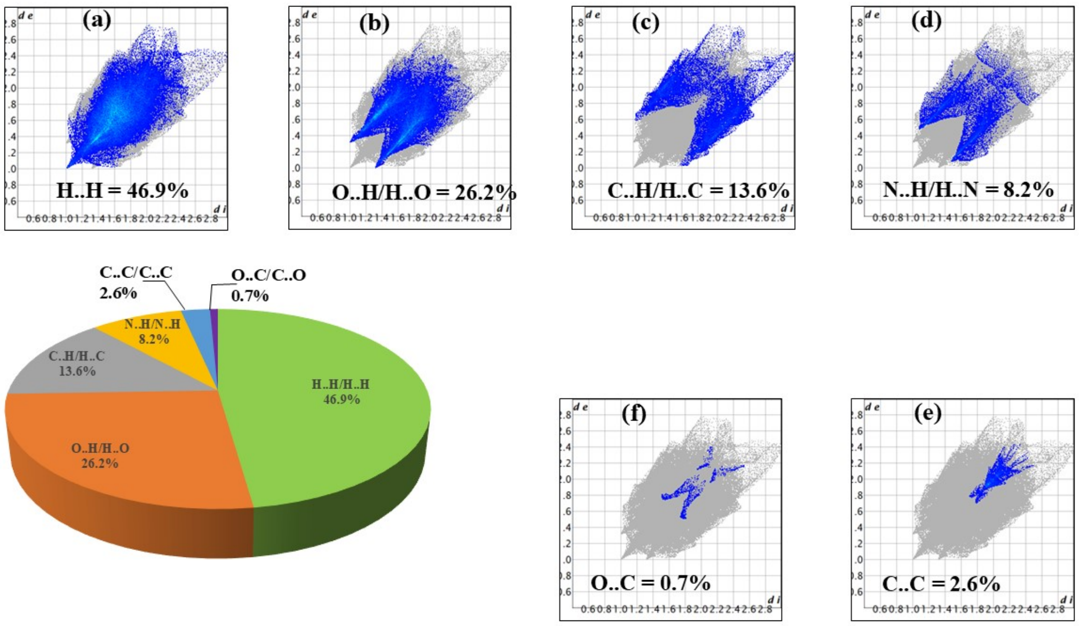

Fig. S53. 2D finger plot % contribution graph for compound **6e**

5.0 DFT Study

5.1 Thermochemical parameter

Table S3. Calculated thermochemical and energetics parameters for the ground state optimized geometrical (in gas phase) of highest binding affinity compounds **6a**, **6b** and **6e** at B3LYP/6-31G (d, p) level of theory.

S/No.	Property/ Parameter	Compounds		
		6a	6b	6e
1	Molecular mass(a.m.u)	660.2068	694.1678	674.2224
2	ZPVE[a](kcal/mol)	391.4593	385.2141	408.5889
3	Zero point correction[b](Hartree/particle)	0.6238	0.6139	0.6511
4	Thermal correction to energy[c](Hartree/particle)	0.6684	0.6597	0.6976
5	Thermal correction to Enthalpy[d](Hartree/particle)	0.6693	0.6607	0.6986
6	Thermal correction to Gibbs free energy[e](Hartree/particle)	0.5387	0.5246	0.5628
7	Sum of electronic and zero-point energies[f] (a.u)	-2322.71	-2782.30	-2362.01
8	Sum of electronic and thermal energies[g](a.u)	-2322.67	-2782.26	-2361.96
9	Sum of electronic and thermal enthalpies[h](a.u)	-2322.67	-2782.26	-2361.96
10	Sum of electronic and thermal free energies[i](a.u)	-2322.80	-2782.39	-2362.10
11	E_{tot}[j] (kcal/mol)	419.429	413.975	437.756
12	S_{tot}[k](cal/mol-kelvin)	275.04	286.332	285.821
13	CV_{tot}[l](cal/mol-kelvin)	180.604	168.598	164.523

5.2 NBO analysis

Table S4. Second order perturbation theory analysis of Fock matrix in NBO basis of compound **6a**, $E^{(2)}$ means energy of hyper conjugative interactions (stabilization energy in kcal/mol). E(j) – E(i) is energy difference between donor and acceptor i and j NBO orbitals. F(i,j) is the Fock matrix element between i and j NBO orbitals.

S. No.	Donor Orbital	Acceptor Orbital	$E^{(2)}$ (kcal/mol)	E(j)-E(i) (a.u.)	F(i,j) (a.u.)
1	C1–C6(π)	C2–C3(π*)	17.33	0.29	0.064
2	C1–C6(π)	C4–C5(π*)	16.78	0.28	0.064
3	C2–C3(π)	C1–C6(π*)	17.39	0.29	0.064
4	C2–C3(π)	C4–C5(π*)	17.38	0.28	0.065
5	C4–C5(π)	C1–C6(π*)	16.36	0.28	0.065
6	C4–C5(π)	C2–C3(π*)	14.71	0.28	0.062
7	C4–C5(π)	N7–C8(π*)	14.9	0.26	0.058
8	C4–C5(π)	C9–C10(π*)	17.48	0.27	0.065
9	N7–C8(π)	C4–C5(π*)	20.8	0.33	0.078

10	C9–C10(π)	N7–C8(π*)	21.13	0.29	0.07
11	C11–C12(π)	C13–C14(π*)	19.95	0.28	0.067
12	C11–C12(π)	C15–C16(π*)	19.56	0.28	0.067
13	C13–C14(π)	C11–C12(π*)	20.53	0.28	0.069
14	C15–C16(π)	C11–C12(π*)	19.44	0.28	0.067
15	C15–C16(π)	C13–C14(π*)	20.99	0.28	0.068
16	C21–C25(π)	N22–N23(π*)	27.51	0.24	0.077
17	N22–N23(π)	C21–C25(π*)	16.49	0.37	0.073
	**Total energy π ⟶ π***		**297.74**	**4.86**	**1.143**
18	O18 LP	C10–C17(σ*)	17.81	0.68	0.101
19	O18 LP	C17–O19(σ*)	31.93	0.64	0.129
25	O34 LP	O33–C35(σ*)	35.43	0.62	0.133
26	O34 LP	C35–C36 (σ*)	18.67	0.64	0.101
29	O38 LP(2)	O37–C39(σ*)	36.81	0.6	0.135
30	O38 LP	C39–C40(σ*)	18.1	0.65	0.1
33	O42 LP	O41–C43(σ*)	35.08	0.62	0.133
34	O42 LP	C43–C44(σ*)	18.71	0.64	0.101
37	O46 LP	O45–C47(σ*)	34.3	0.62	0.132
38	O46 LP	C47–C48(σ*)	17.7	0.65	0.098
	**Total energy LP⟶ σ***		**264.54**	**6.36**	**1.163**
20	O19 LP	C17–O18 (π*)	49.69	0.33	0.115
21	N24 LP(1)	C21–C25 (π*)	35.85	0.3	0.096
22	N24 LP(1)	N22–N23(π*)	43.86	0.24	0.093
23	O33 LP	O34–C35(π*)	46.12	0.33	0.111
27	O37 LP	O38–C39(π*)	42.66	0.34	0.108
28	O38 LP	RY*(1) C39	17.3	1.43	0.141
31	O41 LP	O42–C43(π*)	47.29	0.33	0.112
32	O42 LP	RY*(1) C43	16.76	1.42	0.138
35	O45 LP	O46–C47(π*)	48.21	0.33	0.113
	**Total energy**		**347.74**	**5.05**	**1.027**

	LP⟶ π*				
39	C4–C5 π*	C1–C6(π*)	179.23	0.01	0.078
40	C4–C5 π*	C2–C3(π*)	170.87	0.02	0.078
41	N7–C8 π*	C9–C10(π*)	227.65	0.01	0.083
42	N7–C8 π*	C11–C12(π*)	65.98	0.03	0.062
43	C17–O18 π*	C9–C10(π*)	45.09	0.03	0.067
44	N22–N23 π*	C21–C25(π*)	41.39	0.06	0.068
	**Total energy π*⟶ π***		**730.21**	**0.16**	**0.436**

Table S5. Second order perturbation theory analysis of Fock matrix in NBO basis of compound **6b**, $E^{(2)}$ means energy of hyper conjugative interactions (stabilization energy in kcal/mol). E(j) – E(i) is energy difference between donor and acceptor i and j NBO orbitals. F(i,j) is the Fock matrix element between i and j NBO orbitals.

S. No.	Donor Orbital	Acceptor Orbital	$E^{(2)}$ (kcal/mol)	E(j)-E(i) (a.u.)	F(i,j) (a.u.)
1	C1–C6 (π)	C2–C3(π*)	17.25	0.29	0.064
2	C1–C6(π)	C4–C5(π*)	16.99	0.28	0.064
3	C2–C3(π)	C1–C6(π*)	17.37	0.3	0.064
4	C2–C3(π)	C4–C5(π*)	17.55	0.28	0.065
5	C4–C5(π)	C1–C6(π*)	16.13	0.28	0.065
6	C4–C5(π)	C2–C3(π*)	14.51	0.29	0.061
7	C4–C5 (π)	N7–C8(π*)	15.46	0.26	0.059
8	C4–C5 (π)	C9–C10(π*)	17.5	0.27	0.065
9	N7–C8 (π)	C4–C5(π*)	20.57	0.33	0.077
10	C9–C10 (π)	N7–C8 (π*)	21.19	0.29	0.07
11	C9–C10 (π)	C12–O13(π*)	22.51	0.26	0.069
12	C11–C21 (π)	N7–C8 (π*)	17.25	0.26	0.06
13	C11–C21(π)	C17–C18(π*)	19.64	0.28	0.068
14	C11–C21(π)	C19–C20 (π*)	21.79	0.26	0.068
15	C16–C22(π)	N24–N25(π*)	28.32	0.24	0.078
16	C17–C18(π)	C11–C21(π*)	18.91	0.28	0.066

17	C17–C18(π)	C19–C20(π*)	21.48	0.27	0.068
18	C19–C20(π)	C11–C21 (π*)	18.88	0.3	0.068
19	C19–C20 (π)	C17–C18(π*)	17.94	0.3	0.066
	**Total energy π ⟶ π***		**361.24**	**5.32**	**1.805**
20	LP(2) O13	C10–C12(σ*)	17.81	0.68	0.1
21	LP(2) O13	C12–O14 (σ*)	31.34	0.64	0.128
30	LP(2) O40	O37–C38 (σ*)	36.02	0.61	0.134
31	LP(2) O40	C38–C39 (σ*)	18.61	0.64	0.1
33	LP(2) O43	O35–C41 (σ*)	35.4	0.62	0.133
34	LP(2) O43	C41–C42 (σ*)	18.6	0.65	0.1
36	LP(2) O46	O36–C44 (σ*)	34.13	0.62	0.132
37	LP(2) O46	C44–C45(σ*)	18.59	0.65	0.101
38	LP(2) O48	O34–C47(σ*)	38.58	0.58	0.135
39	LP(2) O48	C47–C49(σ*)	18.74	0.62	0.099
	**Total energy LP⟶ σ***		**267.82**	**6.31**	**1.162**
22	LP(2) O14	C12–O13 (π*)	51.58	0.32	0.116
23	LP(1) N23	C16–C22 (π*)	35.95	0.3	0.097
24	LP(1) N23	N24–N25 (π*)	41.52	0.25	0.091
25	LP(2) O34	C47–O48 (π*)	37.82	0.35	0.103
26	LP(2) O35	C41–O43 (π*)	46.64	0.33	0.112
27	LP(2) O36	C44–O46 (π*)	47.74	0.33	0.113
28	LP(2) O37	C38–O40 (π*)	45.39	0.34	0.111
	**Total energy LP⟶ π***		**306.64**	**2.22**	**0.743**
40	C4–C5(π*)	C1–C6 (π*)	150.84	0.02	0.078
41	C4–C5(π*)	C2–C3 (π*)	148.92	0.02	0.078
42	N7–C8(π*)	C9–C10 (π*)	255.21	0.01	0.084
43	N7–C8(π*)	C11–C21 (π*)	101.72	0.02	0.065
44	C12–O13(π*)	C9–C10 (π*)	52.3	0.03	0.072
45	C19–C20(π*)	C11–C21 (π*)	201.26	0.02	0.084

46	C19–C20 (π*)	C17–C18 (π*)	134.15	0.02	0.079
47	N24–N25(π*)	C16–C22 (π*)	42.89	0.06	0.069
	**Total energy π ⟶ π***		**1087.29**	**0.2**	**0.609**

Table S6. Second order perturbation theory analysis of Fock matrix in NBO basis of compound **6e**, $E^{(2)}$ means energy of hyper conjugative interactions (stabilization energy in kcal/mol). E(j) – E(i) is energy difference between donor and acceptor i and j NBO orbitals. F(i,j) is the Fock matrix element between i and j NBO orbitals.

S. No.	Donor Orbital	Acceptor Orbital	$E^{(2)}$ (kcal/mol)	E(j)-E(i) (a.u.)	F(i,j) (a.u.)
1	C1–C6(π)	C2–C3(π*)	17.37	0.29	0.064
2	C1–C6(π)	C4–C5(π*)	16.73	0.28	0.064
3	C4–C5(π)	C1–C6(π*)	16.44	0.28	0.065
4	C4–C5(π)	C2–C3(π*)	14.71	0.28	0.062
5	C4–C5(π)	N7–C8(π*)	14.85	0.26	0.058
6	C4–C5(π)	C9–C10(π*)	17.47	0.27	0.065
7	N7–C8(π)	C4–C5(π*)	20.91	0.33	0.078
8	N7–C8(π)	C9–C10(π*)	12.32	0.33	0.057
9	C9–C10(π)	C4–C5(π*)	14.42	0.29	0.061
10	C9–C10(π)	N7–C8(π*)	20.99	0.29	0.07
11	C9–C10(π)	C17–O18(π*)	19.45	0.27	0.065
12	C11–C12(π)	N7–C8(π*)	17.08	0.26	0.059
13	C11–C12(π)	C13–C14(π*)	18.98	0.28	0.066
14	C11–C12(π)	C15–C16(π*)	19.91	0.28	0.068
15	C13–C14(π)	C11–C12(π*)	22.07	0.28	0.071
16	C13–C14(π)	C15–C16(π*)	17.88	0.28	0.065
17	C15–C16(π)	C11–C12(π*)	18.51	0.28	0.065
18	C15–C16(π)	C13–C14(π*)	21.55	0.28	0.07
19	C21–C25(π)	N22–N23(π*)	27.54	0.24	0.077
20	N22–N23(π)	C21–C25(π*)	16.47	0.37	0.073
	**Total energy π ⟶ π***		**365.65**	**5.72**	**1.323**

21	LP(2) O18	C10–C17(σ*)	17.77	0.69	0.101
22	LP(2) O18	C17–O19(σ*)	31.9	0.64	0.129
28	LP(2) O34	O33–C35(σ*)	36.79	0.6	0.135
29	LP(2) O34	C35–C36(σ*)	18.09	0.65	0.1
32	LP(2) O38	O37–C39(σ*)	35.07	0.62	0.133
33	LP(2) O38	C39–C40(σ*)	18.71	0.64	0.101
36	LP(2) O42	O41–C43(σ*)	34.32	0.62	0.132
37	LP(2) O42	C43–C44(σ*)	17.73	0.65	0.098
40	LP(2) O47	O46–C48(σ*)	35.41	0.62	0.133
41	LP(2) O47	C48–C49(σ*)	18.68	0.64	0.101
	**Total energy LP⟶ σ***		**264.47**	**6.37**	**1.163**
23	LP(2) O19	C17–O18(π*)	49.64	0.33	0.115
24	LP(1) N24	C21–C25(π*)	35.85	0.3	0.096
25	LP(1) N24	N22–N23(π*)	43.88	0.24	0.093
26	LP(2) O33	O34–C35(π*)	42.67	0.34	0.108
30	LP(2) O37	O38–C39(π*)	47.32	0.33	0.112
34	LP(2) O41	O42–C43(π*)	48.14	0.33	0.113
38	LP(2) O46	O47–C48(π*)	46.15	0.33	0.111
	**Total energy LP⟶ π***		**313.65**	**2.2**	**0.748**
42	C4–C5(π*)	C1–C6(π*)	181.83	0.01	0.078
43	C4–C5(π*)	C2–C3(π*)	173.54	0.02	0.078
44	N7–C8(π*)	C9–C10(π*)	235.88	0.01	0.083
45	N7–C8(π*)	C11–C12(π*)	66.54	0.03	0.063
46	C17–O18(π*)	C9–C10(π*)	44.86	0.03	0.067
47	N22–N23(π*)	C21–C25(π*)	41.3	0.06	0.068
	**Total energy π ⟶ π***		**743.95**	**0.13**	**0.437**
